# Developing therapeutic strategies to target MCL1 and BCLXL in lethal prostate cancer

**DOI:** 10.1016/j.isci.2025.113985

**Published:** 2025-11-10

**Authors:** Daniel Westaby, Juan M. Jiménez-Vacas, Ines Figueiredo, Jonathan Welti, Bora Gurel, Denisa Bogdan, Lorenzo Buroni, Antje J. Neeb, Jan Rekowski, Ana Padilha, Souvik Das, Joe Taylor, Wanting Zeng, Nick Waldron, Thomas Goldsmith, Emily Hobern, Florian Gabel, Nicole Pandell, Susana Miranda, Maryou B. Lambros, Suzanne Carreira, Amanda Swain, Wei Yuan, Steven P. Balk, Marco Bezzi, Johann S. de Bono, Adam Sharp

**Affiliations:** 1 The Institute of Cancer Research, London, UK; 2 The Royal Marsden NHS Foundation Trust, London, UK; 3 Beth Israel Deaconess Medical Center, Boston, MA, USA

**Keywords:** Pharmacology, molecular biology, cancer

## Abstract

Targeting anti-apoptotic BCL2 family proteins is an attractive therapeutic strategy to drive prostate cancer (PCa) cell death. Here, we show that MCL1 is highly expressed in castration-resistant PCa, associating with worse clinical outcome. We demonstrate that targeting MCL1 with BH3 mimetics triggers apoptotic cell death in a subset of PCa cell line models. Furthermore, siRNA targeting of UCHL3, a deubiquitinating enzyme, downregulates MCL1 expression to synergize with BCLXL blockade; however, its impact on MCL1 is driven through an off-target effect, raising an important methodological consideration when studying MCL1 biology. Finally, we demonstrate that co-targeting MCL1 and BCLXL in patient-derived and mouse PCa models drives apoptotic PCa cell death. Taken together, targeting the intrinsic apoptosis pathway remains an attractive therapeutic strategy for lethal PCa. Future studies should focus on identifying strategies and technologies that can deliver cancer specific kill, to improve the outcome for men with this lethal disease.

## Highlights

• MCL1 is highly expressed and associates with poor outcome in lethal prostate cancer (PCa)
• Extensive validation is needed when using siRNA screens to study MCL1 biology
• Targeting MCL1 drives apoptotic death in a subset of PCa cell lines
• Co-targeting MCL1 and BCLXL induces apoptosis in patient-derived and mouse PCa models

## Graphical abstract

**Figure:**
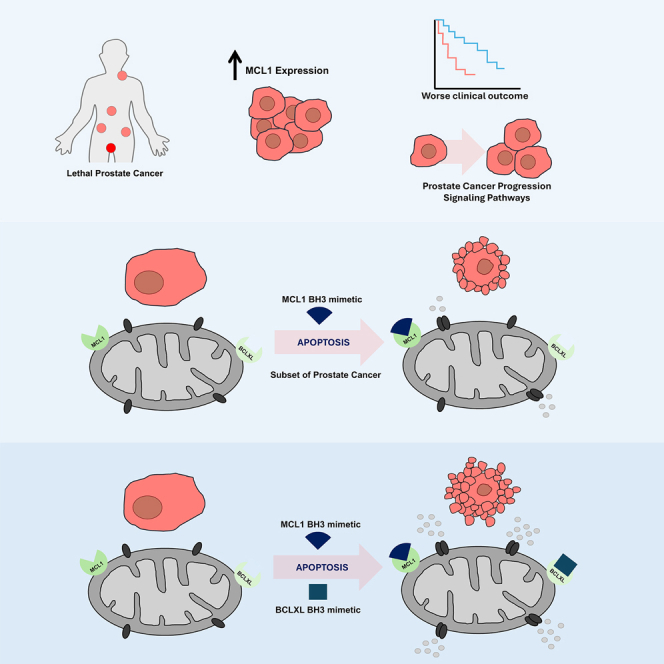

## Introduction

Prostate cancer (PCa) is the commonest cancer, and the second highest cause of cancer related death, in men in the Western world and is increasing in incidence.^1^ Despite the development of multiple new therapies over the past two decades, including taxane chemotherapies, androgen receptor (AR) signaling inhibitors (ARSIs), poly ADP-ribose polymerase (PARP) inhibitors, and radionuclide therapy, treatment resistance is inevitable and advanced PCa remains lethal.^2^^,^^3^^,^^4^ Therefore, the development of innovative therapeutic strategies with novel mechanisms of action is an urgent unmet clinical need in PCa medicine.

The intrinsic apoptosis pathway, which is tightly regulated by pro- and anti-apoptotic BCL2 proteins that interact on the mitochondrial outer membrane, is an attractive area of cancer biology that demonstrates promise as a target for novel therapeutic strategies in lethal PCa.^5^^,^^6^ Pro-apoptotic BCL2 proteins can be subcategorized as BCL2 homology domain (BH3)-only “activators” (BIM, BID, and PUMA), BH3-only “sensitizers” (BAD, NOXA, HRK, BIK, and BMF) and pore-forming “effectors” (BAK and BAX). Counteracting these are anti-apoptotic BCL2 proteins including MCL1, BCL2, and BCLXL, which sequester pro-apoptotic BCL2 family members.^7^ BH3-only “activators” promote apoptosis by directly engaging and activating BAX/BAK, as well as sequestering anti-apoptotic BCL2 proteins. BH3-only “sensitizers” are unable to directly engage BAX/BAK and primarily function by sequestering anti-apoptotic members.^8^ Once activated, BAX/BAK undergo oligomerization and form macropores, leading to mitochondrial outer membrane permeabilization (MOMP), the release of cytochrome *c* and caspase driven apoptotic cell death.^6^ Importantly, the balance between pro- and anti-apoptotic BCL2 family proteins is critical in determining cell fate decisions and responses to therapy. Targeting the anti-apoptotic BCL2 proteins, with small molecule BH3 mimetics, antibody drug conjugates (ADC) and proteolysis targeting chimeras (PROTAC), to breach the apoptotic threshold, is an attractive therapeutic strategy for lethal PCa.^6^^,^^9^^,^^10^^,^^11^^,^^12^^,^^13^^,^^14^^,^^15^^,^^16^^,^^17^^,^^18^^,^^19^^,^^20^^,^^21^ It is now critical to dissect the biology of the intrinsic apoptosis pathway to deliver novel treatments for men suffering from lethal PCa.

In contrast to BCL2, which we have shown to be predominantly expressed in a subset of PCa tumors with lineage plasticity, MCL1 and BCLXL are highly expressed in the majority of PCa tumors, associating with inferior clinical outcomes.^6^^,^^9^^,^^11^^,^^22^ In addition, MCL1 and BCLXL have been shown to reduce the anti-tumour efficacy of AR targeting therapies and chemotherapies in PCa.^6^^,^^12^^,^^13^^,^^14^^,^^16^^,^^18^ Furthermore, the clinical development of drugs targeting the anti-apoptotic BCL2 family proteins, which include the food and drug administration (FDA) approved BH3 mimetic BCL2 inhibitor venetoclax, and novel strategies utilizing ADC and PROTAC approaches, provide exciting opportunities to target intrinsic apoptosis in PCa.^6^^,^^19^^,^^20^ However, clinical translation of these therapeutic strategies for solid malignancies, including lethal PCa, has remained challenging due to the relative functional redundancy between BCL2 family proteins and “on-target” toxicities, supporting the urgent need for innovative approaches.^23^^,^^24^^,^^25^^,^^26^

Here, we report that MCL1 RNA is highly expressed in castration-resistant PCa (CRPC) tissue biopsies and associates with worse clinical outcome. In addition, we show that a number of PCa models are highly dependent on MCL1 function for survival. Moreover, a deubiquitinating enzyme (DUB) siRNA screen reveals that siRNA targeting of UCHL3 decreases MCL1 protein expression and synergizes with BCLXL/BCL2 or BCLXL inhibition to activate the intrinsic apoptosis pathway and drive PCa cell death. However, further interrogation of the UCHL3 siRNA constructs demonstrate that MCL1 protein downregulation is mediated by off-target effects of the UCHL3 siRNA seed region, an important consideration when studying MCL1 biology. Nonetheless, UCHL3 is lost in a significant subset of CRPC tissue biopsies and its expression associates with key signaling pathways. Furthermore, we demonstrate that therapeutic strategies targeting MCL1 (directly or indirectly) synergize with BCLXL/BCL2 blockade, activating the intrinsic apoptosis pathway to drive PCa cell death in patient-derived and mouse PCa models. Taken together, our findings support further interrogation of the intrinsic apoptosis pathway to assist with the development of therapeutic strategies targeting anti-apoptotic BCL2 family proteins, to deliver cancer specific kill for men suffering from lethal PCa.

## Results

### MCL1 is highly expressed in castration-resistant prostate cancer transcriptomes and associates with worse clinical outcome

**Figure 1:**
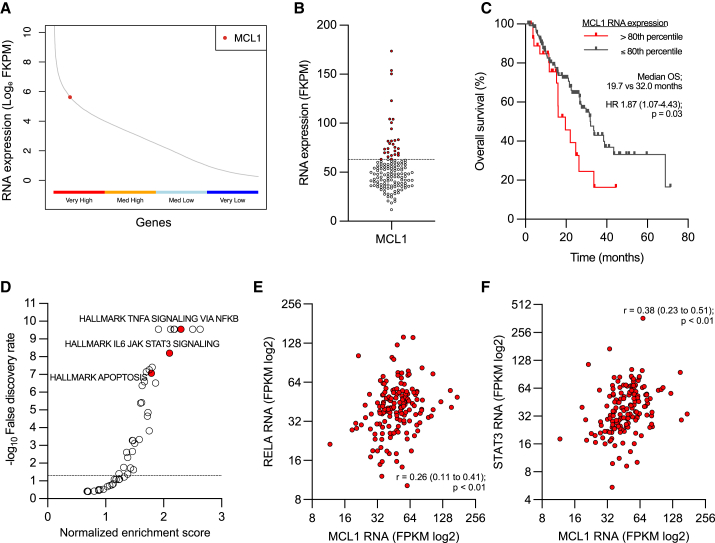
MCL1 is highly expressed in castration-resistant prostate cancer transcriptomes and associates with worse clinical outcome (A) Prostate Cancer Foundation-Stand Up To Cancer (PCF-SU2C) castration-resistant prostate cancer (CRPC) transcriptome analysis for MCL1 RNA expression compared with the 15,000 highest expressed genes divided into very high (upper 25% expressed genes), medium high (50%–75% expressed genes), medium low (25%–50% expressed genes), and very low (lower 25% expressed genes) (*n* = 159). (B) Quantification of MCL1 RNA expression in each CRPC patient transcriptome in the PCF-SU2C CRPC cohort (*n* = 141). Biopsies (red dots) with MCL1 RNA expression >80^th^ percentile (dotted line) are shown. (C) Kaplan-Meier curves for overall survival (OS) from CRPC biopsy split by > 80th percentile (red, *n* = 28) or ≤80th percentile (gray, *n* = 113) MCL1 RNA expression in the PCF-SU2C transcriptome cohort. Median OS is shown. Hazard ratio (HR) with 95% confidence intervals and *p* values for univariate cox survival model are shown. (D) Gene set enrichment analysis shows MCL1 RNA level association with hallmark pathways in the PCF-SU2C transcriptome cohort (*n* = 159). Normalized enrichment scores and false discovery rates are shown. (E and F) Association between MCL1 RNA and either RELA RNA (E) or STAT3 RNA (F) in the PCF-SU2C transcriptome cohort (*n* = 159). Spearman r- and *p* values are shown. FPKM - Fragments per kilobase of transcript per million mapped reads.

We have previously identified MCL1 and BCLXL to be highly expressed in lethal PCa, with increased MCL1 copy number associated with worse clinical outcome.^11^^,^^22^ To further investigate the biological and clinical significance of MCL1 in castration-resistant PCa (CRPC) we interrogated 159 CRPC transcriptomes from the Prostate Cancer Foundation-Stand Up To Cancer (PCF-SU2C) cohort (Figure S1).^27^ MCL1 RNA was highly expressed (top 25% expressed genes) in CRPC transcriptomes (Figure 1A). Next, we tested the association between MCL1 RNA expression and overall survival (OS) in CRPC transcriptomes (Figures 1B and 1C). Patients with higher (>80^th^ percentile) MCL1 RNA expression had shorter OS (median OS 19.7 vs. 32.0 months, hazard ratio 1.87 (95% confidence intervals 1.07–4.43), *p* = 0.03) compared to those patients with lower (≤80^th^ percentile) MCL1 RNA expression (Figures 1B and 1C). Furthermore, MCL1 RNA expression positively associated with apoptosis and multiple inflammatory signaling pathways (such as IL6 JAK STAT3, and TNFA SIGNALING VIA NFKB) that converge on transcription factors reported to regulate MCL1 levels and are implicated in PCa progression (Figure 1D; Table S9).^28^^,^^29^^,^^30^^,^^31^ Consistent with this, RELA (NFKB subunit) (r = 0.26, *p* < 0.01) and STAT3 (r = 0.38, *p* < 0.01) RNA expression positively correlated with MCL1 RNA expression in CRPC transcriptomes (Figures 1E and 1F). Taken together, these data demonstrate that MCL1 is highly expressed in CRPC, associates with worse clinical outcome, and may be regulated by pathways implicated in therapy resistance, suggesting MCL1 may be an important therapeutic target in lethal PCa.

### PCa cell line models demonstrate varying dependency on MCL1 for survival

**Figure 2:**
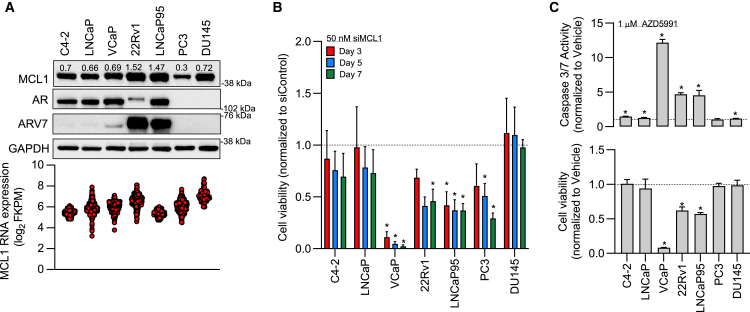
Prostate cancer cell line models demonstrate varying dependency on MCL1 for survival (A) MCL1 RNA expression was downloaded from publicly available RNA-sequencing data. Protein expression was determined across prostate cancer (PCa) cell line models. Basal MCL1, AR, AR-V7, and GAPDH protein expression was determined by western blot. Densitometry of MCL1 protein expression normalized to GAPDH protein expression is shown above each band (A, top). MCL1 RNA expression was determined across multiple publicly available RNA-sequencing experiments and presented as individual data points (A, bottom). FPKM - Fragments per kilobase of transcript per million mapped reads. (B) The impact of MCL1 (siMCL1) and non-targeting control (siControl) siRNA (50 nM) on cell viability was determined using CellTiter-Glo in PCa cell line models. Cell viability of siMCL1 compared to siControl was determined after 3, 5 and 7 days. Mean cell viability and standard deviation is shown. The experiment was performed in three biological replicates, each with three technical replicates. The unpaired Student’s *t* test was used to compare siMCL1 with siControl on each specific day for each cell line. ∗*p* value ≤ 0.05. (C) The impact of AZD5991 (1 μM) or Vehicle (DMSO 0.01%) on cell viability (CellTiter-Glo, bottom) and caspase 3/7 activity (Caspase-Glo 3/7, top) was determined in PCa cell line models. Cell viability and caspase 3/7 activity of AZD5991 compared to Vehicle is shown at 7 days and 6 h, respectively. Mean cell viability and standard deviation is shown for three biological replicates, each performed with three technical replicates. Mean caspase 3/7 activity and standard deviation is shown for one single experiment performed in sextuplets. The unpaired Student’s *t* test was used to compare AZD5991 with Vehicle. ∗*p* value ≤ 0.05.

Having demonstrated the clinical relevance of MCL1 in lethal PCa, we next determined MCL1 RNA and protein expression across PCa cell lines (Figures 2A and S2A). Expression of MCL1 RNA and protein, although at varying levels, is observed across all models studied (Figures 2A and S2A). Next, we determined the dependency of the PCa cell line models on MCL1 for proliferation and survival. Gene knockdown of MCL1 with siRNA significantly impacted the growth of PC3, 22Rv1, LNCaP95, and VCaP PCa cells (all *p* ≤ 0.05), with VCaP cells being most sensitive (Figure 2B). In addition, although only 5 of our PCa cell line models studied are contained within the publicly available DepMap database, these data orthogonally validate that MCL1 is a critical determinant for the survival of VCaP cells (Figure S2B).^32^ Furthermore, treatment of 22Rv1, LNCaP95, and VCaP PCa cells with an MCL1 specific inhibitor (1 μM AZD5991) led to increased caspase 3/7 activation and reduced cell viability (Figure 2C).^33^ Consistent with our siRNA studies and the DepMap database, VCaP PCa cells were most sensitive to chemical perturbation of MCL1. Next, given that the AR remains the predominant therapeutic target in PCa, we evaluated whether there was any association between MCL1 dependency and AR status. There was no clear association between the expression of AR/AR-V7 and the impact of *MCL1* knockdown (siRNA) or knockout (CRISPR), however, the three cell lines which responded to pharmacological inhibition expressed AR-V7 (Figures 2A–2C and S2B). Previously, we have identified that patient-derived and cell line PCa models with MCL1 copy number gain, including 22Rv1 PCa cells, are more sensitive to MCL1 inhibition.^22^ Interestingly, neither LNCaP95 nor VCaP PCa cells demonstrate MCL1 copy number gain, suggesting the presence of other mechanisms (beyond MCL1 copy number gain) that drive sensitization to MCL1 inhibition (Figure S2C).^22^ Overall, these studies demonstrate that specific PCa models are highly dependent on MCL1 for survival and interrogation of the underlying mechanisms that drive sensitivity will support the clinical development of MCL1 inhibition, biomarker stratified, treatment approaches for lethal PCa.

### siRNA targeting of UCHL3 downregulates MCL1 protein expression

**Figure 3:**
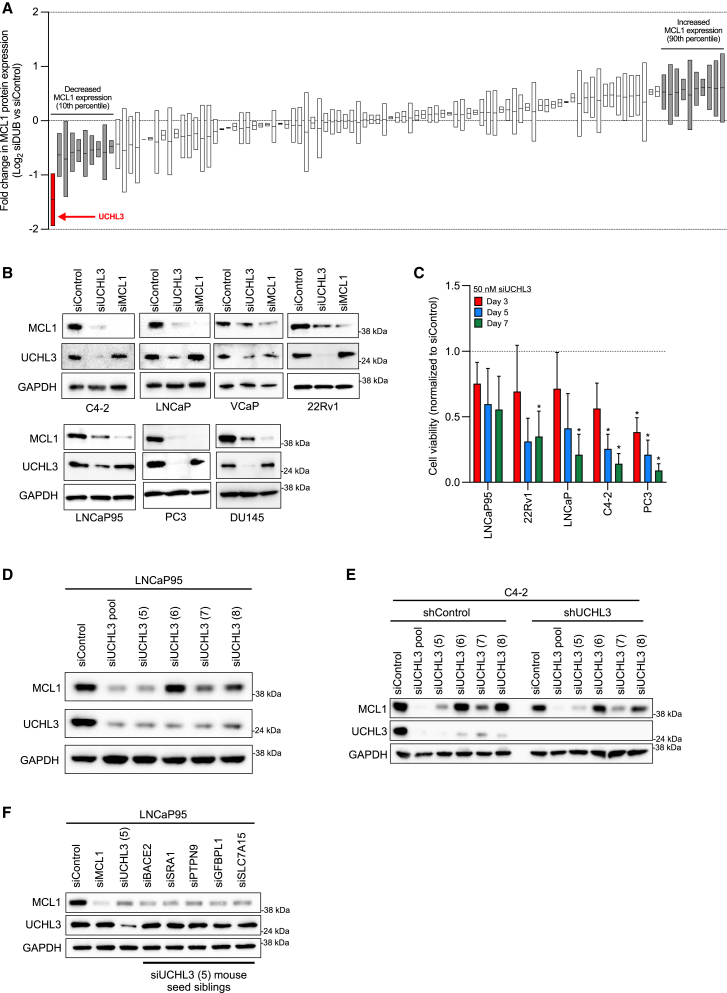
siRNA targeting of UCHL3 downregulates MCL1 protein expression, but the phenotype is driven through an off-target effect (A) LNCaP95 and 22Rv1 prostate cancer (PCa) cells were transfected with siRNA for (one of) 104 deubiquitinating enzymes (siDUBs) or non-targeting control (50 nM) for 72 h. The impact on MCL1 protein expression and GAPDH was determined by western blot. MCL1 protein expression was quantified using densitometry, normalized to GAPDH, and compared with non-targeting control (siControl). Fold change in MCL1 protein expression (Log2 siDUB vs. siControl) is shown. Mean fold change and range (effect in LNCaP95 and 22Rv1 PCa cells) is shown for a single experiment performed in both cell lines. UCHL3 is highlighted (red bar). (B) Various PCa cell line models were transfected with UCHL3 (siUCHL3), MCL1 (siMCL1) or non-targeting control (siControl) siRNA (50 nM) for 72 h and MCL1, UCHL3, and GAPDH protein expression was determined by western blot from one experiment. (C) The impact of UCHL3 (siUCHL3) and non-targeting control (siControl) siRNA (50 nM) on cell viability was determined using CellTiter-Glo in PCa cell line models. Cell viability of siUCHL3 compared to siControl was determined after 3, 5, and 7 days. Mean cell viability and standard deviation is shown for three biological replicates, each performed with three technical replicates. The unpaired Student’s *t* test was used to compare siUCHL3 with siControl on each specific day for each cell line. ∗*p* value ≤ 0.05. (D) LNCaP95 PCa cells were transfected with non-targeting control (siControl), UCHL3 pool (siUCHL3) and single siRNAs (siUCHL3 5, 6, 7, and 8) making the UCHL3 pool (50 nM) for 72 h. The effect of each condition on UCHL3, MCL1, and GAPDH protein expression was determined by western blot. Western blot from one experiment performed in biological triplicate. (E) C4-2 PCa cell shControl and shUCHL3 clones were transfected with non-targeting control (siControl), UCHL3 pool (siUCHL3), and single siRNAs (siUCHL3 5, 6, 7, and 8) making the UCHL3 pool (50 nM) for 72 h. The effect of each condition on UCHL3, MCL1, and GAPDH protein expression was determined by western blot. Western blot from one experiment performed in biological duplicate. (F) LNCaP95 PCa cells were transfected with non-targeting control (siControl), MCL1 (siMCL1), single siRNA UCHL3 5 (siUCHL3 (5)) and siUCHL3 5 mouse seed siblings (BACE2, SRA1, PTPN9, IGFBPL1, and SLC7A15) at 50 nM. The effect of each condition on UCHL3, MCL1, and GAPDH protein expression was determined by western blot at 72 h from one experiment.

Despite these promising data, clinical translation of MCL1 inhibition has been challenging due to the toxicity associated with these compounds.^23^^,^^24^ In light of this, and the importance of the ubiquitin-proteasome system in maintaining MCL1 protein expression, we investigated whether specific DUBs may be critical for MCL1 stability in PCa cells (Figure S3A).^34^^,^^35^ Interestingly, siRNA-mediated knockdown of several DUBs decreased MCL1 protein expression in 22Rv1 and LNCaP95 PCa cells (Figure 3A; Table S10). However, siRNA targeting of UCHL3 demonstrated the largest impact on MCL1 protein expression across both PCa cell lines studied (Figures 3A and S3B; Table S10). Next, to validate the results from the screen, we explored the impact of gene knockdown of UCHL3 and MCL1 with siRNA on various PCa cell lines models (Figure 3B). Consistent with our screen, siRNA targeting of UCHL3 reduced both UCHL3 and MCL1 protein expression in all PCa cell lines studied (Figure 3B). In contrast, siRNA targeting of MCL1 only reduced MCL1 protein expression (Figure 3B). Furthermore, although siRNA targeting of UCHL3 reduced MCL1 protein expression, it demonstrated a broader growth inhibitory effect on PCa cell lines, including those (LNCaP and C4-2) that were unaffected by genomic or chemical abrogation of MCL1 (Figure 3C). Taken together, these data demonstrate that siRNA targeting of UCHL3 decreases MCL1 protein expression but has broader growth inhibitory effects than genomic or chemical abrogation of MCL1 alone, suggesting the observed UCHL3-mediated phenotype in PCa cell line models is not solely dependent on MCL1 protein regulation.

### Downregulation of MCL1 protein expression by siRNA targeting of UCHL3 is driven by the off-target impact of its seed region and is independent of UCHL3

To further determine the impact of UCHL3 knockdown on MCL1 protein expression, control and UCHL3 shRNA clones were developed in LNCaP95, 22Rv1, and C4-2 PCa cells (Figure S4A). Interestingly, despite near complete knockdown of UCHL3 protein expression, in contrast to siRNA targeting of UCHL3, there was no impact on MCL1 protein expression (Figure S4A). Utilizing single siRNAs from the UCHL3 siRNA pool revealed that, despite robust knockdown of UCHL3 with all siRNA oligonucleotides, only siRNA 5 and 7 resulted in downregulation of MCL1, suggesting that the impact of the UCHL3 siRNA on MCL1 levels is off-target and independent of UCHL3 (Figures 3D and S4B). Furthermore, single siRNAs (siRNA 5 and 7) from the UCHL3 siRNA pool, and the UCHL3 siRNA pool, downregulate MCL1 protein expression in C4-2 UCHL3 shRNA clones despite there being no UCHL3 protein expression (Figure 3E). Off-target activity of siRNAs is often caused by activity of the siRNA seed region (nucleotide 2–8 of the antisense strand) which induces microRNA-like silencing of unintended targets via complementation with the 3′ untranslated region (UTR).^36^^,^^37^ A seed sibling is an siRNA that doesn’t match with any transcripts in the species of interest but whose seed sequence is a complete match with an siRNA that induces a specific phenotype. Consistent with previous reports that siRNA seed regions can regulate MCL1 protein expression, we explored the impact of 5 seed siblings that contained the seed sequence for the single UCHL3 5 siRNA (that downregulated MCL1 protein expression independent of UCHL3 protein) and specific mouse transcripts.^25^ MCL1 protein downregulation was observed with all seed siblings in LNCaP95 and C4-2 PCa cells (Figures 3F and S4C). Furthermore, UCHL3 protein expression in metastatic CRPC tissue biopsies did not associate with lower MCL1 protein expression (r = −0.16, *p* = 0.40) (Figures S4D and S4E), adding additional evidence that UCHL3 does not regulate MCL1 protein levels. Taken together, these data indicate that siRNA targeting of UCHL3 downregulates MCL1 protein through off-target actions of its seed region, which is an important consideration when studying MCL1 biology.

### Attempts to uncover the off-target driver of UCHL3 siRNA-mediated MCL1 downregulation result in the identification of another off-target siRNA effect

To identify the off-target gene regulating MCL1, which may uncover novel biology, RNA sequencing was performed in C4-2 cells after 72 h of transfection with two siRNAs that downregulate MCL1 (UCHL3 SMARTpool and oligonucleotide 5), one that does not (UCHL3 oligonucleotide 6), and a non-targeting control. Differential gene expression analysis revealed 52 genes were downregulated in both the SMARTpool and oligonucleotide 5 siRNAs compared to control when utilizing a stringent cut-off (log2 Fold Change < −2, FDR < 0.001) (Table S6; Figures S5A–S5D). Having hypothesized that one of these significantly downregulated genes may be regulating *MCL1* mRNA, an siRNA screen was undertaken aiming to identify the driver (Table S7; Figure S5E). Knockdown of RRM2 and DTL sensitized C4-2 and LNCaP95 cells to navitoclax (Figure S5E). Knockdown of DTL, but not RRM2, induced downregulation of MCL1 protein (Figure S5F). However, despite robust knockdown of DTL with each siRNA oligonucleotide, MCL1 was only downregulated with siRNA oligonucleotide 8 and the SMARTpool, once again suggesting the phenotype is driven via an off-target effect and further highlighting the importance of rigorous validation and potential confounders when utilizing siRNA to interrogate MCL1 biology (Figure S5G).

### UCHL3 expression associates with key signaling pathways in metastatic CRPC

**Figure 4:**
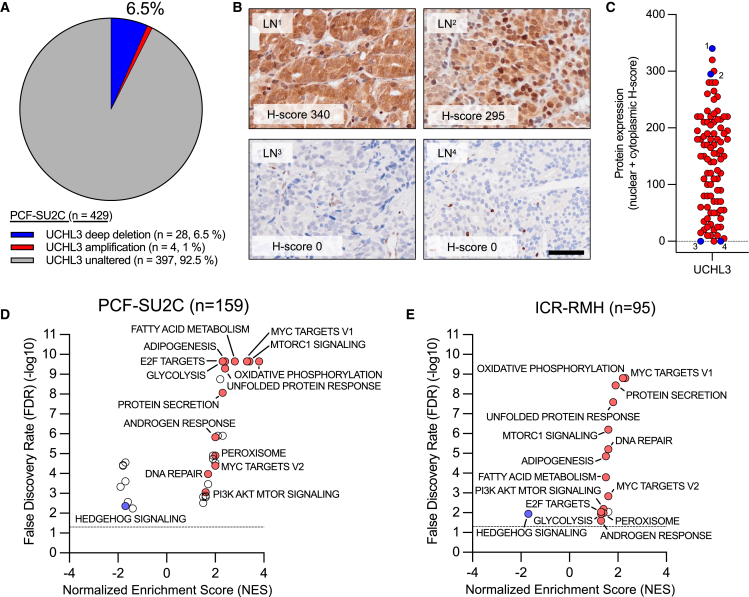
UCHL3 is variably expressed in castration-resistant metastatic prostate cancer, associating with key signaling pathways (A) Analysis of DNA-sequencing from the Prostate Cancer Foundation-Stand Up To Cancer (PCF-SU2C) castration-resistant prostate cancer (CRPC) cohort demonstrated genomic aberrations in UCHL3 (*n* = 429). (B) Representative micrographs of UCHL3 protein detection by immunohistochemistry in 4 CRPC biopsies. The H-score for total (nuclear + cytoplasmic) UCHL3 protein expression is shown. Scale bar, 50 μm. Lymph node (LN) biopsies are shown. (C) Nuclear and cytoplasmic UCHL3 protein expression (H-score) in 93 CRPC (red dots) biopsies is shown. Cases with representative micrographs are shown (blue dots). (D and E) Gene set enrichment analyses using the “hallmark molecular signatures” was performed for UCHL3 RNA expression in the (D) PCF-SU2C (*n* = 159) and (E) Institute of Cancer Research-Royal Marsden Hospital (ICR-RMH) (*n* = 95) CRPC RNA sequencing cohorts. The Spearman’s rank correlation coefficient between each gene’s expression and UCHL3 was calculated, then ranked and used for pathway analysis. Mutually enriched (red) and de-enriched (blue) pathways across both cell lines are highlighted and labeled.

Although UCHL3 does not regulate MCL1 expression, interrogation of DNA-sequencing data from the PCF-SU2C CRPC cohort identified that the UCHL3 gene is deleted in 6.5% of cases (Figure 4A). Consistent with this, following analytical validation of a UCHL3 immunohistochemistry assay, we demonstrated that 7 of 93 (7.5%) of metastatic tissue biopsies from men with CRPC had no/low (H-score ≤10) UCHL3 protein expression (Figures 4B, 4C, and S6A–6C). To investigate whether UCHL3 may have an important biological role in CRPC, we undertook gene set enrichment analysis (GSEA) for UCHL3 expression using “hallmark molecular signatures” in two independent mCRPC biopsy cohorts (PCF-SU2C and Institute of Cancer Research-Royal Marsden Hospital (ICR-RMH)). Several important pathways were enriched in both cohorts including the “unfolded protein response” which is consistent with the canonical deubiquitinating function of UCHL3 (Figures 4D and 4E; Tables S11 and S12).

### Downregulation of MCL1 protein expression sensitizes to BCLXL inhibition in PCa cell lines

**Figure 5:**
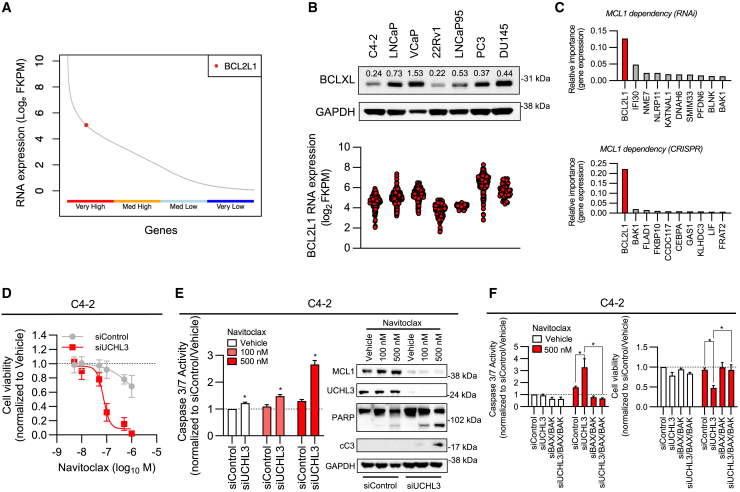
BCL2L1 is highly expressed in castration-resistant prostate cancer and MCL1 knockdown (with siUCHL3) sensitizes prostate cancer cells to BCLXL targeting (A) Prostate Cancer Foundation-Stand Up To Cancer (PCF-SU2C) castration-resistant prostate cancer (CRPC) transcriptome analysis for BCL2L1 (the gene encoding BCLXL) RNA expression compared with the 15,000 highest expressed genes divided into very high (upper 25% expressed genes), medium high (50%–75% expressed genes), medium low (25%–50% expressed genes), and very low (lower 25% expressed genes) (*n* = 159). (B) BCLXL RNA was downloaded from publicly available RNA-sequencing data. Protein expression was determined across prostate cancer cell line models. Basal BCLXL and GAPDH protein expression was determined by western blot. Densitometry of BCLXL normalized to GAPDH is shown above each band (B, top). BCLXL RNA expression was determined across multiple publicly available RNA-sequencing experiments and presented as individual data points (B, bottom). FPKM - Fragments per kilobase of transcript per million mapped reads. (C) The relative importance of specific gene expression on MCL1 dependency in cell lines (top panel—RNAi and bottom panel—CRISPR) from the DepMap database. The top 10 most important genes are shown. (D) C4-2 prostate cancer (PCa) cells were transfected with 50 nM siRNA for UCHL3 (siUCHL3) or non-targeting control (siControl). 72 h after transfection, C4-2 PCa cells were treated with various concentrations of navitoclax (BCLXL/BCL2 inhibitor) or Vehicle (DMSO 0.01%) and cell viability was determined using CellTiter-Glo after 24 h. Mean cell viability and standard deviation for siControl (gray line) and siUCHL3 (red line) compared with Vehicle is shown for three biological replicates, each performed with three technical replicates. (E) C4-2 PCa cells were transfected with 50 nM siRNA for UCHL3 (siUCHL3) or non-targeting control (siControl). 72 h after transfection, the cells were treated with two concentrations of navitoclax (100 and 500 nM) or Vehicle (DMSO 0.01%) for 6 h. The effect of each condition on caspase 3/7 activation (E, left) was determined using Caspase-Glo 3/7 and the effect of each condition on UCHL3, MCL1, PARP/cleaved PARP, cleaved caspase 3 (cC3), and GAPDH protein expression (E, right) was determined by western blot. Mean caspase 3/7 activity and standard deviation compared to siControl and Vehicle is shown for three biological replicates, each performed with three technical replicates. The unpaired Student’s *t* test was used to compare siUCHL3 with siControl for each treatment. ∗*p* value ≤ 0.05. Western blot from one experiment performed in biological triplicate. (F) C4-2 PCa cells were transfected with siRNA for UCHL3, BAX plus BAK, or UCHL3 plus BAX plus BAK siRNA, or non-targeting control (25 nM of each siRNA; total 75 nM). 72 h after transfection, the cells were treated with navitoclax (500 nM) or Vehicle (DMSO 0.01%). The impact of each condition on caspase 3/7 activation (F, left) was determined using Caspase-Glo 3/7 and the effect of each condition on cell viability (F, right) was determined by CellTiter-Glo at 6 and 24 h, respectively. Mean caspase 3/7 activity and cell viability with standard deviation compared to siControl and Vehicle is shown for three biological replicates, each performed with three technical replicates. A one-way ANOVA with post-hoc Tukey test was used to compare siUCHL3 with siControl and siUCHL3 with siUCHL3/BAX/BAK for each treatment ∗*p* value ≤ 0.05.

A major challenge of developing therapeutic strategies targeting MCL1 is the relative functional redundancy between anti-apoptotic BCL2 family protein members.^25^^,^^26^ This is further confounded by the fact that, unlike BCL2 which we have identified to be enriched in PCa tumors with evidence of lineage plasticity, MCL1 and BCL2L1 (the gene encoding BCLXL) are ubiquitously expressed in tumors from patients with lethal PCa (Figures 1A, 5A, and S1).^11^^,^^27^ Consistent with our studies on MCL1, BCLXL RNA, and protein expression is observed across all models studied (Figures 5B and S7A). In addition, the DepMap database confirms that BCL2L1 is one of the major genes that modulates MCL1 dependency in cancer cell lines (Figure 5C). Considering this finding, we next determined whether the UCHL3 siRNA, that induced downregulation of MCL1, sensitized C4-2 and LNCaP95 PCa cells to BH3 mimetics targeting BCL2/BCLXL (navitoclax and AZD4320) or BCLXL alone (A-1331852) (Figures 5D, 5E, S7B, S7C, S8A, and S8B). Interestingly, siRNA targeting of UCHL3 downregulated MCL1 and sensitized C4-2 and LNCaP95 PCa cells to navitoclax, AZD4320, and A-1331852, with induction of the intrinsic apoptosis pathway as evidenced by caspase 3/7 activation and PARP cleavage (Figures 5D, 5E, S7B, S7C, S8A, and S8B). To confirm that this phenotype was driven by the induction of the intrinsic apoptosis pathway, we next confirmed that gene knockdown of BAK and BAX rescued caspase 3/7 activation, PARP cleavage, and cell viability, in response to UCHL3 siRNA and BH3 mimetics targeting BCLXL (Figures 5F, S7D, and S8C–S8E). Overall, these data demonstrate that functional redundancy between the BCL2 family proteins remains a challenge for the development of BH3 mimetics in lethal PCa. However, the identification of novel, and existing therapeutic strategies, that target both MCL1 and BCLXL to activate the intrinsic apoptosis pathway represent an attractive approach, although the delivery of cancer specific kill remains the greatest challenge to clinical translation of these strategies.

### Pharmacological inhibition of MCL1 and BCLXL/BCL2 in patient derived and mouse models of PCa activates the intrinsic apoptosis pathway to drive cell death

**Figure 6:**
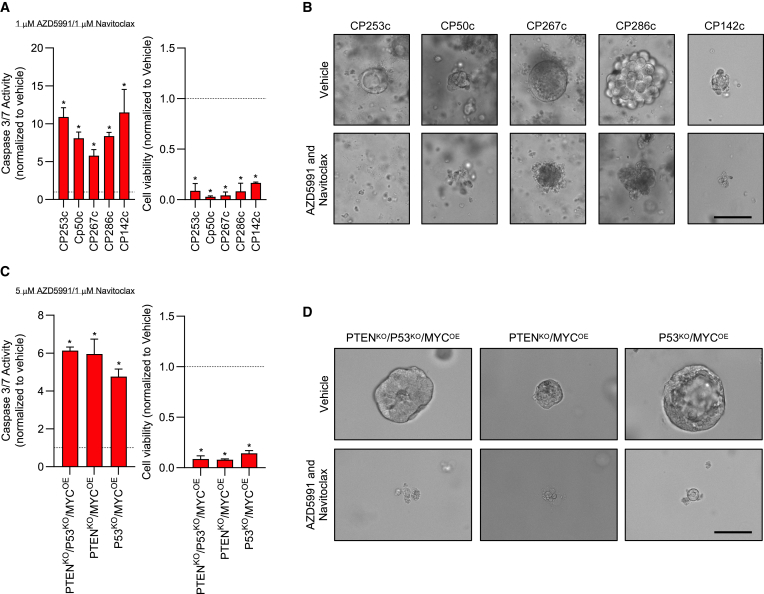
Combined MCL1 and BCLXL/BCL2 blockade drives apoptotic cell death in prostate cancer patient-derived xenograft-organoids and prostate cancer pre-clinical mouse modeling platform-organoids (A and B) Prostate cancer patient-derived xenograft-organoids (PDX-O) were treated with Vehicle or combined MCL1 (1 μM AZD5991) and BCLXL/BCL2 (1 μM navitoclax) inhibitors. The impact of each condition on caspase 3/7 activation (A, left) was determined using Caspase-Glo 3/7 and the effect of each condition on cell viability (A, right) was determined by CellTiter-Glo at 6 and 24 h respectively. Mean caspase 3/7 activity and cell viability with standard deviation compared to Vehicle is shown for three separate experiments performed in quintuplet. The unpaired Student’s *t* test was used to compare Vehicle with treatment for each model. ∗*p* value ≤ 0.05. Representative images of PDX-Os in all conditions are shown (B). Scale bar, 100 μm. (C and D) Prostate cancer pre-clinical mouse modeling platform-organoids (ProMPt-O) were treated with Vehicle or combined MCL1 (5 μM AZD5991) and BCLXL/BCL2 (1 μM navitoclax) inhibitors. The impact of each condition on caspase 3/7 activation (C, left) was determined using Caspase-Glo 3/7 and the effect of each condition on cell viability (C, right) was determined by CellTiter-Glo at 6 and 24 h, respectively. Mean caspase 3/7 activity and cell viability with standard deviation compared to Vehicle is shown for three biological replicates, each performed with three technical replicates. The unpaired Student’s *t* test was used to compare Vehicle with treatment for each model. ∗*p* value ≤ 0.05. Representative images of ProMPt-Os in all conditions are shown (D). Scale bar, 100 μm.

Having validated our previous studies that demonstrated combined MCL1 and BCLXL/BCL2 inhibition synergizes to activate the intrinsic apoptosis pathway and induce cell death in PCa cell lines, we further interrogated *in vitro* patient-derived and mouse PCa models.^11^^,^^22^ Pharmacological blockade of MCL1 and BCLXL/BCL2 in combination activated caspase 3/7 with associated cell death across multiple patient-derived PCa models and a variety of genomically distinct mouse PCa models (Figures 6A–6D). We have previously shown that PCa models with MCL1 copy number gain are more sensitive to MCL1 inhibition, but this subset is rare.^22^ These data demonstrate that targeting MCL1 and BCLXL in combination provides anti-tumor activity in a wide variety of patient-derived and mouse models of PCa that have limited therapeutic options. This supports the further development of innovative treatments targeting the anti-apoptotic BCL2 family proteins in lethal PCa.

## Discussion

Despite the development of multiple new therapies over the past two decades that have improved the clinical outcome for PCa patients, treatment resistance is inevitable, and advanced disease is invariably fatal.^2^^,^^3^^,^^4^ Therefore, the development of innovative therapeutic strategies with novel mechanisms of action for advanced PCa remains an urgent unmet medical need. One attractive strategy is to target key regulators of the intrinsic apoptosis pathway; this includes targeting the anti-apoptotic proteins (BCL2, BCLXL, and MCL1) with BH3 mimetics to breach the apoptotic threshold and drive PCa cell death.^5^^,^^6^^,^^9^^,^^10^^,^^11^^,^^12^^,^^13^^,^^14^^,^^15^^,^^16^^,^^17^^,^^18^^,^^19^^,^^20^^,^^21^ The studies herein support the clinical relevance of MCL1 in advanced PCa, demonstrating that MCL1 RNA is highly expressed in CRPC and that increased expression associates with worse clinical outcome. In addition, genomic and pharmacological abrogation of MCL1 identifies it to be a critical determinant of proliferation and/or survival in several PCa models.

Considering these findings, and to interrogate MCL1 as a therapeutic target in advanced PCa, we evaluated genomic and pharmacological abrogation of MCL1 across multiple PCa cell lines. Interestingly, a number of PCa models (including 22Rv1, LNCaP95, and VCaP cells) were sensitive to MCL1 inhibition suggesting that, despite functional redundancy between BCL2 family proteins, a subset of advanced PCas may benefit from single agent MCL1 inhibition.^25^^,^^26^ This may, in part, be due to the identification that PCa models with MCL1 copy number gain, such as 22Rv1 cells, are more sensitive to MCL1 inhibition, which is in keeping with other tumor types including multiple myeloma and lung adenocarcinoma.^22^^,^^38^^,^^39^ However, neither LNCaP95 nor VCaP harbor MCL1 copy number alternations, and it will be important to interrogate the underlying mechanisms that drive sensitivity to MCL1 inhibition in these models to support the development of biomarker stratified treatment approaches for therapies targeting MCL1 in lethal PCa.^22^ Interestingly, the studied cell lines with sensitivity to MCL1 inhibition all express AR-V7 (22Rv1, LNCaP95, and VCaP). Further studies are required to uncover any functional link. Taken together, MCL1 inhibition demonstrated anti-tumor activity in several PCa models, including those resistant to treatments that target the AR, which remains a major unmet clinical need in PCa medicine.

Studies evaluating the clinical relevance of MCL1 in PCa have demonstrated MCL1 to be upregulated in response to current PCa therapies (including androgen deprivation therapy, ARSI and taxane chemotherapy), highly expressed in CRPC, and gene copy number gains to be associated with worse clinical outcome.^6^^,^^9^^,^^11^^,^^14^^,^^22^^,^^40^^,^^41^ Consistent with this, our current study demonstrates that MCL1 is one of most abundant RNA transcripts in CRPC tissue biopsies and patients with higher MCL1 RNA levels have shorter OS. Furthermore, MCL1 RNA expression positively associated with IL6/JAK/STAT3 and TNFα signaling via NF-κB signaling pathways that converge on transcription factors, including RELA and NFKB that drive MCL1 expression and are implicated in the development of PCa treatment resistance.^28^^,^^29^^,^^30^^,^^31^ These data demonstrate the clinical relevance of MCL1 in lethal PCa and support its evaluation as a novel therapeutic target.

Despite these promising data, the clinical development of MCL1 inhibitors has been challenging due to asymptomatic troponin elevation across studies.^23^^,^^24^ In light of this, and the importance of the ubiquitin-proteasome system in maintaining MCL1 protein expression, we investigated whether DUBs may be critical for MCL1 stability in PCa cells.^34^^,^^35^ Consistent with this, genomic perturbation of a number of DUBs modulated MCL1 levels in PCa cells, with siRNA targeting UCHL3 most consistently downregulating MCL1 protein levels. However, we identified that this phenotype was independent of UCHL3 and instead driven through an off-target effect of the UCHL3 siRNA seed region. Historically, researchers have concluded that a hit should be considered a true positive if two individual siRNAs silence a specific target and drive the intended phenotype. Our data, in keeping with others, show that this assumption is not sufficient for validation.^42^ Furthermore, an additional siRNA screen, designed to identify the off-target driver of MCL1 downregulation with UCHL3 siRNA, produced another false positive result, as siRNA targeting of DTL was shown to downregulate MCL1 through an off-target, non-specific, effect. Consistent with our findings, in a previous study, the top three hits from an siRNA screen of 4000 druggable targets in combination with BCLXL/BCL2 inhibition were all found to modulate MCL1 through an off-target effect.^25^ This is an important discovery, as it highlights the liability of utilizing siRNA screens to both interrogate MCL1 biology and/or discover new therapeutic targets that function through modulating MCL1 protein expression.

Nevertheless, we identified that loss of UCHL3 occurs in a significant subset of lethal PCas. UCHL3 is a member of the ubiquitin C-terminal hydrolase (UCH) family, and although it has been reported to have contrasting biological and phenotypical roles across multiple different cancer types, it has been shown to regulate PI3K/AKT and DNA repair pathways that are known to play a role in PCa biology.^43^^,^^44^^,^^45^^,^^46^ We show that UCHL3 mRNA expression, in two independent CRPC cohorts, associates with PI3K/AKT/MTOR signaling and DNA repair, as well as other key processes in PCa including the androgen response and oxidative phosphorylation. Taken together, although UCHL3 is not functionally linked to MCL1 protein expression, it warrants further investigation which may open up novel therapeutic strategies that could be exploited in this specific molecular subset.^43^^,^^44^^,^^45^^,^^46^

In addition, we show that targeting MCL1 (directly or indirectly) synergizes with BCLXL inhibition to activate the intrinsic apoptosis. Functional redundancy between BCL2 family proteins, and high levels of expression of MCL1 and BCLXL in PCa, drives resistance to single anti-apoptotic protein inhibition.^11^^,^^25^^,^^26^^,^^27^ siRNA targeting of UCHL3 downregulated MCL1 and sensitized PCa cells to BCL2/BCLXL or BCLXL inhibition. Although these therapeutic approaches remain an attractive strategy to induce PCa cell death the ability to drive cancer specific kill from such treatment combinations remains a challenge and concerns with regard to on-target treatment related toxicity persist, namely thrombocytopenia for BCLXL and cardiotoxicity with MCL1.^6^^,^^23^^,^^24^^,^^47^ Strategies to circumvent these toxicities include utilization of ADCs, PROTACs, and nanoparticles; to preferentially target tumor cells thereby sparing exposure to vital organs.^19^^,^^48^^,^^49^^,^^50^ Another approach is to employ intermittent dosing, aiming to drive rapid apoptotic cell death but then allow sufficient time for organ recovery prior to further drug exposure.^51^ These approaches have already demonstrated anti-cancer activity with the promise of reduced toxicity.^6^^,^^19^^,^^20^^,^^52^ Despite these challenges, as we and others have shown across multiple PCa cell line models, combined targeting of MCL1 and BCLXL/BCL2 activates the intrinsic apoptosis pathway and drives cell death in patient-derived and mouse PCa models.^11^^,^^22^^,^^53^^,^^54^

Another approach to specifically kill PCa cells, while sparing normal tissues, is to identify genomic aberrations that sensitize PCa cells to single agent BH3 mimetics, as we and others have shown with respect to MCL1 copy number gains and MCL1 inhibition.^22^^,^^38^^,^^39^ In this space, a recent study has revealed that single agent BCLXL inhibition may be effective in tumors with *RB1* loss and replication stress.^55^ In addition, the demonstration that oncogenic PI3K/AKT signaling identifies cancers that are sensitive to co-targeting AKT and MCL1, suggests that rational therapeutic combinations with BH3 mimetics can drive cancer specific kill.^22^^,^^56^ Overall, the development of treatment approaches that can activate the intrinsic apoptosis pathway to deliver cancer specific cell death remains an attractive therapeutic strategy for lethal PCa.

### Limitations of the study

There are several limitations to our study. Clinical outcome data were collected retrospectively and should be interpreted with caution. Prospective studies, in multiple independent cohorts, are required to confirm MCL1 as a biomarker with prognostic clinical utility. Our study aimed to uncover a novel regulator of MCL1 protein stability, but validation experiments revealed that the impact of UCHL3 siRNA on MCL1 was via an off-target effect, independent of UCHL3. As such, there remains an unmet need to identify novel regulators of MCL1 that could be leveraged as alternative therapeutic targets or biomarkers. Although our study included a variety of cell lines and organoid models, we did not undertake *in* *vivo* studies and thus it was not possible to evaluate toxicity. However, as MCL1 inhibitors have a higher affinity for human than for murine MCL1, toxicity is difficult to study without using humanized MCL1 mice. There remain concerns regarding on-target toxicity of MCL1 and BCLXL inhibitors. The investigation of toxicity sparing strategies, including PROTACs and ADCs, may have further strengthened our study.

## Resource availability

### Lead contact

Requests for further information and resources should be directed to the lead contact, Dr Adam Sharp (adam.sharp@icr.ac.uk).

### Materials availability

This study did not generate new unique reagents.

### Data and code availability

• UCHL3 siRNA RNA-sequencing data have been deposited at the European Nucleotide Archive under accession number PRJEB101294.
• Data reported in this paper will be shared by the lead contact upon request.
• The paper does not report original code.
• Any additional information required to reanalyze the data reported in this paper is available from the lead contact upon request.

## Acknowledgments

The authors gratefully acknowledge the patients and the families of patients who contributed to this study. This work was supported by 10.13039/501100000771Prostate Cancer UK (Traveling Prize Fellowship to J.M.J.-V.; Career Acceleration Fellowship to J.T., research funding to J.S.d.B.), the 10.13039/501100000265Medical Research Council (research funding to J.S.d.B.), the 10.13039/100000892Prostate Cancer Foundation (Challenge awards to S.P.B., J.S.d.B., and A. Sharp), the Movember Foundation through the London Movember Centre of Excellence (funding to J.S.d.B.), the 10.13039/100010269Wellcome Trust (Clinical Research Career Development Fellowship to A. Sharp), 10.13039/501100000289Cancer Research UK (Clinical Research Training Fellowship to D.W. and N.W.; Center Program and Experimental Cancer Medicine Center grants to J.S.d.B., Radiation Research Network Seed Funding to A. Sharp, Convergence Center grant funding to J.S.d.B.), the 10.13039/501100000272National Institute for Health and Care Research (Academic Clinical Fellowship to D.W.), the National Institute for Health and Care Research Biomedical Research Centre (pump priming award to A. Sharp), the US Department of Defense (Early Investigator Research award to J.M.J.-V.), and 10.13039/100000002National Institutes of Health (R01CA262536 and P01CA163227 to S.P.B.).

## Author contributions

Conceptualization D.W., J.M.J.-V., and A. Sharp; methodology D.W., J.M.J.-V., A. Sharp, I.F., and J.R.; investigation D.W., J.M.J.-V., A. Sharp, I.F., J.W., B.G., D.B., L.B., A.J.N., A.P., S.D., J.T., W.Z., N.W., T.G., E.H., F.G., N.P., S.M., M.B.L., S.C., A. Swain, M.B., and W.Y.; writing-original draft D.W., J.M.J.-V., A. Sharp, and I.F.; writing-review and editing D.W., J.M.J.-V., A. Sharp, I.F., J.W., J.R., S.P.B., and J.S.d.B.; funding acquisition D.W., A. Sharp, and J.S.d.B.; resources A. Sharp, J.S.d.B., M.B., and A.S.; supervision A. Sharp, J.S.d.B., W.Y., S.P.B., and M.B.

## Declaration of interests

J.S.d.B. is an employee of the ICR, which has a commercial interest in abiraterone, PARP inhibition in DNA repair defective cancers, and PI3K/AKT pathway inhibitors (no personal income). J.S.d.B. has served on advisory boards and received fees from many companies, including Amgen, AstraZeneca, Bayer, Bioxcel Therapeutics, Daiichi, Genentech/Roche, GSK, Merck Serono, Merck Sharp & Dohme, Pfizer, and Sanofi Aventis. He is an employee of the ICR, which has received funding or other support for his research work from AstraZeneca, Astellas, Bayer, CellCentric, Daiichi, Genentech, GSK, Janssen, Merck Serono, MSD, Orion, Pfizer and Sanofi Aventis. J.S.d.B. was named as an inventor, with no financial interest, for patent 8,822,438, submitted by Janssen that covers the use of abiraterone acetate with corticosteroids. J.S.d.B. has been the CI/PI of industry-sponsored clinical trials. A. Sharp is an employee of the ICR, which has a commercial interest in abiraterone, PARP inhibition in DNA repair defective cancers, and PI3K/AKT pathway inhibitors (no personal income). A. Sharp has received travel support from Sanofi, Roche-Genentech and Nurix, and speaker honoraria from Astellas Pharma and Merck Sharp & Dohme. He has served as an advisor to DE Shaw Research, CHARM Therapeutics, Ellipses Pharma and Droia Ventures. A. Sharp has been the CI/PI of industry-sponsored clinical trials.

## STAR★Methods

### Key resources table

**Table:** 

REAGENT or RESOURCE	SOURCE	IDENTIFIER
**Antibodies**		

MCL1	Proteintech	16225-1-AP; RRID:AB_2143977
AR	Abcam	52615; RRID:AB_867653
AR-V7	Revmab	31-1109-00; RRID:AB_2716436
UCHL3	Abcam	126703; RRID:AB_10834124
DTL	Abcam	AB72264; RRID:AB_1268088
BCLXL	Cell Signaling Technology	2764; RRID:AB_2228008
BAX	Cell Signaling Technology	5023: RRID:AB_10557411
BAK	Cell Signaling Technology	12105; RRID:AB_2716685
PARP	Cell Signaling Technology	9542; RRID:AB_2160739
Cleaved caspase 3	Cell Signaling Technology	9661; RRID:AB_2341188
GAPDH	Santa Cruz Biotechnology	sc-32233; RRID:AB_627679
Vinculin	Santa Cruz Biotechnology	sc-73614; RRID:AB_1131294
αTubulin	Santa Cruz Biotechnology	sc-5286; RRID:AB_628411

**Biological samples**		

*Metastatic CRPC tissue biopsies (*The Royal Marsden Hospital/The Institute of Cancer Research)	This paper	N/A

**Chemicals, peptides, and recombinant proteins**		

DMSO/Vehicle	Fisher bioreagents, Thermo Fisher Scientific	BP231-1
AZD5991	MedChemExpress	HY-101533
Navitoclax	MedChemExpress	HY-10087
AZD4320	MedChemExpress	HY-112416
A-1331852	MedChemExpress	HY-19741

**Critical commercial assays**		

CellTiter-Glo 2D	Promega	G9241
Caspase-Glo 3/7 2D	Promega	G8093
CellTiter-Glo 3D	Promega	G9681
Caspase-Glo 3/7 3D	Promega	G8981

**Deposited data**		

CRPC patient transcriptomes	Prostate Cancer Foundation-Stand Up To Cancer (PCF-SU2C) cohort	https://pubmed.ncbi.nlm.nih.gov/31061129/
CRPC patient transcriptomes	ICR/RMH CRPC transcriptome dataset	https://pubmed.ncbi.nlm.nih.gov/35491356/
The Cancer Dependency Map project	DepMap	https://depmap.org/portal/
UCHL3 siRNA RNA-sequencing data	European Nucleotide Archive	Accession number: PRJEB101294

**Experimental models: Cell lines**		

C4-2	ATCC	CRL-3314
LNCaP	ATCC	CRL-1740
VCaP	ATCC	CRL-2876
22Rv1	ATCC	CRL-2505
LNCaP95	Dr Meeker/Dr Luo (Johns Hopkins University, Baltimore, Maryland, USA)	N/A
PC3	ATCC	CRL-1345
DU145	ATCC	HTB-81

**Experimental models: Organisms/strains**		

PCa patient-derived xenograft-organoids	The Institute of Cancer Research	https://doi.org/10.1038/s41467-025-64042-5
PCa pre-clinical mouse modeling platform-organoids	The Institute of Cancer Research	https://doi.org/10.1038/s41467-025-64042-5

**Oligonucleotides**		

Non-targeting control (smart pool)	Dharmacon	D-001810-10
MCL1 (smart pool)	Dharmacon	L-004501-00
UCHL3 (smart pool)	Dharmacon	L-006059-00
BAX (smart pool)	Dharmacon	L-003308-01
BAK1 (smart pool)	Dharmacon	L-003305-00
UCHL3 singles (individuals)	Dharmacon	L-006059-05, L-006059-06, L-006059-07, L-006059-08
UCHL3 siRNA 5 seed siblings (individuals)	Dharmacon	BACE2 (J-040326-06), SRA1 (J-045719-10), PTPN9 (J-049539-06), IGFBPL1 (J-050335-11), SLC7A15 (-053701-11)
DTL (smart pool)	Dharmacon	L-020543-00
DTL (individuals)	Dharmacon	L-020543-05, L-020543-06, L-020543-07, L-020543-08

**Software and algorithms**		

GraphPad Prism (v9)	GraphPad	https://www.graphpad.com/
Image Lab	Bio-Rad	https://www.bio-rad.com/en-uk/product/image-lab-software?ID=KRE6P5E8Z
GSEA software (v4.1.0)	Molecular Signatures Database (MSigDB)	https://www.gsea-msigdb.org/gsea/index.jsp
R v4.2.1	The R Project for Statistical Computing	https://www.r-project.org/

### Experimental model and study participant details

#### Metastatic CRPC tissue biopsies

A set of 93 metastatic tissue biopsies from 93 men with CRPC treated at the Royal Marsden Hospital was collected. Given the disease etiology, sex was not considered as a variable, and male subjects were included only. Human biological samples were sourced ethically, and their research use was in accordance with the terms of the informed consent provided. All tissue blocks were freshly sectioned and were only considered for IHC analyses if adequate material was present.

#### Cell lines and compounds

All cell lines used in this study were grown in the suppliers’ recommended media at 37 °C in 5% CO_2_ and are detailed in Table S1. Parental cell lines were tested for mycoplasma using the VenorGem One Step PCR Kit (Cambio) and STR-profiled using the cell authentication service by Eurofins Medigenomix. All compounds used are detailed in Table S2. Given the disease etiology, all cell lines were derived from male subjects.

#### *In vitro* PCa patient-derived xenograft-organoids and PCa pre-clinical mouse modeling platform-organoids

*In vitro* PCa patient-derived xenograft-organoids (PDX-O) and prostate cancer pre-clinical mouse modeling platform-organoids (ProMPt-O) were developed as previously described.^22^^,^^57^^,^^58^ Given the disease etiology, all PDXs were developed from male subjects, and male mice were utilized for the PDX-O and ProMPt-O platforms. To create PDX-Os, PDX tumors were mechanically dissociated using a sterile scalpel and subsequently passed through a 40 μm cell strainer. Red blood cells were lyzed with ammonium chloride solution, and samples were washed twice with PDS supplemented with Y-27632 before centrifugation (2000 rpm, 5 min). Single cell suspensions were obtained using 50 μm CellTrics filters and then embedded in Matrigel at a matrix-to-cell ratio of 2:1. 50 μL aliquots were placed in the center of each well. The plates were then inverted to create ‘hanging drops’ and incubated at 37 °C. Once the Matrigel domes had formed, the plates were returned to the upright position. Freshly prepared medium was added and replenished weekly. A bespoke culture medium, containing specific metabolites and growth factors, was optimized for each model. For ProMPt-Os, mouse strains including PTENLoxP; p53LoxP (JAX stock: 008462); MYCStopFL (JAX stock: 0204458); and the UBC-Cre-ERT2 (JAX stock: 007001) were utilized.^59^^,^^60^^,^^61^^,^^62^ Prostates were isolated from 3-month-old mice. Luminal and basal epithelial populations were separated by double antibody staining for CD49f and CD24. Luminal cells were embedded in 30 μL Matrigel domes in 6 well-plates, prior to supplementation with prostate organoid media.^22^ To induce Cre-ERT2-mediated excisions of floxed exons and STOP cassettes, 4-hydoxytamoxifen was added for two passages. Cre recombination was confirmed by western blot and quantitative PCR.

#### Study approvals

All clinical CRPC biopsy transcriptome analyses were performed on an already published cohort.^27^^,^^63^ All metastatic tissue biopsies from patients with CRPC used for IHC and/or PDX models (providing PDX-O) were derived from patients treated at the Royal Marsden Hospital who had provided written informed consent and were enrolled in institutional protocols approved by ethics review committee. All mouse work was carried out in accordance with The Institute of Cancer Research guidelines, including approval by The Institute of Cancer Research Animal Welfare and Ethical Review Body, and also with the UK Animals (Scientific Procedures) Act 1986 and the NCRI guidelines for the welfare and use of animals in cancer research.^64^

### Method details

#### Castration-resistant prostate cancer (CRPC) patient transcriptome analyses

CRPC patient transcriptomes from the Prostate Cancer Foundation-Stand Up To Cancer (PCF-SU2C) cohort were downloaded and re-analysed as previously described.^27^^,^^65^ In addition, the ICR/RMH CRPC transcriptome dataset was also analyzed.^63^ Briefly, paired-end transcriptome sequencing reads for the PCF-SU2C (*n* = 159) and ICR/RMH (*n* = 95) cohorts were aligned to the human reference genome (GRCh37/hg19) using Tophat2 (v2.0.7). Gene expression in Fragments Per Kilobase of transcript per Million mapped reads (FPKM) was calculated using Cufflinks. The top expressed genes (*n* = 15,000) were analyzed and gene set enrichment analysis (GSEA) was performed using the pre-ranked algorithm from GSEA software (v4.1.0) with default parameters (1000 permutations per test). The top genes were ranked from high to low using the Spearman correlation coefficient between each gene’s RNA expression and MCL1 RNA expression, and subsequently used in analysis. Results were obtained using Molecular Signatures Database hallmark gene collection and normalized enrichment scores and false discovery rates (FDR) determined.^66^ The GSEA software computes the FDR based on the Benjamini-Hochberg model.

#### Western blotting

Cell lines were lyzed with RIPA buffer (Pierce) supplemented with protease inhibitor cocktail (Roche) and PhosStop phosphatase inhibitor mix (Roche). Protein extracts (20 μg) were separated on 4–12% NuPAGE Bis-Tris gel (Invitrogen) by electrophoresis and subsequently transferred onto Immobilon-P PVDF membranes of 0.45 μm pore size (Millipore). Details of primary antibodies used are provided in Table S3. Chemiluminescence was detected on the Chemidoc Touch imaging system (Bio-Rad). Densitometry was determined using Image Lab software. The ‘adjusted volume’ of the band of interest (MCL1 or BCLXL) was normalized against the ‘adjusted volume’ of the housekeeping control (GAPDH).

#### Ribonucleic acid (RNA) sequencing (RNA-seq)

Publicly available PCa cell line RNA expression data were downloaded from the curated Combined Transcriptome dataset of PCa Cell lines (CTPC) Website and presented as log2 FPKM.^67^ To evaluate the impact of UCHL3 siRNA on the transcriptome, RNA-seq was undertaken in C4-2 cells. 250,000 cells were plated in 6 well plates and allowed to settle for 48 h, prior to transfection with non-targeting control, UCHL3 SMARTpool, UCHL3 oligonucleotide 5 and UCHL3 oligonucleotide 6 siRNAs (50 nM). After 72 h, RNA was extracted with the RNeasy Plus Mini Kit (QIAGEN), as per the manufacturer’s instructions. The experiment was performed in biological triplicate. Quantitative reverse transcription polymerase chain reaction was undertaken to confirm UCHL3 knockdown prior to RNA-seq. RNA-seq libraries were prepared using the TruSeq Stranded Total RNA Library Prep Gold Kit (Illumina) and sequenced on the Illumina NextSeq 500 platform at the Sidney Kimmel Cancer Sequencing core facility using single end 75 bp reads. FASTQ files were aligned to the human genome (GRCh37/hg19) using STAR (version 2.5.2a). Counts were generated using featureCounts (v1 1.5.0-p3) and differential expression analysis was performed using DESeq2.^68^^,^^69^

#### Immunohistochemistry (IHC)

IHC analysis for MCL1 and BCLXL protein expression on formalin fixed paraffin embedded (FFPE) PCa cell line models was carried out as previously reported.^11^ UCHL3 IHC was performed on the i6000 Automated Staining System (BioGenex) using the rabbit monoclonal anti-UCHL3 (Abcam, EPR5332) antibody. After deparaffinization and rehydration, antigen retrieval was achieved by microwaving slides in citrate buffer (pH 6.0) for 18 min (800 W), and anti-UCHL3 antibody (1:600) was incubated with tissue for 1 h at room temperature. After washes, the bound antibody was visualized using the Novolink Polymer Detection System (Leica Biosystems). Sections were counterstained with hematoxylin. Cell pellets from LNCaP95 cells treated with non-targeting control or UCHL3 siRNA, normal placenta, and rabbit IgG, were used as controls for each run.

#### IHC scoring method

MCL1 (cytoplasmic), BCLXL (cytoplasmic) and UCHL3 (nuclear and cytoplasmic) protein expression was determined by a pathologist blinded to clinical and molecular data using H-scores ([% of negative staining × 0] + [% of weak staining × 1] + [% of moderate staining × 2] + [% of strong staining × 3]), to determine the overall percentage of positivity across the entire stained samples, yielding a range from 0 to 300.^70^

#### Small interfering RNA (siRNA)

PCa cell line models were transiently transfected with siRNA as indicated. All siRNA were ON-TARGETplus pools or individuals (Dharmacon, Horizon) as listed in Table S4. The siRNAs were used with 0.2% Lipofectamine RNAiMAX transfection reagent (Thermo Fisher Scientific) as per manufacturer’s instructions and incubated with cells as indicated.

#### DepMap analyses

Publicly available data elucidating the effect of MCL1 knockout (CRISPR and RNA interference, RNAi) on PCa cell lines were downloaded from the DepMap portal (depmap.org/portal). The Cancer Dependency Map project aims to identify cancer vulnerabilities, utilizing *in vitro* CRISPR and RNAi loss-of-function screens to elucidate genetic dependencies. Details of the pipeline have been previously published.^71^ In addition, the relative importance of specific gene expression on MCL1 dependency (CRISPR and RNAi) was downloaded from the predictability tab on DepMap (Core Omics Model). These data uncover how a particular gene dependency relates to the baseline genomic status of the cell line. The top 10 most important genes were included.

#### *In vitro* cell line viability

Viability was measured for PCa cell lines using CellTiter-Glo 2D (Promega), and for patient-derived and mouse PCa models using CellTiter-Glo 3D (Promega) according to the manufacturer’s instruction. Luminescence was measured using Synergy HTX (BioTek).

#### *In vitro* caspase 3/7 assay

Caspase 3/7 activity was measured for PCa cell lines using Caspase-Glo 3/7 2D (Promega), and for patient-derived and mouse PCa models using Caspase-Glo 3/7 3D (Promega). Luminescence was measured using Synergy HTX (BioTek).

#### PDX-O and ProMPt-O

Organoids were formed in 24-well plates and subsequently reseeded into 96-well plates for drug experiments. Once established, PDX-O were treated with either Vehicle or 1 μM AZD5991 and 1 μM Navitoclax, and ProMPt-O were treated with either Vehicle or 5 uM AZD5991 and 1 μM Navitoclax. Caspase 3/7 activity (6 h) and cell viability (24 h) was determined as described above.

#### MCL1 fluorescence *in situ* hybridization (FISH)

MCL1 FISH was performed on FFPE VCaP PCa cells as previously described.^22^ Briefly, FFPE blocks were sectioned at 3–4 μm thick, and placed onto positively charged slides. Samples were deparaffinized, hydrated and boiled in pre-treatment buffer, followed by pepsin digestion. DNA was denatured at 75 °C for 10 min and subsequently hybridized at 37 °C for a minimum of 4 h. After stringency washes, the slides were mounted with Vectashield mounting medium containing DAPI (Vector Laboratories). A dual-colour FISH assay was optimized using a cocktail of two FISH probes commercially available (ZytoLight SPEC MCL1/1p12 Dual Color Probe, Bio SB), following manufacturer’s recommendations. The MCL1/centromere probe ratio was calculated in fifty intact nonoverlapping nuclei.

#### Deubiquitinating enzyme (DUB) siRNA screen

A custom DUB siRNA screen (ON-TARGETplus pools, Dharmacon, Horizon) was purchased utilizing a list of DUBs taken from the publicly available Gene Ontology Resource (Table S5).^72^^,^^73^ LNCaP95 and 22Rv1 PCa cell lines were seeded in 48 well plates at 25,000 cells per well. 24 h after plating, cells were transfected with either 50 nM DUB siRNAs or a non-targeting control as described above. After 72 h, western blot analysis was undertaken to determine the impact of individual DUBs on MCL1 protein expression as described above. Densitometric analysis was used to evaluate the MCL1 protein band quantity (Image Lab Software, Bio-Rad Laboratories). The mean fold change in MCL1 expression of both cell lines was calculated. Any DUB with a mean fold change of less than −1 was taken forward for further studies.

#### Off-target MCL1 downregulation siRNA screen

An siRNA screen was designed to target genes that were most downregulated with both SMARTpool and Oligo 5 UCHL3 siRNAs in C4-2 cells (see RNA-seq methods for details). Differential gene expression analysis revealed 52 genes were downregulated with both UCHL3 SMARTpool and oligonucleotide 5 siRNAs compared to control when utilizing a stringent cut-off (log2 Fold Change < −2, FDR <0.001) (Table S6). siRNA was available for 49 out of the 52 genes. C4-2 and LNCaP95 cells were seeded in two 96 well plates. 48 h after seeding, the cells in each plate were transfected with the 49 siRNAs (ON-TARGET plus SMARTPOOL, Dharmacon), as well as 4 negative controls (non-targeting siRNA) and two positive controls (UCHL3 SMARTpool and MCL1) (Table S7). To screen for MCL1 downregulation, after 72 h one plate was treated with the BCL2/BCLXL inhibitor Navitoclax (1 μM) and the other with vehicle (DMSO 0.01%). Given co-targeting MCL1 and BCLXL is lethal, synergy with navitoclax should be seen with any siRNA downregulating MCL1. Cell viability was evaluated with CellTiter-Glo 2D after 24 h. The fold change in cell viability for each siRNA was compared to the non-targeting control for each treatment.

#### cBioPortal for Cancer Genomics

Analyses of UCHL3 genomic aberrations was determined from DNA-sequencing of the PCF-SU2C CRPC cohort that was downloaded from cBioPortal for Cancer Genomics.^27^^,^^74^^,^^75^^,^^76^

#### Short hairpin RNA (shRNA)

Stable gene expression knockdown was undertaken with lentiviral short hairpin RNAs (shRNA) acquired from Sigma Aldrich (Table S8). C4-2, LNCaP95 and 22Rv1 cells were plated in 96 well plates at a seeding density of 5000 cells/well. After 48 h lentiviral constructs were defrosted on ice and gently spun down, then added to the well (4 μL lentiviral constructs were added to 96 μL of media). After 48 h, the viral particles were removed and replaced with fresh media. Once the cells reached a confluency of around 70%, puromycin was added for selection (4 μg/mL).

### Quantification and statistical analysis

Bioinformatic analyses are detailed in associated sections. For MCL1 RNA expression clinical analyses, patients with clinical data available (*n* = 141) were divided into two groups (≤80^th^ vs. > 80^th^ percentile). Overall survival (OS) was defined as time from CRPC biopsy to date of death or last follow up (censored). OS was estimated using the Kaplan–Meier method and log rank test for significance. Hazard ratios (HR) with 95% confidence intervals and *p*-values for univariate Cox survival models are shown. The correlation between MCL1 and both RELA and STAT3 RNA expression was determined using Spearman correlation. The unpaired Student’s t test (two groups), or ANOVA with post-hoc Tukey’s test (multiple groups), was used to determine significant differences between independent groups in all *in vitro* experiments analyzing the impact of chemical compounds or genomic manipulation on caspase 3/7 activation or cell viability. The correlation between MCL1 and UCHL3 protein expression was determined using Spearman correlation. Statistical analyses were performed with GraphPad Prism version 9 (GraphPad Software) or R version 4.2.1. All experimental replicates and statistical analyses performed are detailed in figure legends. Statistical significance was pre-specified at *p* ≤ 0.05. No adjustment for multiple testing has been made.
