# Supplementary material for: Developing therapeutic strategies to target MCL1 and BCLXL in lethal prostate cancer
Source: iScience. 2025 Nov 10;28(12):113985. doi: 10.1016/j.isci.2025.113985 (PMC12719073; doi:10.1016/j.isci.2025.113985)
Supplement: Document S1. Figures S1–S8, Tables S1–S12, Data S1, and Methods S1 [file mmc1.pdf]

iScience, Volume *28*

Supplemental information

Developing therapeutic strategies
to target MCL1 and BCLXL in lethal prostate cancer

Daniel Westaby, Juan M. Jiménez-Vacas, Ines Figueiredo, Jonathan Welti, Bora Gurel, Denisa Bogdan, Lorenzo Buroni, Antje J. Neeb, Jan Rekowski, Ana Padilha, Souvik Das, Joe Taylor, Wanting Zeng, Nick Waldron, Thomas Goldsmith, Emily Hobern, Florian Gabel, Nicole Pandell, Susana Miranda, Maryou B. Lambros, Suzanne Carreira, Amanda Swain, Wei Yuan, Steven P. Balk, Marco Bezzi, Johann S. de Bono, and Adam Sharp

Document S1. Supplementary Figures S1-8

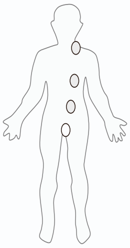

PCF-SU2C cohort
Abida at al, Proc Natl Acad Sci, 2019
n = 159 biopsies (141 for clinical analyses)
mCRPC tissue biopsies
RNA-sequencing

Supplementary figure 1: Prostate Cancer Foundation – Stand Up To Cancer castration-resistant prostate cancer transcriptome cohort. Schematic of the Prostate Cancer Foundation – Stand Up To Cancer castration-resistant prostate cancer transcriptome cohort containing 159 metastatic biopsies, of which 141 have associated clinical outcome data.

Supplementary figure 2

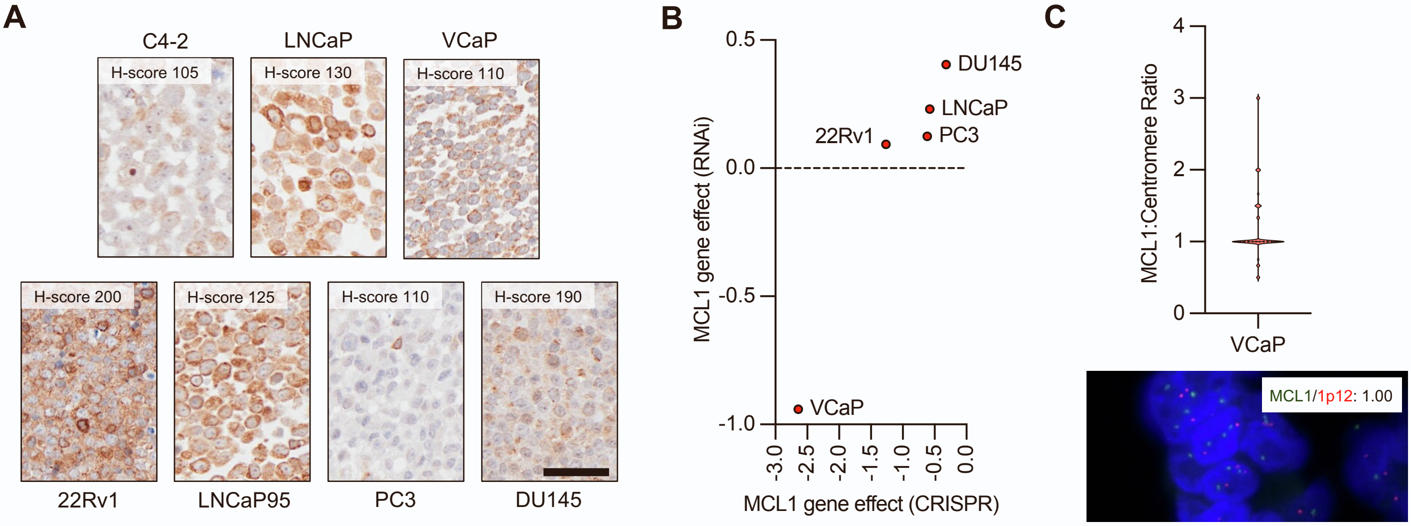

Supplementary figure 2: Prostate cancer cell line models express MCL1 protein and have varying dependency on MCL1. (A) MCL1 protein expression was determined by immunohistochemistry on formalin fixed paraffin embedded (FFPE) prostate cancer (PCa) cell line models. Representative micrographs are shown. H-score for MCL1 cytoplasmic staining is shown. Scale bar, 50 µm. **(B)** The effect of MCL1 knockout (CRISPR, x-axis) and knockdown (RNAi, y-axis) is shown in 5 prostate cancer cell lines from the DepMap database. **(C)** Representative image of MCL1 fluorescence in situ hybridisation performed on FFPE VCaP PCa cells. The median of MCL1:Centromere (1p12) ratio is shown and individual values plotted for 50 separate cells.

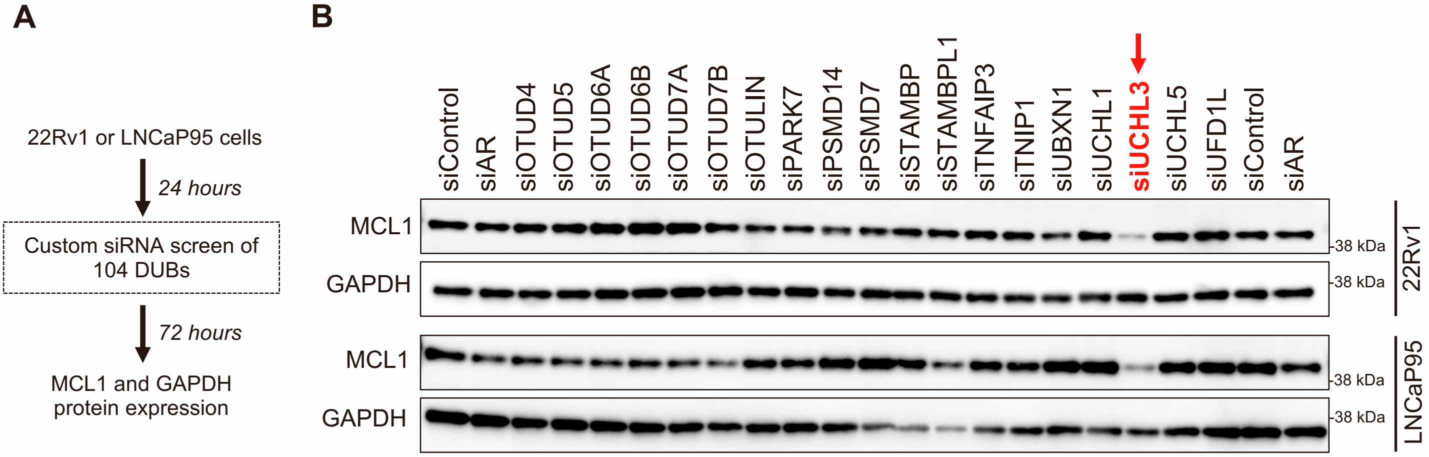

Supplementary figure 3: Deubiquitinating enzyme siRNA screen identifies specific deubiquitinating enzyme siRNAs that downregulate MCL1 protein expression. (A) Schematic of the custom siRNA screen of 104 deubiquitinating enzymes (DUBs). 22Rv1 and LNCaP95 prostate cancer (PCa) cells were plated and after 24 hours transfected with siRNA for (one of) 104 deubiquitinating enzymes or non-targeting control (50 nM) for 72 hours. The impact on MCL1 protein expression and GAPDH was then determined by western blot. **(B)** Representative western blot from the custom siRNA screen demonstrating the impact of UCHL3 (siUCHL3) siRNA (highlighted in red) on MCL1 protein expression in 22Rv1 and LNCaP95 PCa cells.

Supplementary figure 4

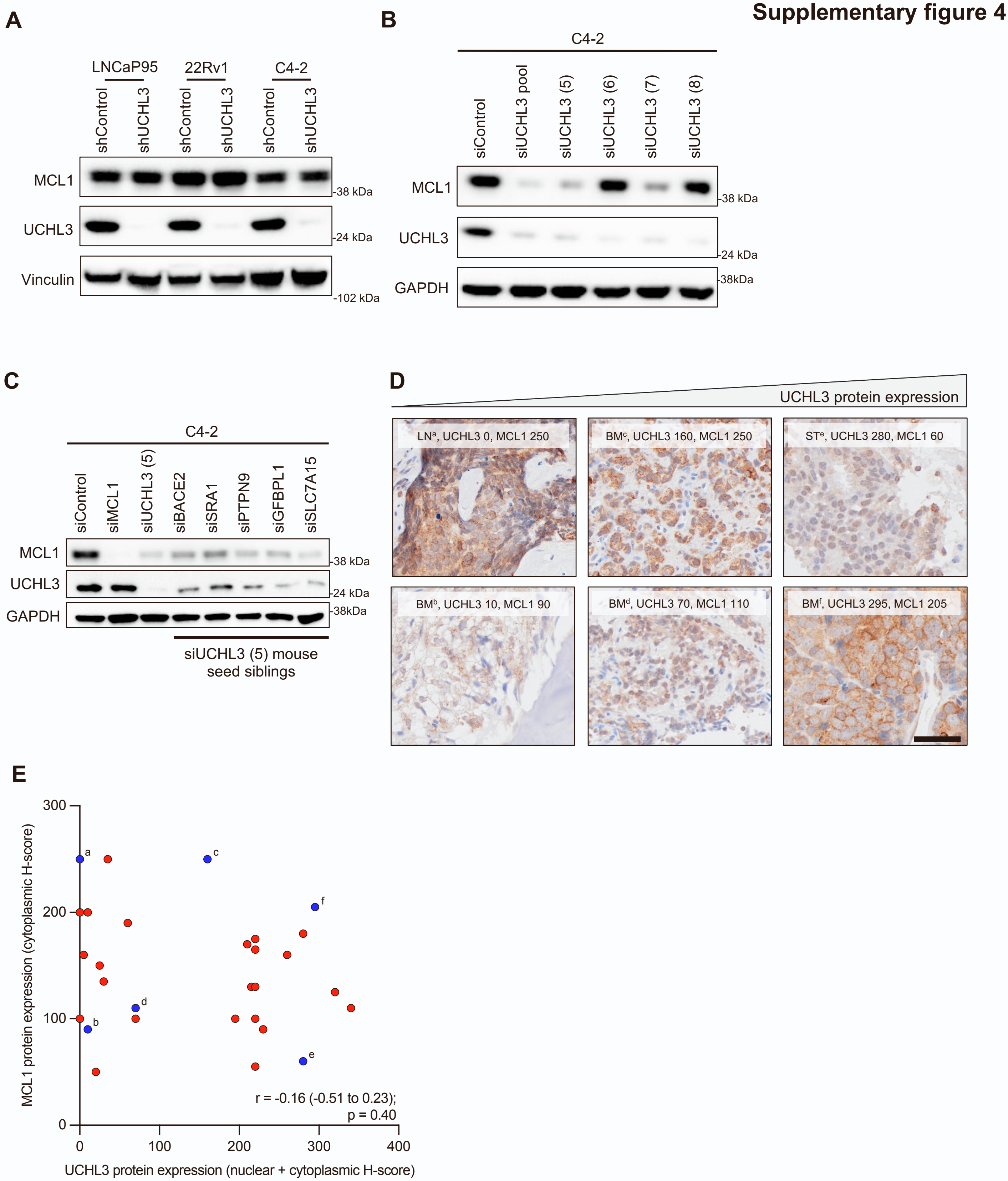

Supplementary figure 4: siRNA targeting of UCHL3 downregulates MCL1 protein expression through its off-target seed region and is independent of UCHL3. (A) shRNA Control (shControl) and UCHL3 (shUCHL3) clones were developed in LNCap95, 22Rv1 and C4-2 prostate cancer (PCa) cells. Basal levels of MCL1, UCHL3 and Vinculin protein expression for each shRNA clone was determined by western blot. **(B)** C4-2 prostate cancer (PCa) cells were transfected with non-targeting control (siControl), UCHL3 pool (siUCHL3) and single siRNAs (siUCHL3 5, 6, 7 and 8) making the UCHL3 pool (50 nM) for 72 hours. The effect of each condition on UCHL3, MCL1 and GAPDH protein expression was determined by western blot. Western blot from one experiment performed in biological triplicate. **(C)** C4-2 PCa cells were transfected with non-targeting control (siControl), MCL1 (siMCL1), single siRNA UCHL3 5 (siUCHL3 (5)) and siUCHL3 5 mouse seed siblings (BACE2, SRA1, PTPN9, IGFBPL1, SLC7A15) at 50 nM. The effect of each condition on UCHL3, MCL1 and GAPDH protein expression was determined by western blot at 72 hours. **(D)** Representative micrographs of MCL1 protein detection by IHC in 6 CRPC biopsies with various levels of UCHL3 protein expression. Scale bar, 50 µm. Lymph node (LN), bone marrow (BM) and soft tissue (ST) biopsies are shown. **(E)** Cytoplasmic MCL1 protein expression (H-score) and total (nuclear + cytoplasmic) UCHL3 protein expression (H-score) in 29 CRPC (red dots) biopsies is shown. Cases with representative micrographs are shown (blue dots).

Supplementary figure 5

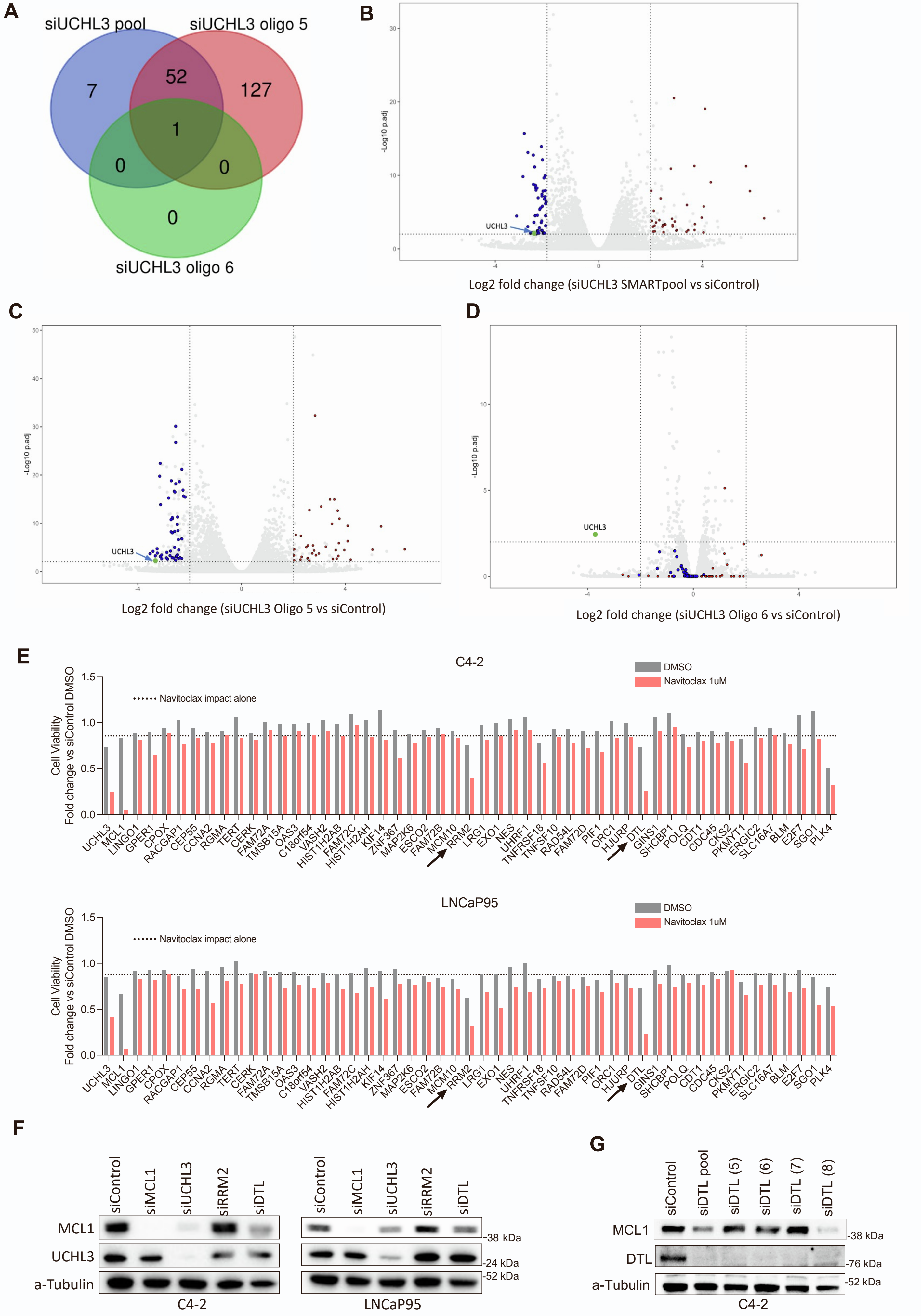

Supplementary figure 5: siRNA targeting DTL downregulates MCL1 through an off-target effect of the siRNA. **(A)** Venn diagram of mutually downregulated genes with different UCHL3 siRNAs. C4-2 cells were transfected with three different UCHL3 siRNAs (SMARTpool, oligo 5 and oligo 6) and a non-targeting control. RNA sequencing was performed after 72 hours of transfection. Venn diagram depicts mutually downregulated genes compared to the non-targeting control. The thresholds used for inclusion are log2 Fold Change < -2 and FDR <0.001. Oligo = oligonucleotide. **(B-D)** Volcano plots for UCHL3 siRNA differential gene expression analysis. C4-2 cells were transfected with three different UCHL3 siRNAs (siUCHL3 SMARTpool (B), siUCHL3 oligo 5 (C) and siUCHL3 oligo 6 (D)) and a non-targeting control (72 hours, 50nM), followed by RNA sequencing. Mutually downregulated (blue) and upregulated (red) mRNAs in SMARTpool and oligo 5 are highlighted. Dotted lines are shown at log2 fold change = -2, -log10 p adj.= 1.3. **(E)** C4-2 *(*top panel) and LNCaP95 (bottom panel) cells were transfected with siRNAs for the 49 genes identified to be downregulated with both SMARTpool and Oligo 5 UCHL3 siRNAs, UCHL3, MCL1 and a non-targeting control (50nM). After 72 hours, cells were treated with Navitoclax (1 µM) or vehicle. The impact on cell viability was assessed after 24 hours using Cell-Titre Glo. Arrows show the two genes with the most marked difference between DMSO and navitoclax (RRM2 and DTL). **(F)** C4-2 and LNCaP95 cells were transfected with non-targeting control, MCL1, UCHL3, RRM2 and DTL siRNAs. The impact on MCL1 and UCHL3 protein expression was evaluated by western blot. a-Tubulin was used as a loading control. **(G)** C4-2 cells were transfected with non-targeting control, DTL SMARTpool, DTL oligo 5, DTL oligo 6, DTL oligo 7 and DTL oligo 8 (72 hours, 50nM). The impact on DTL and MCL1 was evaluated by western blot.

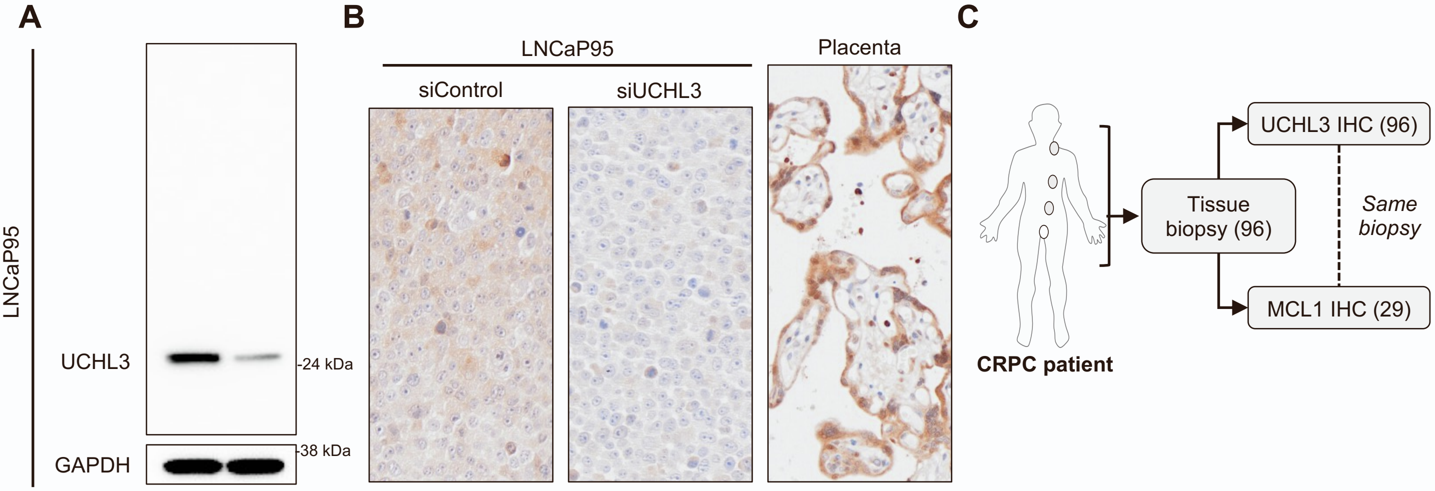

Supplementary figure 6: Analytical validation of an immunohistochemistry assay for UCHL3 protein quantification in metastatic prostate cancer tissue biopsies. (A-B) Representative western blot (A) and immunohistochemistry (IHC) (B) of UCHL3 protein detection in LNCaP95 prostate cancer (PCa) cells treated with (50 nM) non-targeting control (siControl) or UCHL3 (siUCHL3) siRNA and control tissue (placenta). Scale bar, 50 µm. **(C)** Overview of metastatic PCa tissue biopsies stained with UCHL3 and MCL1 IHC assays.

Supplementary figure 7

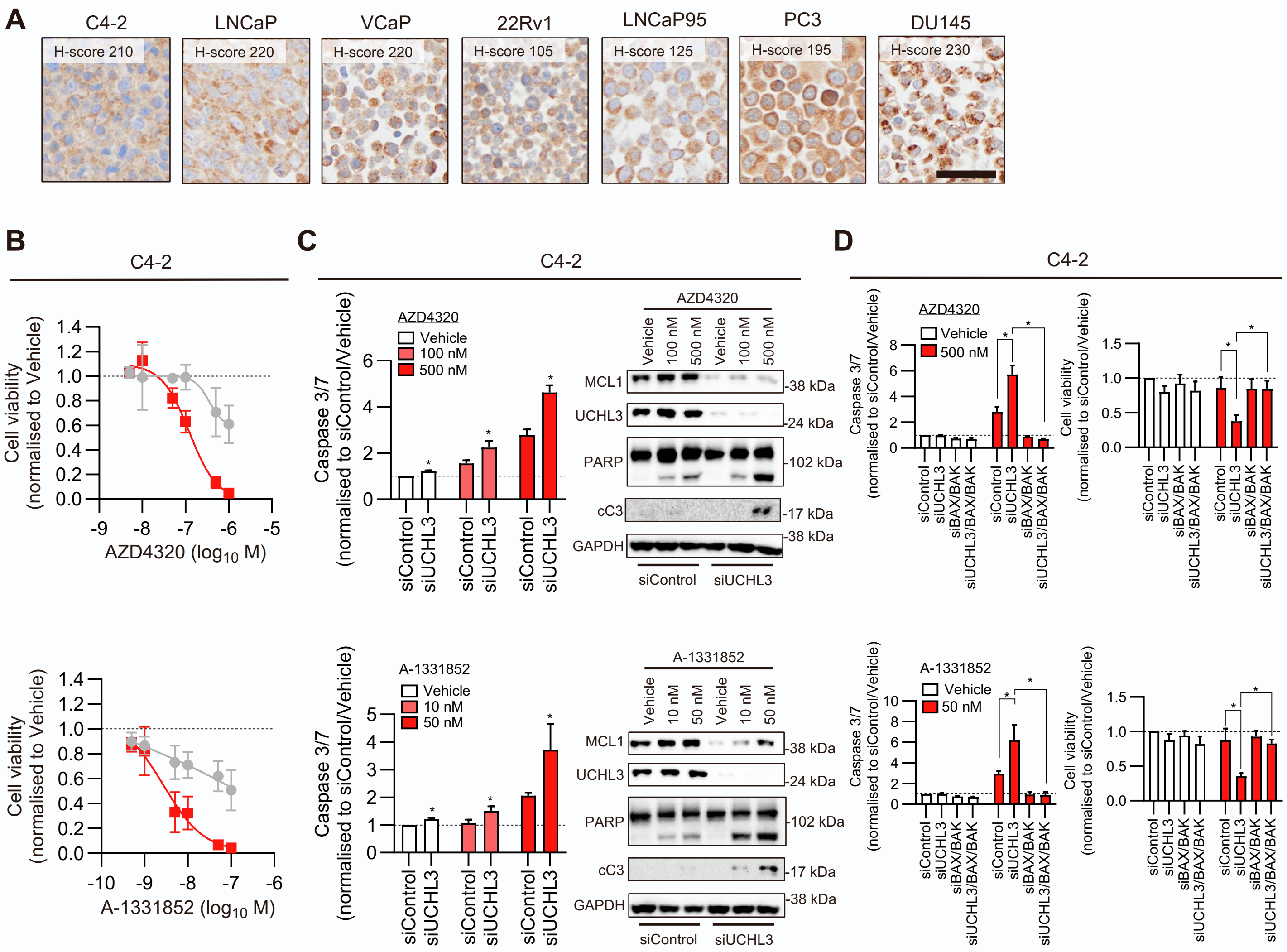

Supplementary figure 7: siRNA targeting of UCHL3 downregulates MCL1 protein expression and sensitises C4-2 prostate cancer cells to BH3 mimetics targeting BCLXL (A) BCLXL protein expression was determined by immunohistochemistry on formalin fixed paraffin embedded prostate cancer cell line models. Representative micrographs are shown. H-score for BCLXL cytoplasmic staining is shown. Scale bar, 50 µm. **(B)** C4-2 prostate cancer (PCa) cells were transfected with 50 nM siRNA for UCHL3 (siUCHL3) or non-targeting control (siControl). 72 hours after transfection, C4-2 PCa cells were treated with various concentrations of BCL2/BCLXL (AZD4320) or BCLXL (A-1331852) inhibitors or Vehicle (DMSO 0.01%) and cell viability was determined using CellTitre-Glo after 24 hours. Mean cell viability and standard deviation for siControl (grey line) and siUCHL3 (red line) compared with Vehicle is shown for three biological replicates, each performed with three technical replicates. **(C)** C4-2 prostate cancer (PCa) cells were transfected with 50 nM siRNA for UCHL3 (siUCHL3) or non-targeting control (siControl). 72 hours after transfection, the cells were treated with two concentrations of AZD4320 (100 and 500 nM), A-1331852 (10 and 50 nM) or Vehicle (DMSO 0.01%) for 6 hours. The effect of each condition on caspase 3/7 activation (C, left) was determined using Caspase-Glo and the effect of each condition on UCHL3, MCL1, PARP/cleaved PARP, cleaved caspase 3 (cC3) and GAPDH protein expression (C, right) was determined by western blot. Mean caspase 3/7 activity and standard deviation compared to siControl and Vehicle is shown for three biological replicates, each performed with three technical replicates. The unpaired student t-test was used to compare siUCHL3 with siControl for each treatment. *p-value ≤ 0.05. Western blot from one experiment performed in biological triplicate. **(D)** C4-2 PCa cells were transfected with siRNA for UCHL3, BAX plus BAK, or UCHL3 plus BAX plus BAK siRNA, or non-targeting control (25 nM of each siRNA; total 75 nM). 72 hours after transfection, the cells were treated with AZD4320 (500 nM), A-1331852 (50 nM) or Vehicle (DMSO 0.01%). The impact of each condition on caspase 3/7 activation (D, left) was determined using Caspase-Glo and the effect of each condition on cell viability (D , right) was determined by CellTitre-Glo at 6 and 24 hours respectively. Mean caspase 3/7 activity and cell viability with standard deviation compared to siControl and Vehicle is shown for three biological replicates, each performed with three technical replicates. A one-way ANOVA with post-hoc Tukey test was used to compare siUCHL3 with siControl and siUCHL3 with siUCHL3/BAX/BAK for each treatment. *p-value ≤ 0.05.

Supplementary figure 8

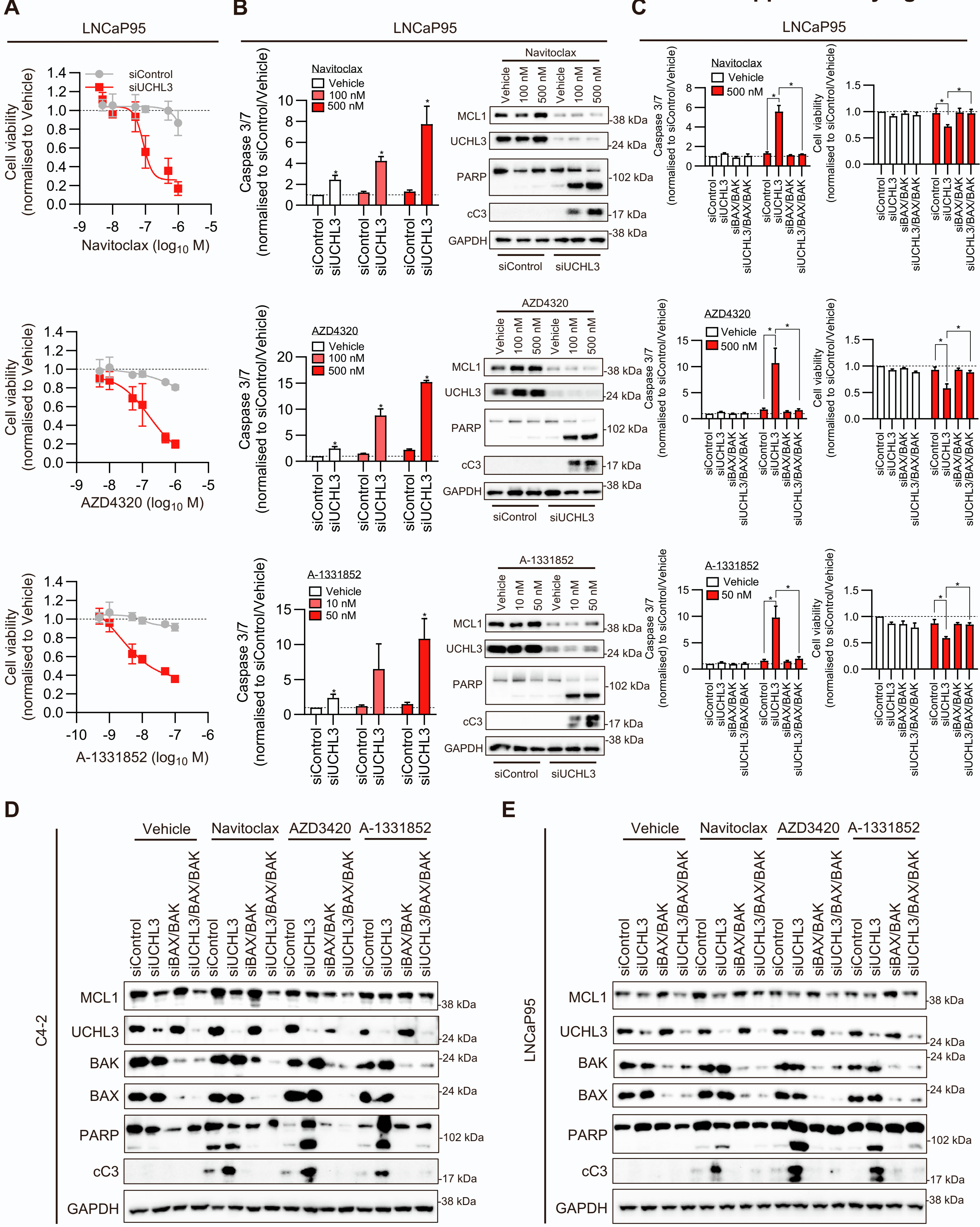

Supplementary figure 8: siRNA targeting of UCHL3 downregulates MCL1 protein expression and sensitises prostate cancer cell lines to BH3 mimetics targeting BCLXL.

(A) LNCaP95 prostate cancer (PCa) cells were transfected with 50 nM siRNA for UCHL3 (siUCHL3) or non-targeting control (siControl). 72 hours after transfection, LNCaP95 PCa cells were treated with various concentrations of BCL2/BCLXL (navitoclax and AZD4320) or BCLXL (A-1331852) inhibitors or vehicle (DMSO 0.01%) and cell viability was determined using CellTitre-Glo after 24 hours. Mean cell viability and standard deviation for siControl (grey line) and siUCHL3 (red line) compared with Vehicle is shown for three biological replicates, each performed with three technical replicates. **(B)** LNCaP95 PCa cells were transfected with 50 nM siRNA for UCHL3 (siUCHL3) or non-targeting control (siControl). 72 hours after transfection, the cells were treated with two concentrations of navitoclax (100 and 500 nM), AZD4320 (100 and 500 nM), A-1331852 (10 and 50 nM) or Vehicle (DMSO 0.01%) for 6 hours. The effect of each condition on caspase 3/7 activation (C, left) was determined using Caspase-Glo and the effect of each condition on UCHL3, MCL1, PARP/cleaved PARP, cleaved caspase 3 (cC3) and GAPDH protein expression (C, right) was determined by western blot. Mean caspase 3/7 activity and standard deviation compared to siControl and Vehicle is shown for three biological replicates, each performed with three technical replicates. The unpaired student t-test was used to compare siUCHL3 with siControl for each treatment. *p-value ≤ 0.05. Western blot from one experiment performed in biological triplicate. **(C)** LNCaP95 PCa cells were transfected with siRNA for UCHL3, BAX plus BAK, or UCHL3 plus BAX plus BAK siRNA, or non-targeting control (25 nM of each siRNA; total 75 nM). 72 hours after transfection, the cells were treated with navitoclax (500 nM), AZD4320 (500 nM), A-1331852 (50 nM) or Vehicle (DMSO 0.01%). The impact of each condition on caspase 3/7 activation (D, left) was determined using Caspase-Glo and the effect of each condition on cell viability (D, right) was determined by CellTitre-Glo at 6 and 24 hours respectively. Mean caspase 3/7 activity and cell viability with standard deviation compared to siControl and Vehicle is shown for three biological replicates, each performed with three technical replicates. A one-way ANOVA with post-hoc Tukey test was used to compare siUCHL3 with siControl and siUCHL3 with siUCHL3/BAX/BAK for each treatment. *p-value ≤ 0.05. **(D-E)** C4-2 (D) and LNCaP95 (E) were treated as described (supplementary figure 7D and 8C). The effect of each condition on MCL1, UCHL3, BAK, BAX, PARP/cleaved PARP, cleaved caspase 3 (cC3) and GAPDH protein expression was determined by western blot.

Document S2. Supplementary Tables 1-12

Supplementary tables

Cell line	Supplier	Catalogue ID	Media^	Serum (10%)
C4-2	ATCC	CRL-3314	DMEM	FBS
LNCaP	ATCC	CRL-1740	RPMI	FBS
VCaP	ATCC	CRL-2876	DMEM	FBS
22Rv1	ATCC	CRL-2505	RPMI	FBS
LNCaP95	Dr Meeker/Dr Luo*	NA	RPMI^	CSS
PC3	ATCC	CRL-1345	F12 HAM	FBS
DU145	ATCC	HTB-81	DMEM	FBS

Supplementary table 1: Cell lines

ATCC – American Type Culture Collection, RPMI – Roswell Park Memorial Institute 1640 Medium, DMEM – Dulbecco's Modified Eagle Medium, FBS – fetal bovine serum, CSS – charcoal stripped serum, * - LNCaP95 cells were a kind gift from Drs. Alan K Meeker and Jun Luo (Johns Hopkins University, Baltimore, Maryland, USA), ^ - phenol red free.

Compound	Supplier	Catalogue ID
DMSO/Vehicle	Fisher bioreagents, Thermo Fisher Scientific	BP231-1
AZD5991	MedChemExpress	HY-101533
Navitoclax	MedChemExpress	HY-10087
AZD4320	MedChemExpress	HY-112416
A-1331852	MedChemExpress	HY-19741

Supplementary table 2: Compounds

Protein target	Supplier	Catalogue ID	Dilution
MCL1	Proteintech	16225-1-AP	1:5000
AR	Abcam	52615	1:1000
AR-V7	Revmab	31-1109-00	1:1000
UCHL3	Abcam	126703	1:1000
DTL	Abcam	AB72264	1:1000
BCLXL	Cell Signaling Technology	2764	1:1000
BAX	Cell Signaling Technology	5023	1:1000
BAK	Cell Signaling Technology	12105	1:1000
PARP	Cell Signaling Technology	9542	1:1000
Cleaved caspase 3	Cell Signaling Technology	9661	1:500
GAPDH	Santa Cruz Biotechnology	sc-32233	1:2500
Vinculin	Santa Cruz Biotechnology	sc-73614	1:2500
αTubulin	Santa Cruz Biotechnology	sc-5286	1:2500

Supplementary table 3: Antibodies used for western blot analyses

Gene target	Supplier	Catalogue ID
Non-targeting control (smart pool)	Dharmacon, Horizon	D-001810-10
MCL1 (smart pool)	Dharmacon, Horizon	L-004501-00
UCHL3 (smart pool)	Dharmacon, Horizon	L-006059-00
BAX (smart pool)	Dharmacon, Horizon	L-003308-01
BAK1 (smart pool)	Dharmacon, Horizon	L-003305-00
UCHL3 singles (individuals)	Dharmacon, Horizon	L-006059-05, L-006059-06, L-006059-07, L-006059-08
UCHL3 siRNA 5 seed siblings (individuals)	Dharmacon, Horizon	BACE2 (J-040326-06), SRA1 (J-045719-10), PTPN9 (J-049539-06), IGFBPL1 (J-050335-11), SLC7A15 (-053701-11)
DTL (smart pool)	Dharmacon, Horizon	L-020543-00
DTL (individuals)	Dharmacon, Horizon	L-020543-05, L-020543-06, L-020543-07, L-020543-08

Supplementary Table 4: ON-TARGETplus siRNA

UCHL3	USP5	USP42	USP30	MYSM1
USP37	BAP1	USP13	USP26	EIF3H
OTUD7B	USP35	UFD1L	USP29	USP14
USP51	USP38	USP9X	OTUB2	USP43
USP50	COPS6	YOD1	USP31	STAMBPL1
ALG13	USP40	USP8	UBXN1	USP17L15
ATXN3	USP24	USP21	OTUD3	TNFAIP3
OTUD5	USP6	USP18	OTUD1	USP17L21
USP10	USP36	USP27X	USP17L22	USP17L10
ATXN3L	USP19	USP33	ZRANB1	USP17L11
OTUD6A	COPS5	USP17L8	USP3	USP17L13
PARK7	USP25	USP7	USP47	EIF3F
ANKZF1	USP20	USP22	JOSD2	MPND
OTUD7A	USP16	OTUB1	USP45	USP17L20
OTUD4	USP34	USP9Y	TNIP1	UCHL1
USP54	PSMD14	VCPIP1	USP17L18	USP17L2
USP1	USP11	JOSD1	UCHL5	STAMBP
OTULIN	BRCC3	USP15	USP44	USP17L17
CYLD	USP53	USP12	PSMD7	VCP
USP41	USP2	USP28	USP17L19	USP48
OTUD6B	USP17L26	USP32	USP46	

Supplementary table 5: Custom deubiquitinating enzyme ON-TARGETplus siRNA screen.

siUCHL3 siRNA	Downregulated mRNAs compared to siControl (log2 Fold Change < -2, FDR <0.01)
**SMARTpool**	NPTX1, GIPC3, ARL6IP1, NUS1, NUS1P1, HELLS, H2AC16
**Oligo 5**	QARS1, UBFD1, NETO2, CDK1, ESPL1, KIF23, KIF18B, ZNF678, TGFBR1, AURKA, NCAPH, SKA1, ASF1B, NCAPG, CBFB, ERCC6L, TCF19, CDCA8, STMN1, CENPE, ARHGAP11A, MELK, CLSPN, DDIAS, PHLDA1, CENPA, NUF2, SKA2, CDCA3, DIAPH3, CDKN3, SKA3, E2F8, CIP2A, IPMK, CXCL12, SERTAD4, RAD51AP1, RTKN2, PSRC1, KIF18A, SPC25, PCLAF, ORC6, NEIL3, KIF15, MXD3, CDC25C, CENPI, LYPLA1, KNL1, KIFC1, ANLN, CENPK, ACBD7, SMC2, FAM83D, MCM2, MKI67, DUSP19, OIP5, UBE2C, CAMKV, IQGAP3, DLGAP5, SCD5, HMGB2, NMU, NEK2, KIF20A, PBK, KIF11, NDC80, ASPM, BUB1B, AURKB, BUB1, MTFR2, GTSE1, MCM7, SPAG5, CDCA2, TACC3, FOXM1, CIT, H2BC14, HROB, CKAP2L, H2BC11, DTX4, CCNB1, CENPF, HMMR, TTK, CENPU, MYBL2, KIF2C, LMNB1, BRCA2, H2BC13, CDC20, NAV2, TPX2, PIMREG, ERFE, CCNB2, PLK1, H3C1, GCOM1, PRR11, H2BC17, CDCA5, BIRC5, ACYP1, MIR924HG, TOP2A, KIF4A, H1-5, TK1, ZNF724, KIF20B, H3C2, H2AC14, H3C13, DMBX1, DPY19L2P2, IFITM1
**SMARTpool & Oligo 5**	LINGO1, GPER1, CPOX, RACGAP1, CEP55, CCNA2, RGMA, TERT, CERK, FAM72A, TMSB15A, OAS3, C18orf54, VASH2, H2AC4, FAM72C, H4C1, H2AC12, KIF14, ZNF367, MAP2K6, ESCO2, FAM72B, MCM10, H3C15, H3C14, RRM2, LRG1, EXO1, NES, UHRF1, TNFRSF18, TNFSF10, RAD54L, FAM72D, PIF1, ORC1, HJURP, DTL, GINS1, SHCBP1, POLQ, CDT1, CDC45, CKS2, PKMYT1, ERGIC2, SLC16A7, BLM, E2F7, SGO1, PLK4
**SMARTpool & Oligo5 & Oligo6**	UCHL3

Supplementary table 6: Downregulated genes with different UCHL3 siRNAs.

C4-2 cells were transfected with three different UCHL3 siRNAs (SMARTpool, oligo 5 and oligo 6) and a non-targeting control. RNA sequencing was performed after 72 hours of transfection. The table shows downregulated genes compared to the non-targeting control. The thresholds used for inclusion are log2 Fold Change < -2 and FDR <0.001. Oligo - oligonucleotide.

LINGO1	TMSB15A	ESCO2	RAD54L	CDC45
GPER1	OAS3	FAM72B	FAM72D	CKS2
CPOX	C18orf54	MCM10	PIF1	PKMYT1
RACGAP1	VASH2	RRM2	ORC1	ERGIC2
CEP55	HIST1H2AB	LRG1	HJURP	SLC16A7
CCNA2	FAM72C	EXO1	DTL	BLM
RGMA	HIST1H2AH	NES	GINS1	E2F7
TERT	KIF14	UHRF1	SHCBP1	SGO1
CERK	ZNF367	TNFRSF18	POLQ	PLK4
FAM72A	MAP2K6	TNFSF10	CDT1	

Supplementary Table 7: Off-target MCL1 downregulation siRNA screen gene set.

Gene Target	Catalogue ID	Vector Backbone	Selection	Supplier
Control	SHC0002V	pLKO.1	Puromycin	Sigma Aldrich
UCHL3	TRCN0000320682	pLKO.1	Puromycin	Sigma Aldrich

Supplementary Table 8: MISSION Lentiviral shRNA used for stable UCHL3 gene expression knockdown

Gene set enrichment analysis for MCL1 mRNA expression (PCF-SU2C RNA-sequencing cohort)		
**Pathway**	**NES**	**FDR**
HALLMARK INTERFERON ALPHA RESPONSE	2.63	2.86e-10
HALLMARK INTERFERON GAMMA RESPONSE	2.50	2.86e-10
HALLMARK TNFA SIGNALING VIA NFKB	2.30	2.86e-10
HALLMARK INFLAMMATORY RESPONSE	2.19	2.86e-10
HALLMARK ALLOGRAFT REJECTION	2.18	2.86e-10
HALLMARK COMPLEMENT	2.12	2.86e-10
HALLMARK IL6 JAK STAT3 SIGNALING	2.10	6.30e-09
HALLMARK KRAS SIGNALING UP	1.91	2.86e-10
HALLMARK PROTEIN SECRETION	1.86	3.07e-07
HALLMARK UV RESPONSE DN	1.80	3.97e-08
HALLMARK APOPTOSIS	1.79	8.67e-08
HALLMARK TGF BETA SIGNALING	1.75	1.51e-04
HALLMARK IL2 STAT5 SIGNALING	1.75	5.48e-08
HALLMARK PI3K AKT MTOR SIGNALING	1.73	1.42e-05
HALLMARK COAGULATION	1.72	2.23e-05
HALLMARK MITOTIC SPINDLE	1.70	7.07e-08
HALLMARK MTORC1 SIGNALING	1.67	2.61e-07
HALLMARK G2M CHECKPOINT	1.63	4.26e-07
HALLMARK EPITHELIAL MESENCHYMAL TRANSITION	1.62	3.85e-06
HALLMARK HYPOXIA	1.60	4.11e-06
HALLMARK ANDROGEN RESPONSE	1.58	4.69e-04
HALLMARK UNFOLDED PROTEIN RESPONSE	1.50	7.69e-04
HALLMARK APICAL SURFACE	1.48	2.36e-02
HALLMARK APICAL JUNCTION	1.47	5.38e-04
HALLMARK P53 PATHWAY	1.46	5.29e-04
HALLMARK UV RESPONSE UP	1.46	2.22e-03
HALLMARK NOTCH SIGNALING	1.43	1.85e-02
HALLMARK ANGIOGENESIS	1.38	4.11e-02
HALLMARK HEME METABOLISM	1.38	4.76e-03
HALLMARK E2F TARGETS	1.36	2.49e-03
HALLMARK HEDGEHOG SIGNALING	1.32	6.05e-02
HALLMARK ESTROGEN RESPONSE EARLY	1.31	1.75e-02
HALLMARK CHOLESTEROL HOMEOSTASIS	1.24	8.01e-02
HALLMARK REACTIVE OXYGEN SPECIES PATHWAY	1.23	8.36e-02
HALLMARK GLYCOLYSIS	1.23	3.93e-02
HALLMARK MYOGENESIS	1.18	8.36e-02
HALLMARK ESTROGEN RESPONSE LATE	1.15	8.37e-02
HALLMARK SPERMATOGENESIS	1.13	1.46e-01
HALLMARK BILE ACID METABOLISM	1.11	1.64e-01
HALLMARK PEROXISOME	1.07	1.80e-01
HALLMARK MYC TARGETS V1	1.03	2.09e-01
HALLMARK ADIPOGENESIS	0.96	2.95e-01
HALLMARK KRAS SIGNALING DN	0.90	3.16e-01
HALLMARK WNT BETA CATENIN SIGNALING	0.88	3.16e-01
HALLMARK XENOBIOTIC METABOLISM	0.88	3.47e-01
HALLMARK FATTY ACID METABOLISM	0.80	3.78e-01

HALLMARK DNA REPAIR	0.70	3.94e-01
HALLMARK PANCREAS BETA CELLS	0.69	3.78e-01
HALLMARK MYC TARGETS V2	0.68	3.90e-01

Supplementary table 9: Cellular pathways enriched with MCL1 RNA expression in CRPC patient transcriptomes.

NES – normalized enrichment score, FDR – false discovery rate.

DUB	Fold change (log2) in MCL1 protein expression: 22Rv1	Fold change (log2) in MCL1 protein expression: LNCaP95	Mean
UCHL3	-0.97	-1.94	-1.46
USP37	-0.01	-1.40	-0.71
OTUD7B	-1.03	-0.23	-0.63
USP51	-0.17	-1.03	-0.60
USP50	-0.07	-1.09	-0.58
ALG13	-0.22	-0.94	-0.58
ATXN3	-0.34	-0.76	-0.55
OTUD5	-1.32	0.23	-0.55
USP10	-0.81	-0.27	-0.54
ATXN3L	-0.38	-0.67	-0.53
OTUD6A	-1.15	0.16	-0.50
PARK7	-0.36	-0.60	-0.48
ANKZF1	0.08	-0.97	-0.45
OTUD7A	-0.89	0.03	-0.43
OTUD4	-0.97	0.19	-0.39
USP54	-1.00	0.24	-0.38
USP1	-0.34	-0.34	-0.34
OTULIN	-0.36	-0.30	-0.33
CYLD	-0.94	0.29	-0.33
USP41	-0.69	0.11	-0.29
OTUD6B	-0.86	0.29	-0.29
USP5	0.14	-0.69	-0.28
BAP1	-0.17	-0.36	-0.27
USP35	0.06	-0.54	-0.24
USP38	-0.32	-0.15	-0.24
COPS6	-0.94	0.52	-0.21
USP40	-0.42	0.03	-0.20
USP24	-0.14	-0.17	-0.16
USP6	0.15	-0.43	-0.14
USP36	0.41	-0.69	-0.14
USP19	-0.14	-0.12	-0.13
COPS5	-0.42	0.16	-0.13
USP25	-0.18	-0.04	-0.11
USP20	0.19	-0.40	-0.11
USP16	-0.17	-0.01	-0.09
USP34	0.23	-0.40	-0.09
PSMD14	0.25	-0.42	-0.09
USP11	-0.04	-0.12	-0.08
BRCC3	-0.04	-0.10	-0.07
USP53	0.01	-0.14	-0.07
USP2	-0.07	-0.04	-0.06
USP17L26	0.07	-0.15	-0.04
USP42	-0.20	0.14	-0.03
USP13	0.36	-0.42	-0.03
UFD1L	-0.25	0.21	-0.02

USP9X	0.19	-0.22	-0.02
YOD1	0.12	-0.14	-0.01
USP8	0.20	-0.22	-0.01
USP21	0.15	-0.12	0.02
USP18	0.32	-0.27	0.03
USP27X	0.11	-0.06	0.03
USP33	0.11	-0.06	0.03
USP17L8	0.33	-0.23	0.05
USP7	0.08	0.03	0.06
USP22	-0.03	0.15	0.06
OTUB1	0.18	-0.03	0.08
USP9Y	0.19	0.01	0.10
VCPIP1	0.25	-0.03	0.11
JOSD1	-0.18	0.40	0.11
USP15	0.19	0.04	0.12
USP12	0.36	-0.12	0.12
USP28	-0.03	0.28	0.13
USP32	0.20	0.06	0.13
USP30	0.10	0.16	0.13
USP26	0.23	0.04	0.14
USP29	-0.27	0.56	0.15
OTUB2	0.55	-0.25	0.15
USP31	0.20	0.11	0.16
UBXN1	0.40	-0.09	0.16
OTUD3	0.71	-0.38	0.17
OTUD1	0.60	-0.25	0.18
USP17L22	0.40	0.00	0.20
ZRANB1	0.15	0.26	0.21
USP3	-0.04	0.48	0.22
USP47	0.18	0.32	0.25
JOSD2	0.16	0.40	0.28
USP45	0.18	0.39	0.29
TNIP1	0.23	0.36	0.30
USP17L18	0.93	-0.29	0.32
UCHL5	0.26	0.40	0.33
USP44	0.36	0.33	0.35
PSMD7	1.07	-0.38	0.35
USP17L19	0.96	-0.22	0.37
USP46	0.14	0.70	0.42
MYSM1	0.50	0.34	0.42
EIF3H	0.00	0.87	0.44
USP14	0.81	0.06	0.44
USP43	0.07	0.84	0.46
STAMBPL1	0.81	0.10	0.46
USP17L15	0.84	0.10	0.47
TNFAIP3	0.68	0.26	0.47
USP17L21	0.70	0.25	0.48
USP17L10	1.12	-0.17	0.48

USP17L11	1.04	-0.07	0.49
USP17L13	0.96	0.08	0.52
EIF3F	0.58	0.48	0.53
MPND	0.21	0.85	0.53
USP17L20	0.64	0.44	0.54
UCHL1	0.77	0.38	0.58
USP17L2	0.90	0.28	0.59
STAMBP	1.11	0.08	0.60
USP17L17	1.24	-0.03	0.61
VCP	0.46	0.77	0.62
USP48	0.20	1.03	0.62

Supplementary table 10: Impact of deubiquitinating enzyme knockdown on MCL1 protein expression in 22Rv1 and LNCaP95 prostate cancer cells.

DUB – Deubiquitinating enzyme

Gene set enrichment analysis for UCHL3 mRNA expression (PCF-SU2C RNA-sequencing cohort)		
**Pathway**	**NES**	**FDR**
OXIDATIVE PHOSPHORYLATION	3.8	2.26E-10
MTORC1 SIGNALING	3.4	2.26E-10
MYC TARGETS V1	3.3	2.26E-10
FATTY ACID METABOLISM	2.8	2.26E-10
UNFOLDED PROTEIN RESPONSE	2.4	5.11E-10
ADIPOGENESIS	2.4	2.26E-10
PROTEIN SECRETION	2.3	8.52E-09
GLYCOLYSIS	2.3	2.26E-10
E2F TARGETS	2.3	2.26E-10
REACTIVE OXYGEN SPECIES PATHWAY	2.3	0.00000126
INTERFERON GAMMA RESPONSE	2.2	1.76E-09
INTERFERON ALPHA RESPONSE	2.1	0.00000126
ANDROGEN RESPONSE	2	0.00000149
CHOLESTEROL HOMEOSTASIS	2	0.000029
PEROXISOME	2	0.0000124
MYC TARGETS V2	2	0.00004
XENOBIOTIC METABOLISM	1.9	0.0000126
UV RESPONSE UP	1.9	0.0000181
DNA REPAIR	1.7	0.000106
APOPTOSIS	1.7	0.00034
PI3K AKT MTOR SIGNALING	1.6	0.000887
BILE ACID METABOLISM	1.6	0.00153
ALLOGRAFT REJECTION	1.6	0.00131
ESTROGEN RESPONSE LATE	1.6	0.00131
G2M CHECKPOINT	1.5	0.0015
HEME METABOLISM	1.5	0.00308
HYPOXIA	1.5	0.00308
MYOGENESIS	-1.4	0.00574
KRAS SIGNALING DN	-1.6	0.00271
EPITHELIAL MESENCHYMAL TRANSITION	-1.7	0.000247
MITOTIC SPINDLE	-1.7	0.0000276
HEDGEHOG SIGNALING	-1.7	0.00436
APICAL JUNCTION	-1.8	0.000038
UV RESPONSE DN	-1.8	0.00004
WNT BETA CATENIN SIGNALING	-1.9	0.000464

Supplementary table 11: Gene set enrichment analyses using the ‘hallmark molecular signatures’ for UCHL3 mRNA expression in PCF-SU2C CRPC RNA sequencing cohort. NES - normalised enrichment score, FDR- false discovery rate.

Gene set enrichment analysis for UCHL3 mRNA expression (ICR/RMH RNA-sequencing cohort)		
**Pathway**	**NES**	**FDR**
MYC TARGETS V1	2.3	1.58E-09
OXIDATIVE PHOSPHORYLATION	2.2	1.58E-09
PROTEIN SECRETION	1.9	3.67E-09
UNFOLDED PROTEIN RESPONSE	1.8	2.61E-08
PANCREAS BETA CELLS	1.6	0.00901
DNA REPAIR	1.6	0.00000617
MYC TARGETS V2	1.6	0.00146
MTORC1 SIGNALING	1.6	6.39E-07
ADIPOGENESIS	1.5	0.000014
FATTY ACID METABOLISM	1.5	0.000163
PI3K AKT MTOR SIGNALING	1.4	0.00648
PEROXISOME	1.4	0.0104
ANDROGEN RESPONSE	1.3	0.0252
GLYCOLYSIS	1.3	0.0113
E2F TARGETS	1.3	0.00901
HEDGEHOG SIGNALING	-1.7	0.0113

Supplementary table 12: Gene set enrichment analyses using the 'hallmark molecular signatures' for UCHL3 mRNA expression in ICR-RMH CRPC RNA sequencing cohort. NES - normalised enrichment score, FDR - false discovery rate.

Data S1/Methods S1. Raw western blot images

Fig 2A

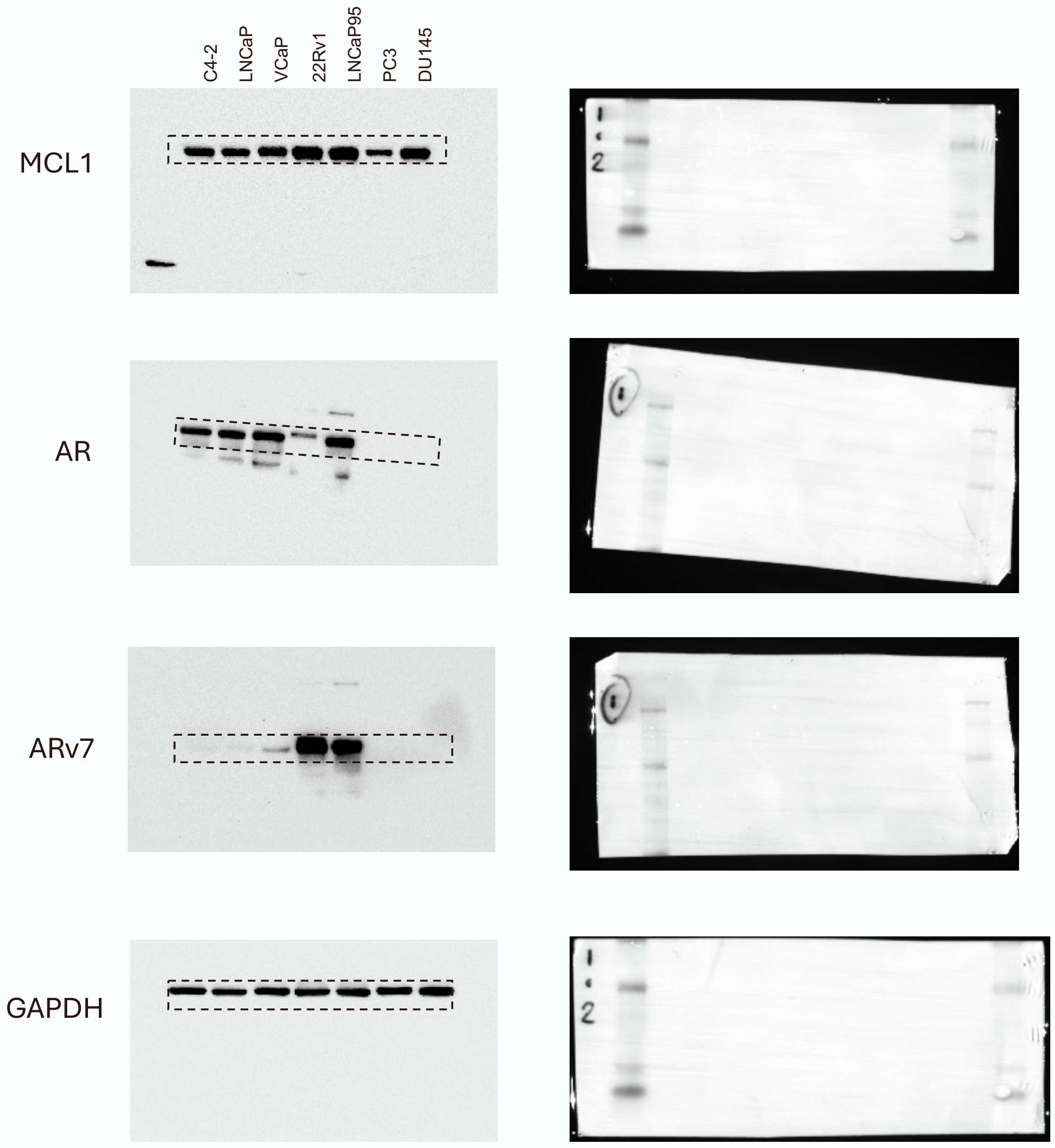

Fig 3B – LNCaP95 & 22Rv1

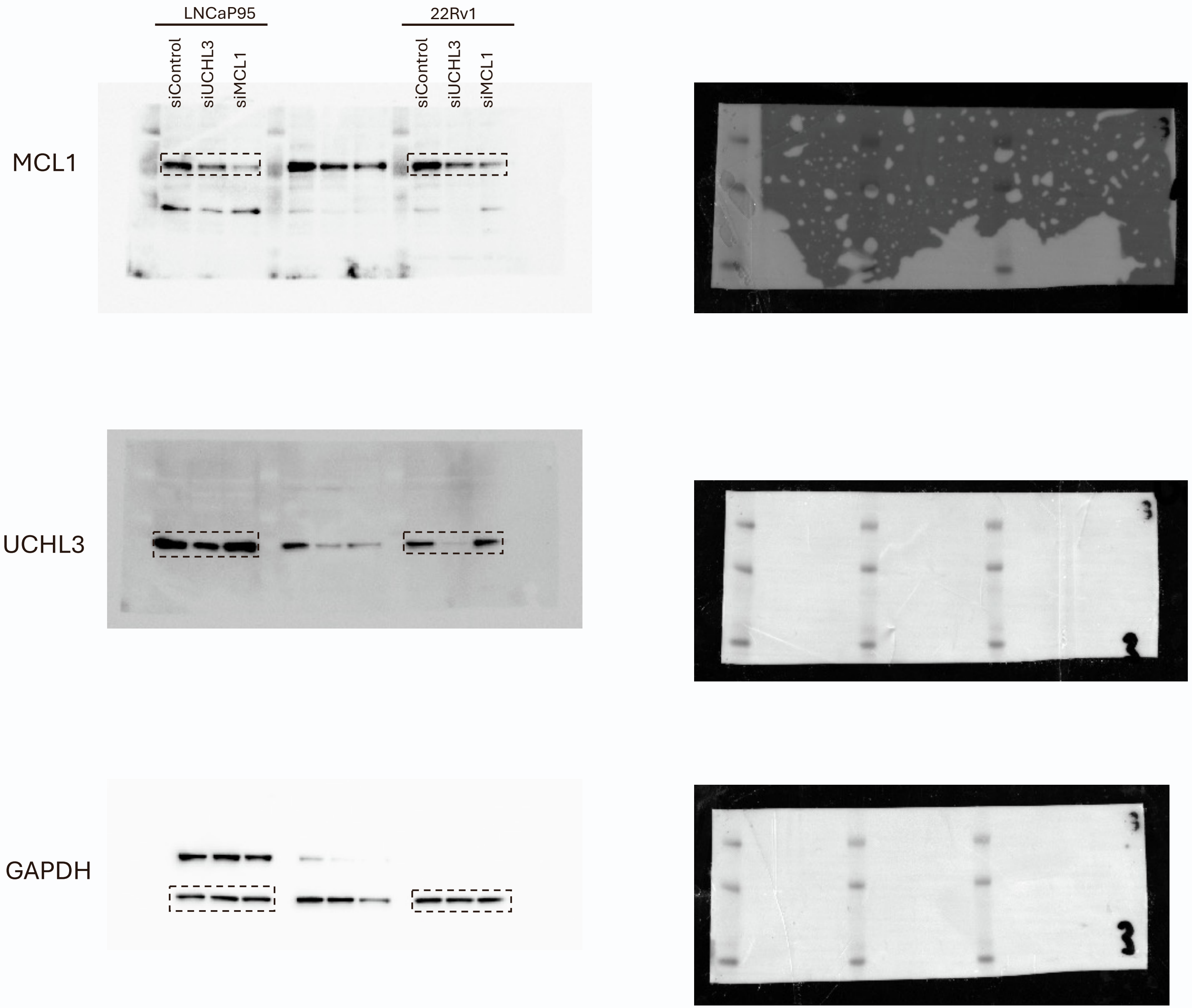

Fig 3B – PC3

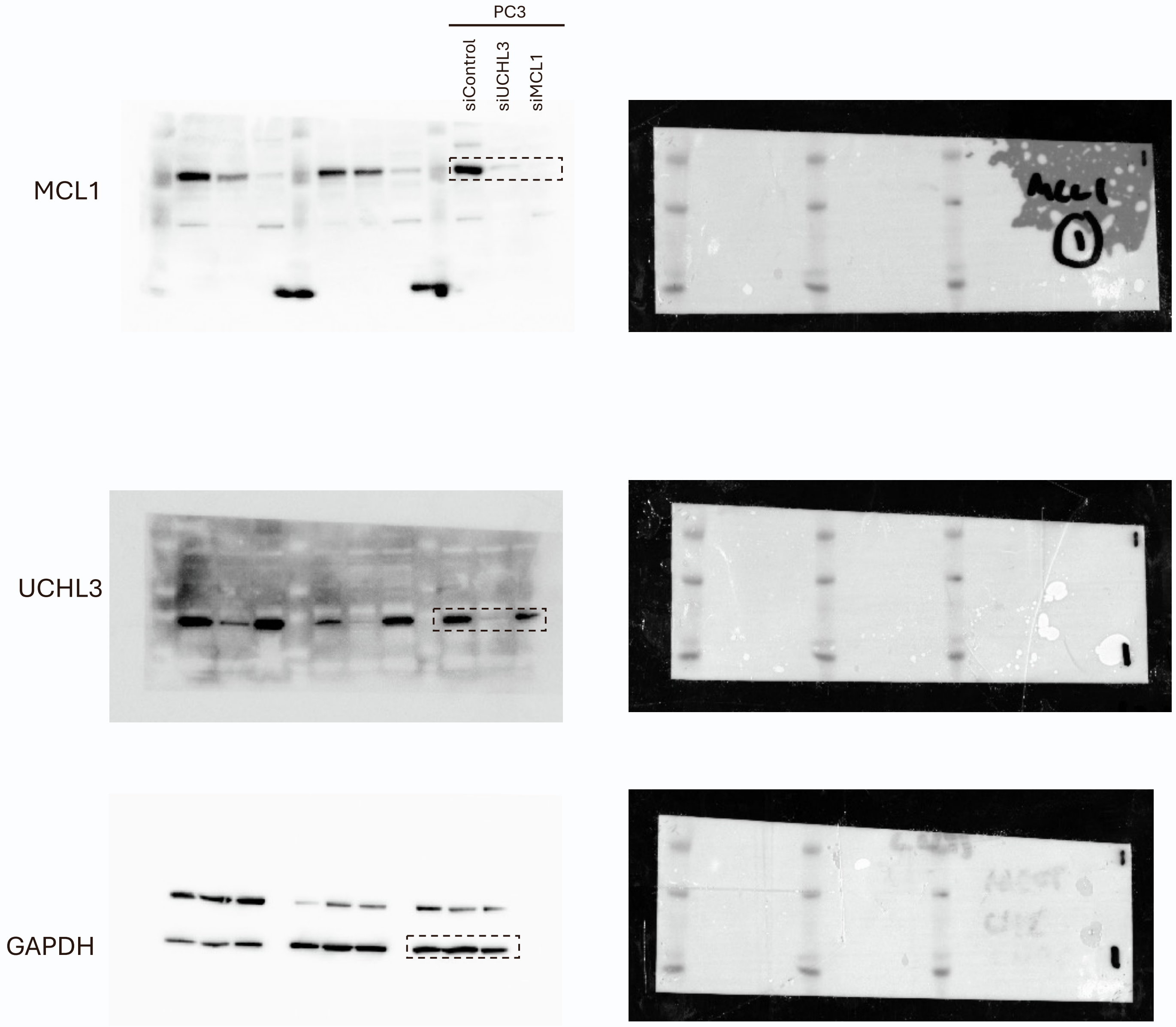

Fig 3B – LNCaP & C4-2

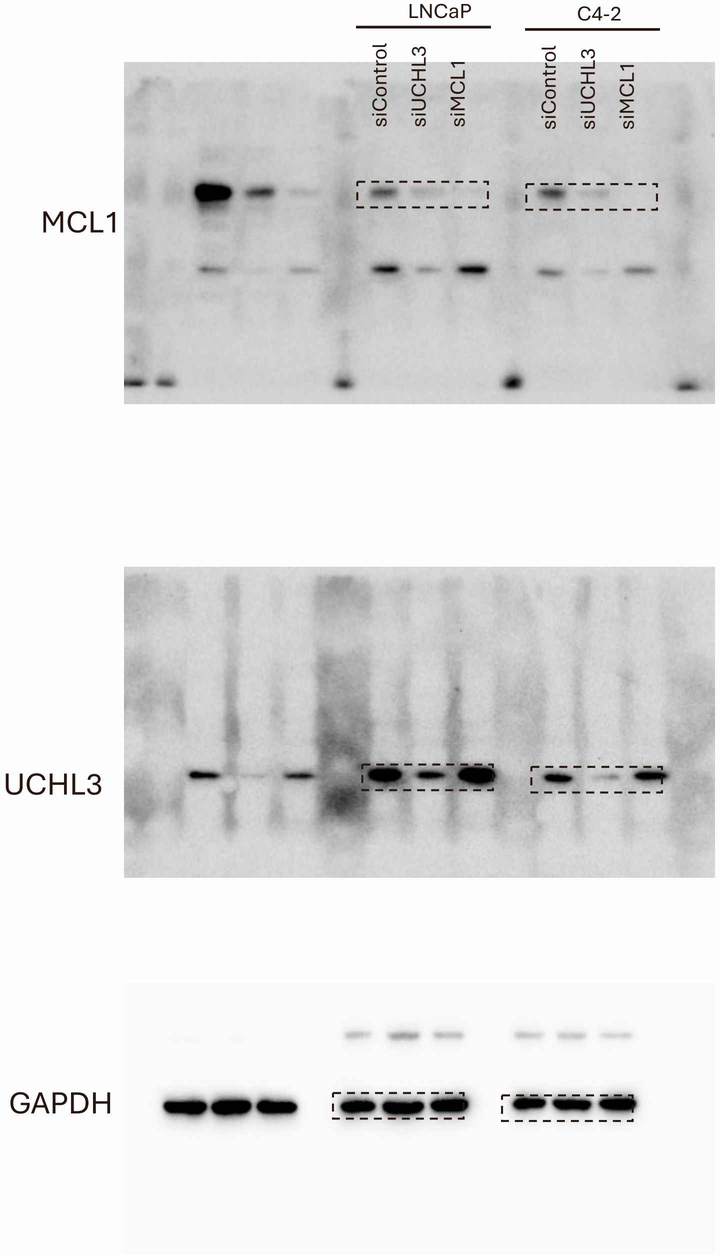

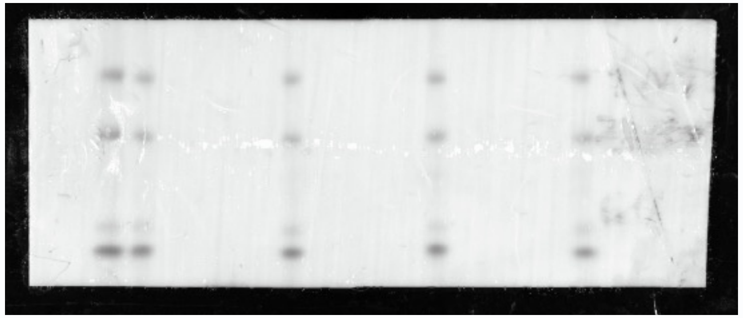

Image not available

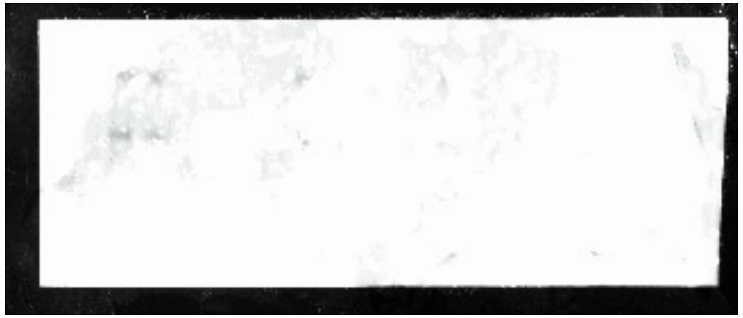

Fig 3B – DU145

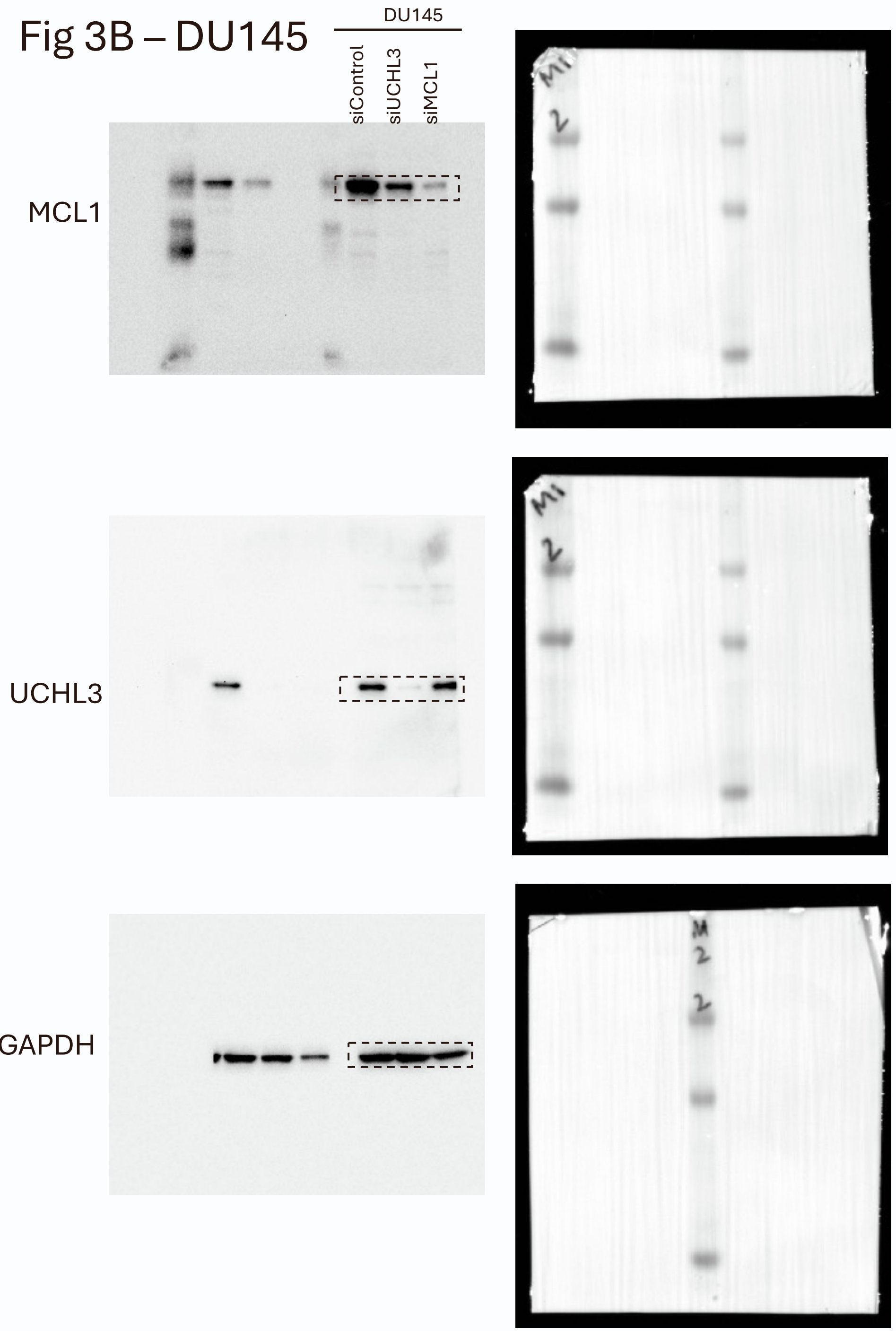

Vinculin
(not shown but done as loading control on membrane M1)

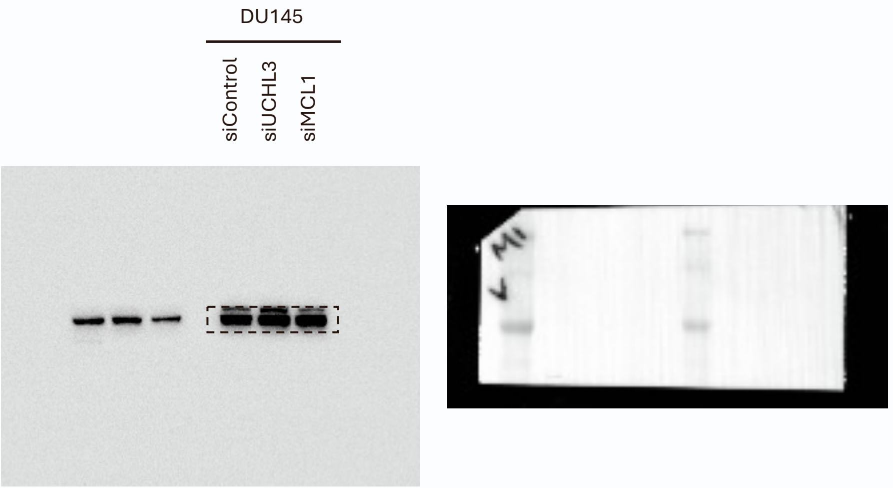

Fig 3B – VCaP

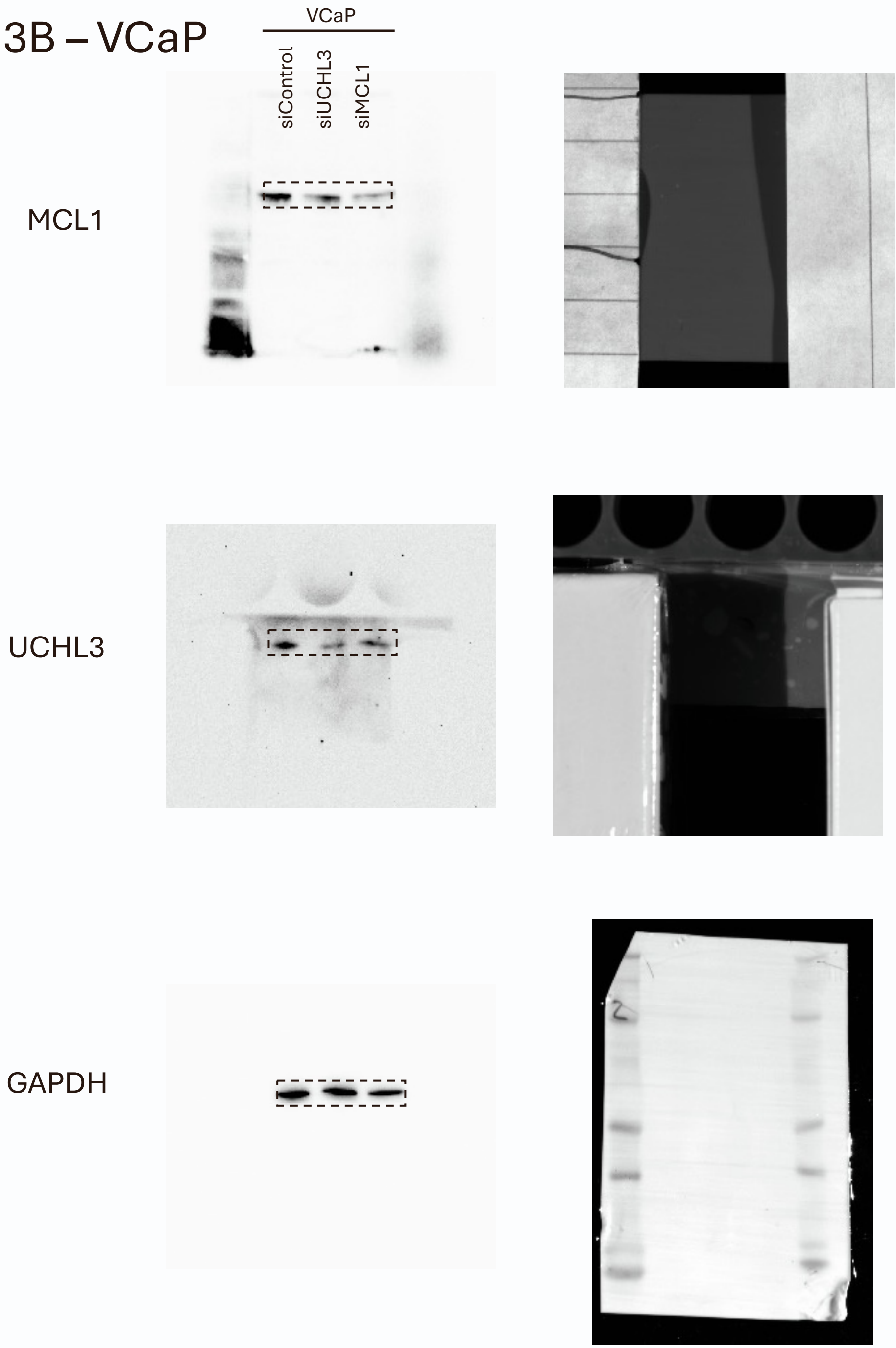

Fig 3D

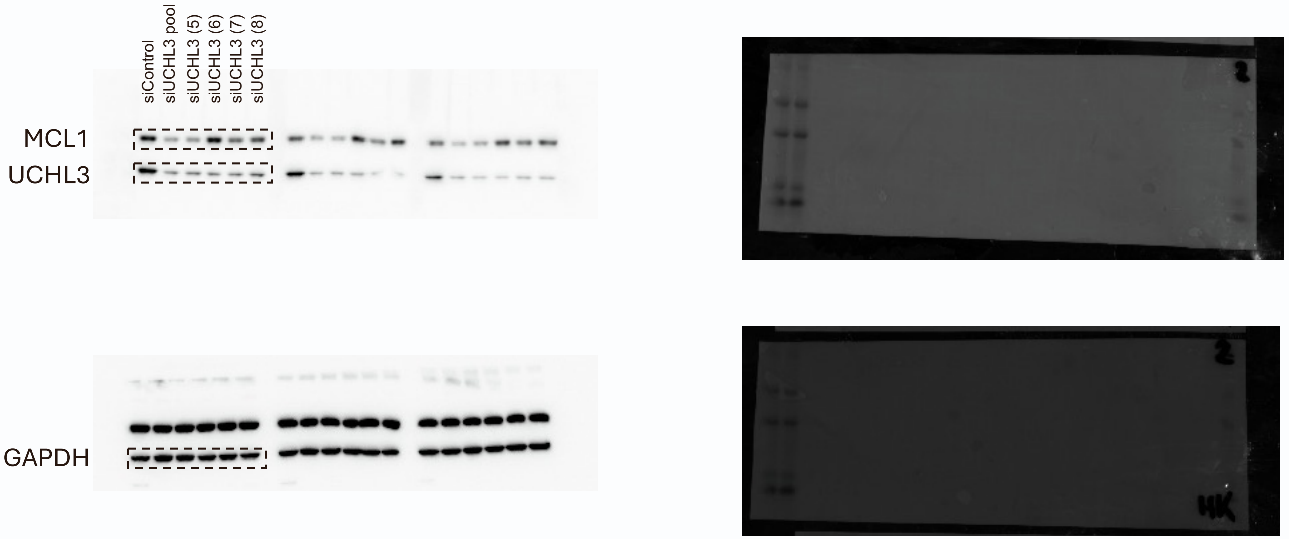
siControl
siUCHL3 pool
siUCHL3 (5)
siUCHL3 (6)
siUCHL3 (7)
siUCHL3 (8)
MCL1
UCHL3
GAPDH

Fig 3E

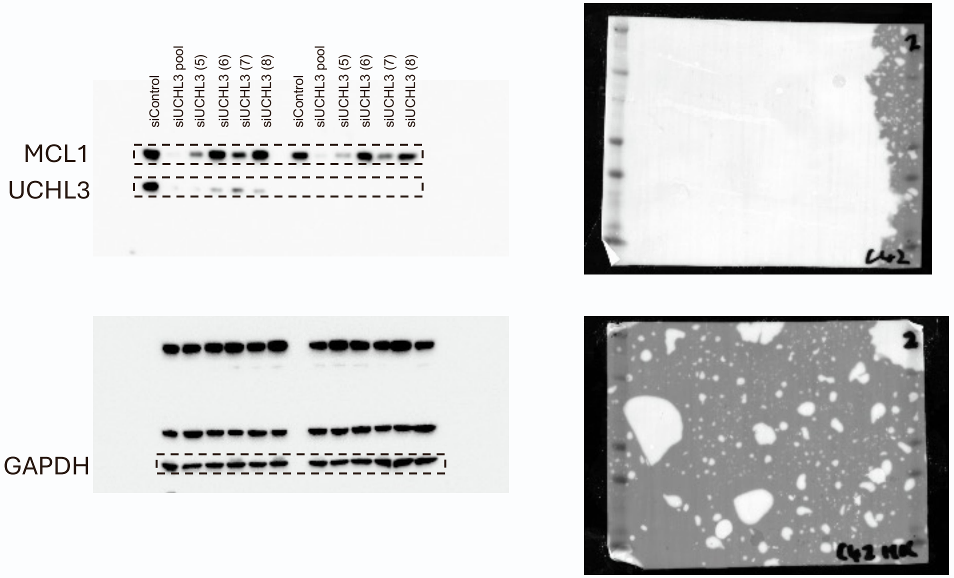
siControl
siUCHL3 pool
siUCHL3 (5)
siUCHL3 (6)
siUCHL3 (7)
siUCHL3 (8)
siControl
siUCHL3 pool
siUCHL3 (5)
siUCHL3 (6)
siUCHL3 (7)
siUCHL3 (8)
MCL1
UCHL3
GAPDH

Fig 3F

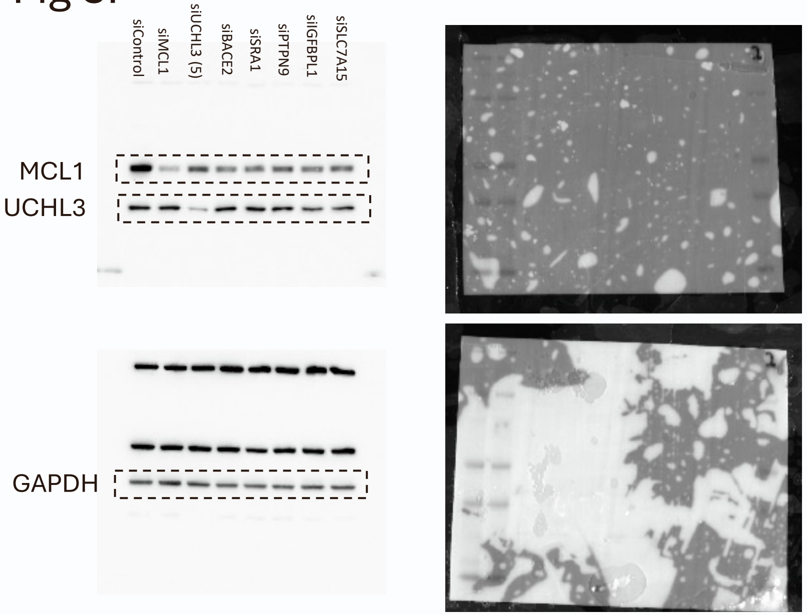
siControl
siMCL1
siUCHL3 (5)
siBACE2
siSRA1
siPTPN9
siIGFBPL1
siSLC7A15
MCL1
UCHL3
GAPDH

Supp Fig 3

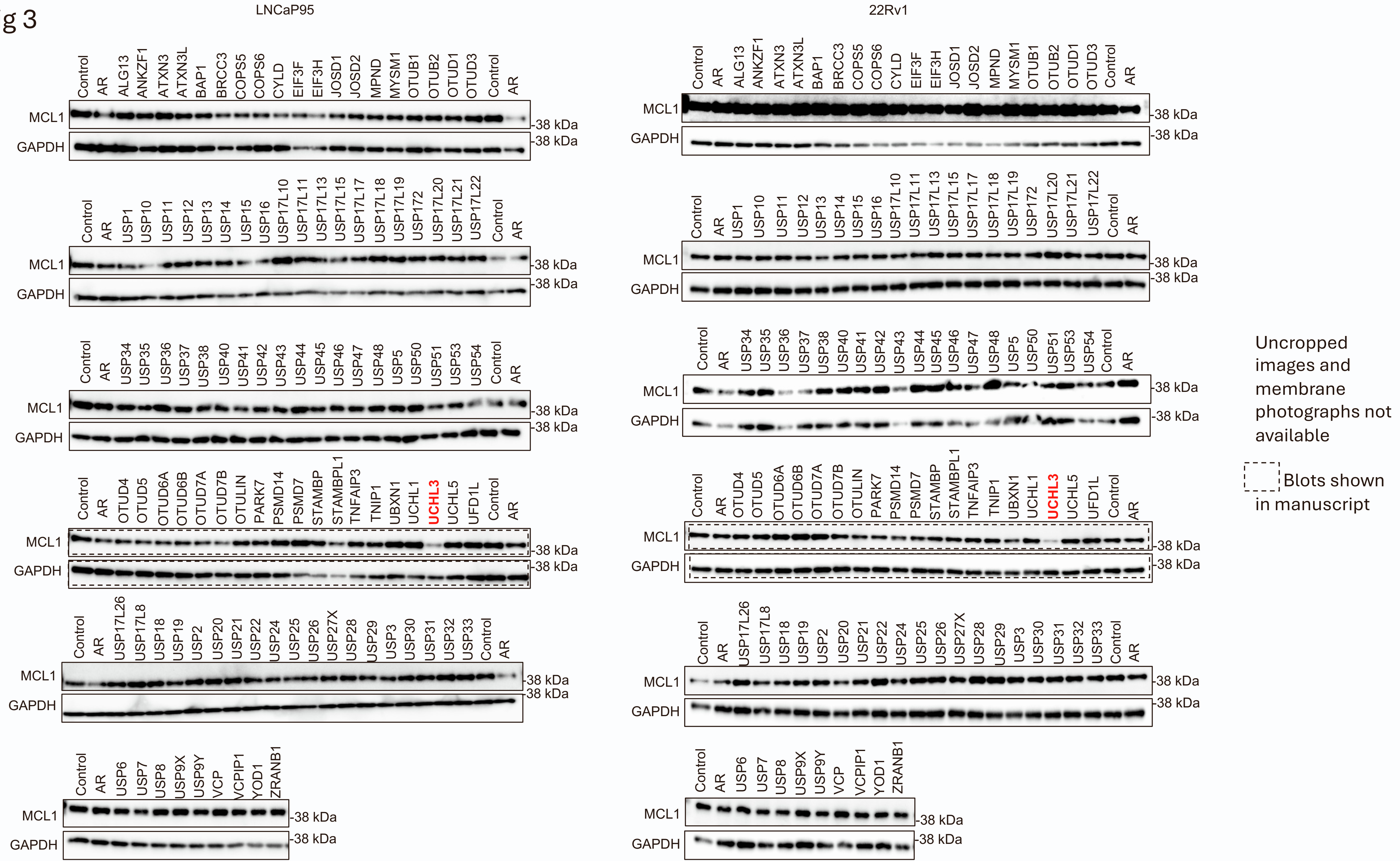
LNCaP95
22Rv1
Control AR ALG13 ANKZF1 ATXN3 ATXN3L BAP1 BRCC3 COPS5 COPS6 CYLD EIF3F EIF3H JOSD1 JOSD2 MPND MYSM1 OTUB1 OTUB2 OTUD1 OTUD3 Control AR
Control AR USP1 USP10 USP11 USP12 USP13 USP14 USP15 USP16 USP17L10 USP17L11 USP17L13 USP17L15 USP17L17 USP17L18 USP17L19 USP172 USP17L20 USP17L21 USP17L22 Control AR
Control AR USP34 USP35 USP36 USP37 USP38 USP40 USP41 USP42 USP43 USP44 USP45 USP46 USP47 USP48 USP5 USP50 USP51 USP53 USP54 Control AR
Control AR OTUD4 OTUD5 OTUD6A OTUD6B OTUD7A OTUD7B OTULIN PARK7 PSMD14 PSMD7 STAMBP STAMBPL1 TNFAIP3 TNIP1 UBXN1 UCHL1 UCHL3 UCHL5 UFD1L Control AR
Control AR USP17L26 USP17L8 USP18 USP19 USP2 USP20 USP21 USP22 USP24 USP25 USP26 USP27X USP28 USP29 USP3 USP30 USP31 USP32 USP33 Control AR
Control AR USP6 USP7 USP8 USP9X USP9Y VCP VCPIP1 YOD1 ZRANB1
MCL1
GAPDH
-38 kDa
Uncropped images and membrane photographs not available
Blots shown in manuscript

Supp Fig 4A

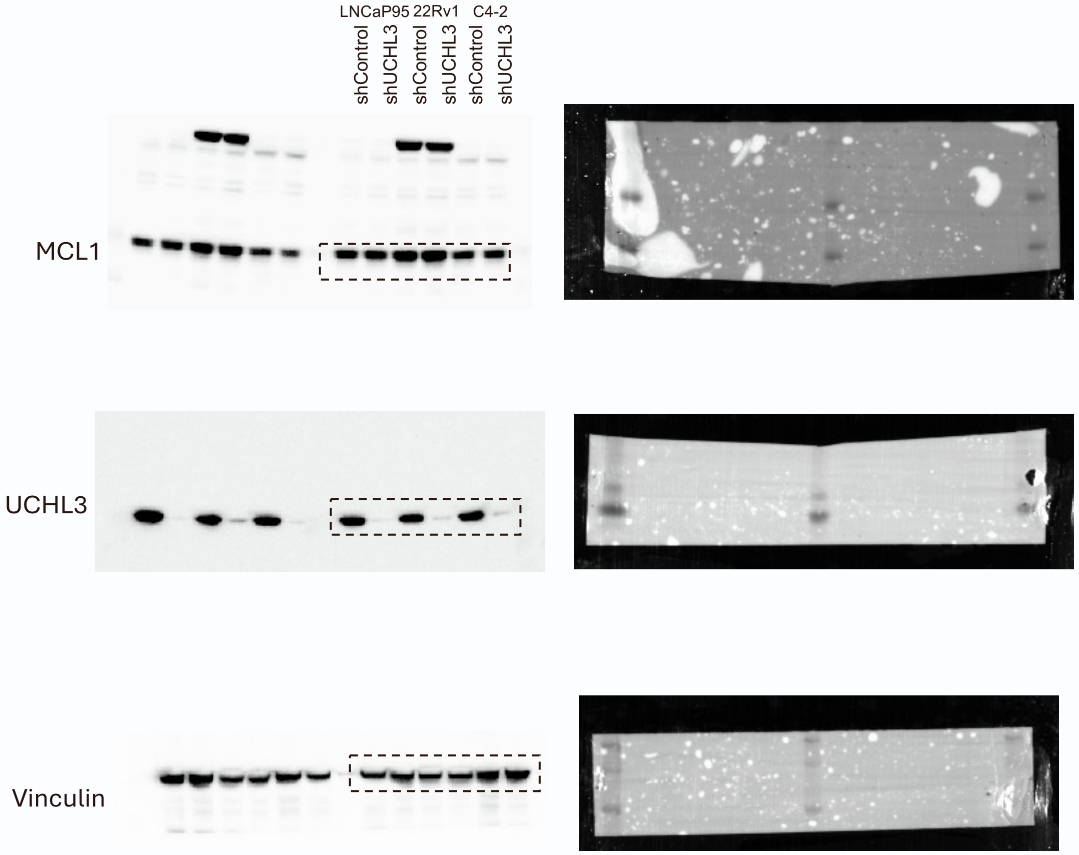
LNCaP95
22Rv1
C4-2
shControl
shUCHL3
shControl
shUCHL3
shControl
shUCHL3
MCL1
UCHL3
Vinculin

Supp Fig 4B

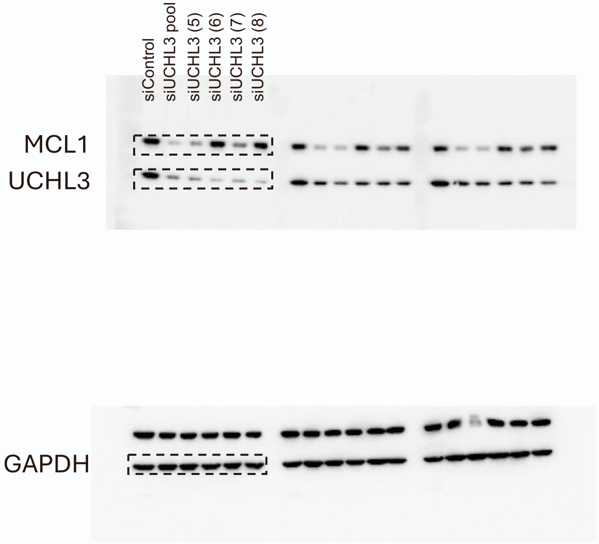
siControl
siUCHL3 pool
siUCHL3 (5)
siUCHL3 (6)
siUCHL3 (7)
siUCHL3 (8)
MCL1
UCHL3
GAPDH

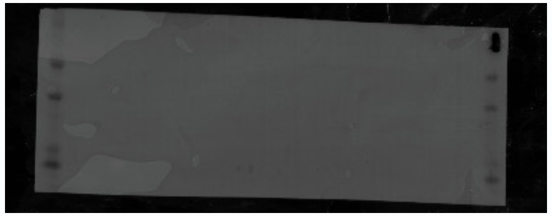

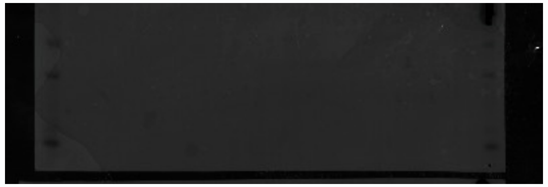

Supp Fig 4C

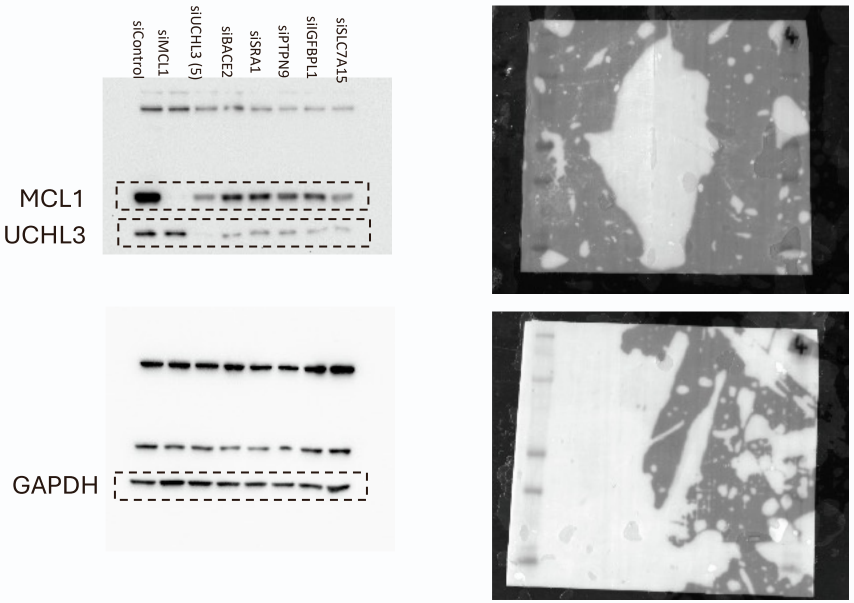
siControl
siMCL1
siUCHL3 (5)
siBACE2
siSRA1
siPTPN9
siIGFBPL1
siSLC7A15
MCL1
UCHL3
GAPDH

Supp Fig 5F

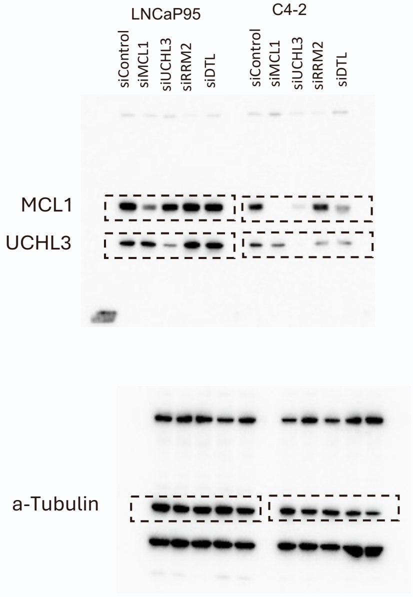

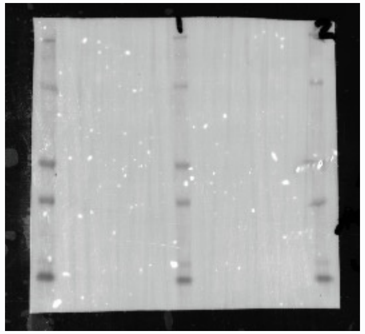

Image not available

Supp Fig 5G

MCL1

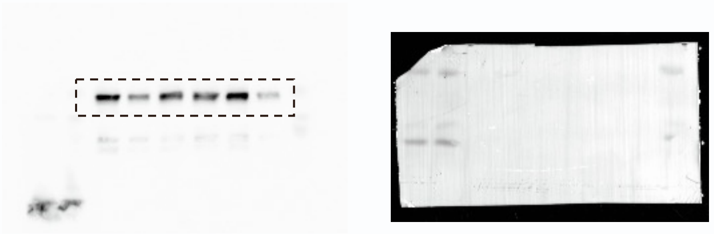

DTL

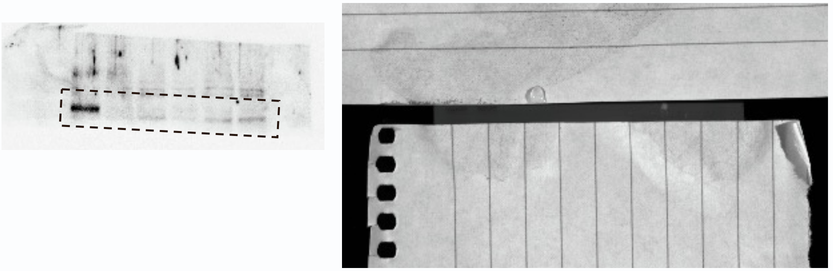

a-Tubulin

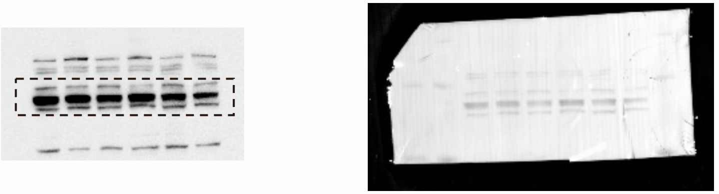

Supp Fig 6

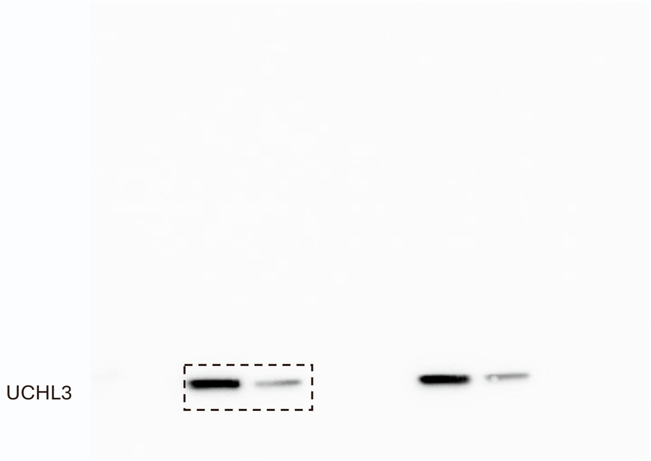

Image not available

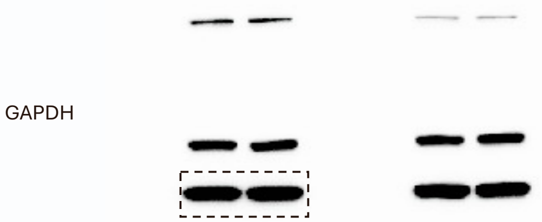

Image not available

Fig 5B

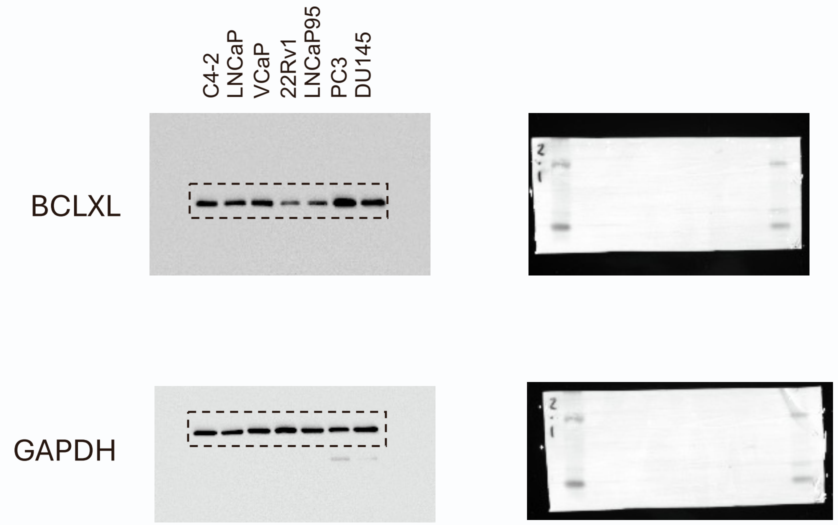

Fig 5F

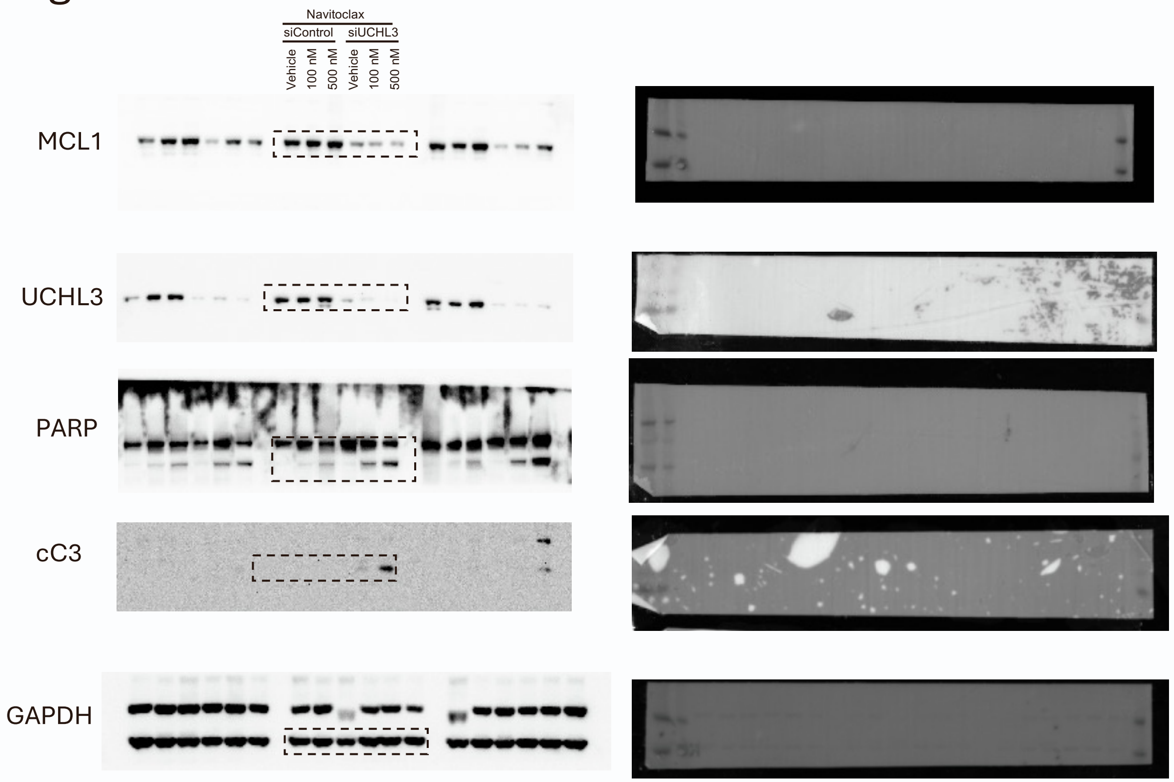
Navitoclax
siControl
siUCHL3
Vehicle
100 nM
500 nM
Vehicle
100 nM
500 nM
MCL1
UCHL3
PARP
cC3
GAPDH

Supp Fig 7C – AZD4320

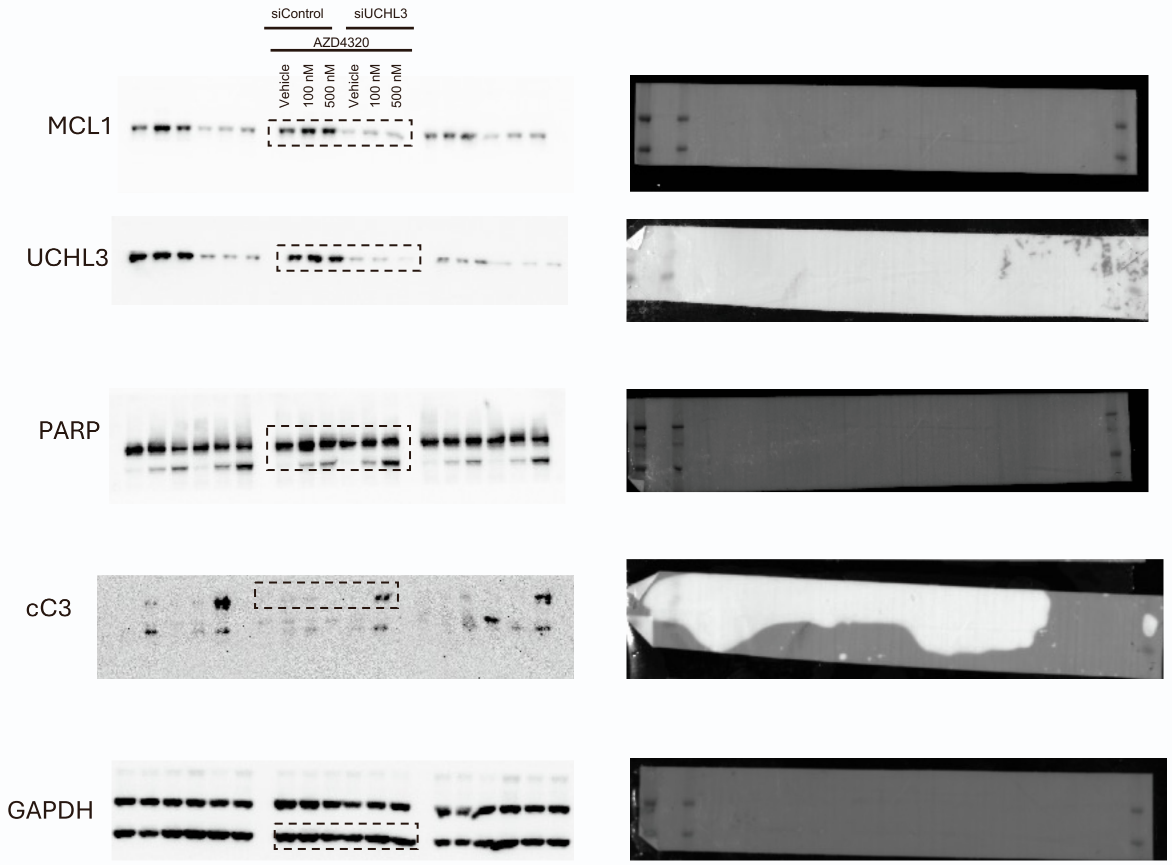

Supp Fig 7C – A-1331852

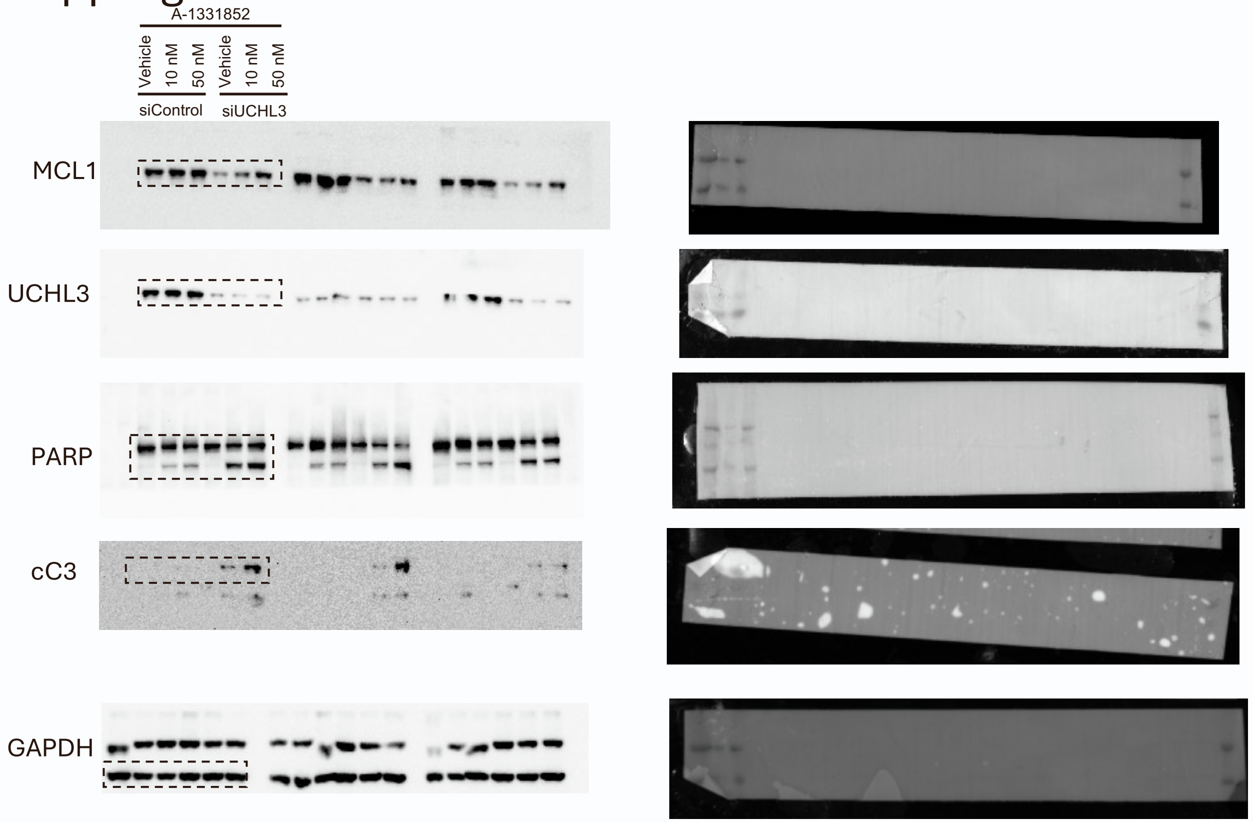

Supp Fig 8C

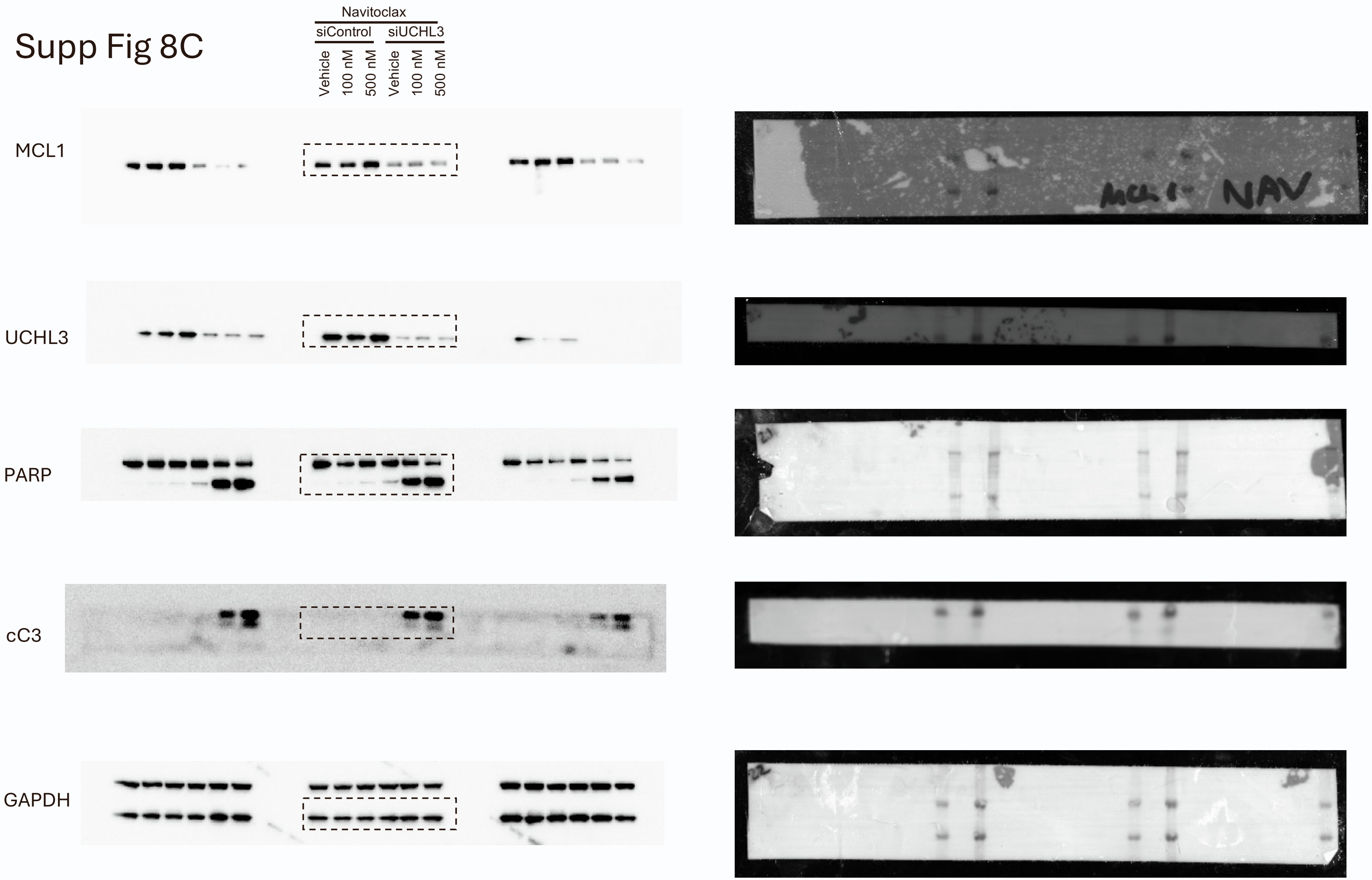
Navitoclax
siControl
siUCHL3
Vehicle
100 nM
500 nM
Vehicle
100 nM
500 nM
MCL1
UCHL3
PARP
cC3
GAPDH
MCL1
NAV

Supp Fig 8C – AZD4320

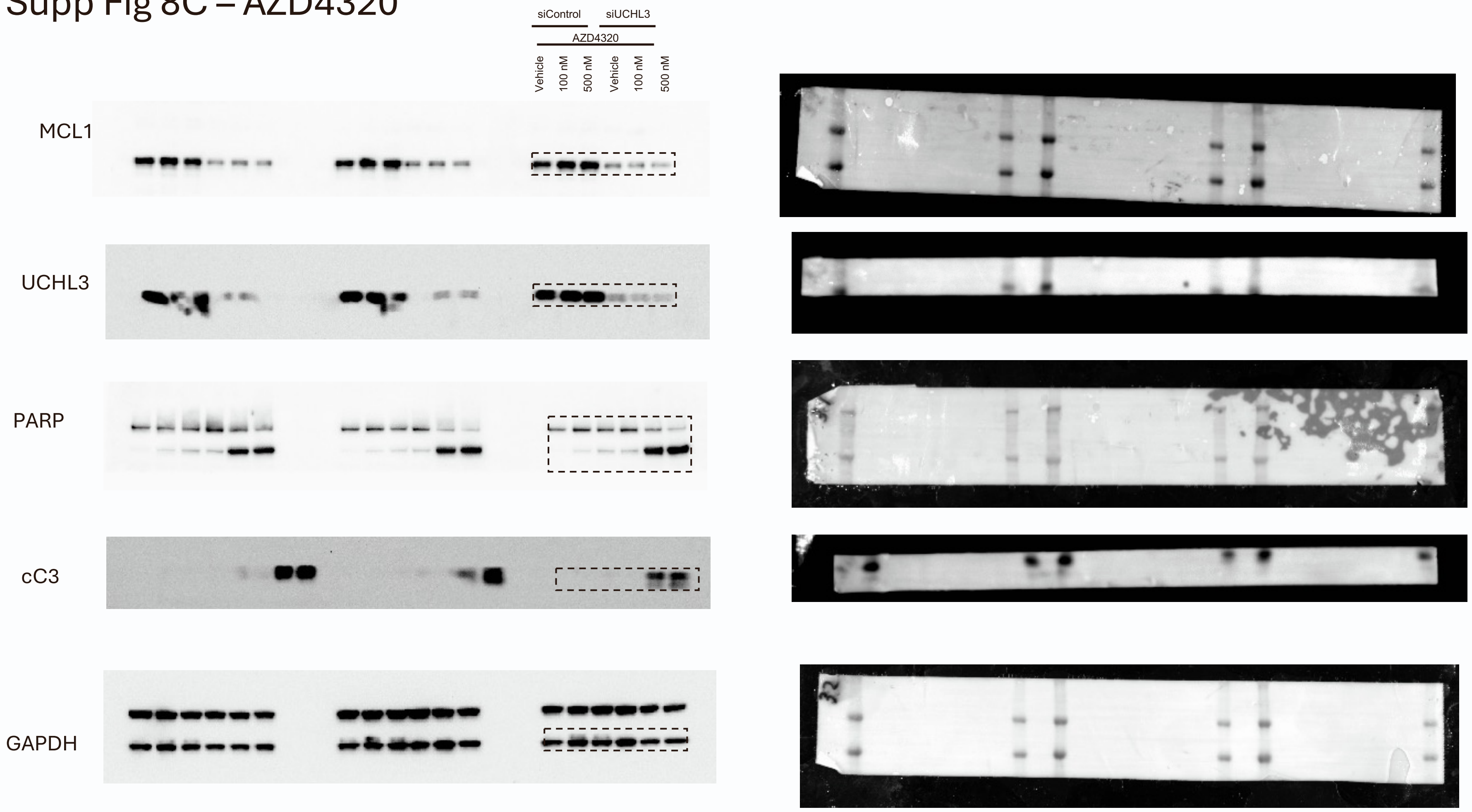

Supp Fig 8C – A-1331852

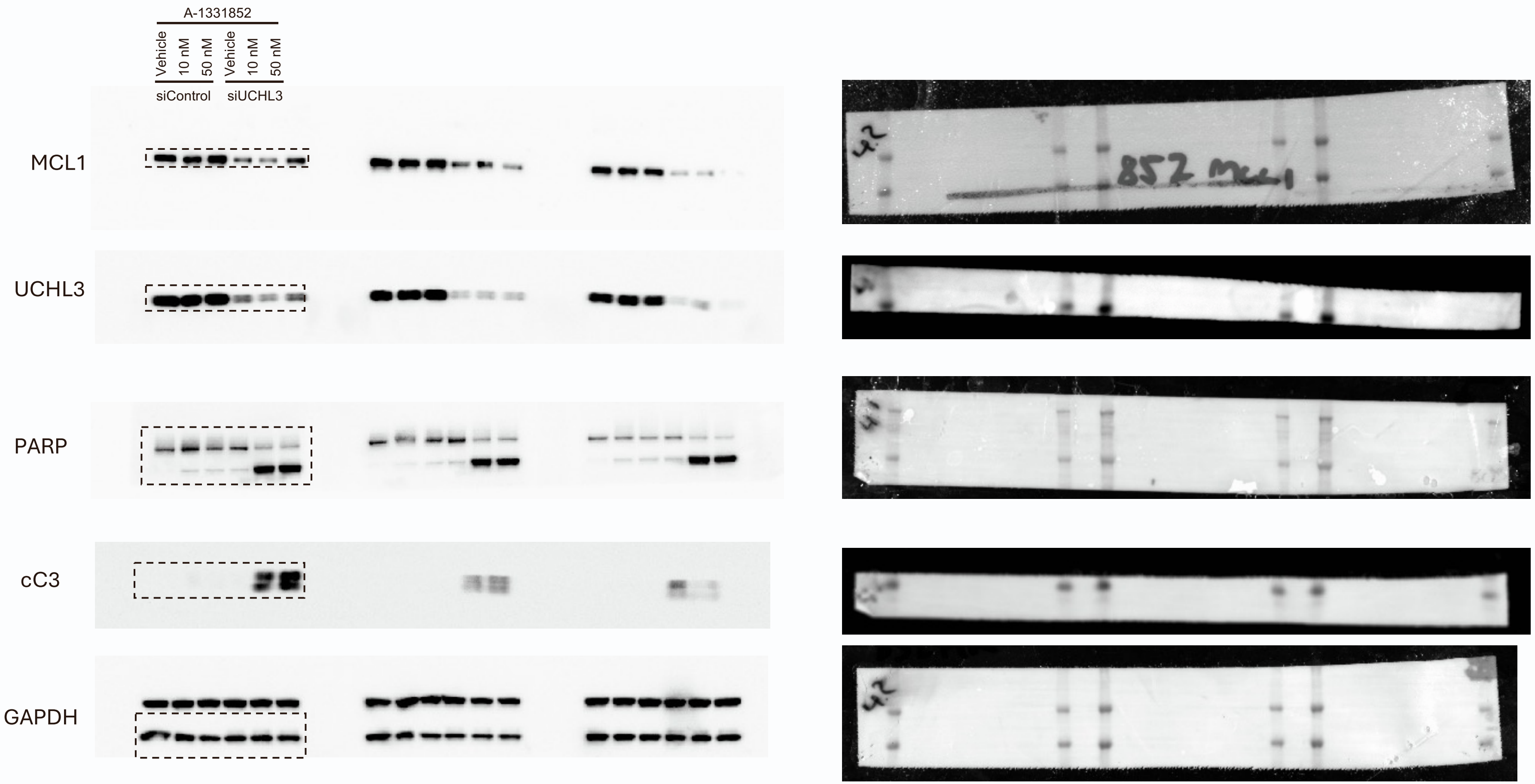

Supp Fig 8C – A-1331852

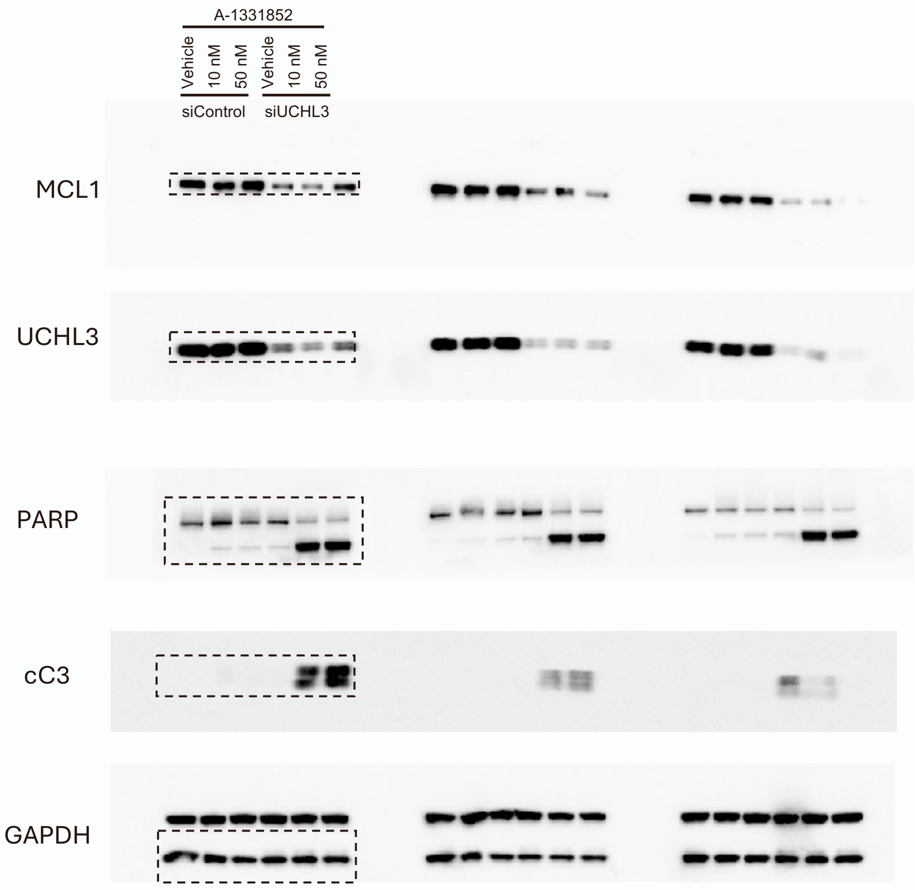

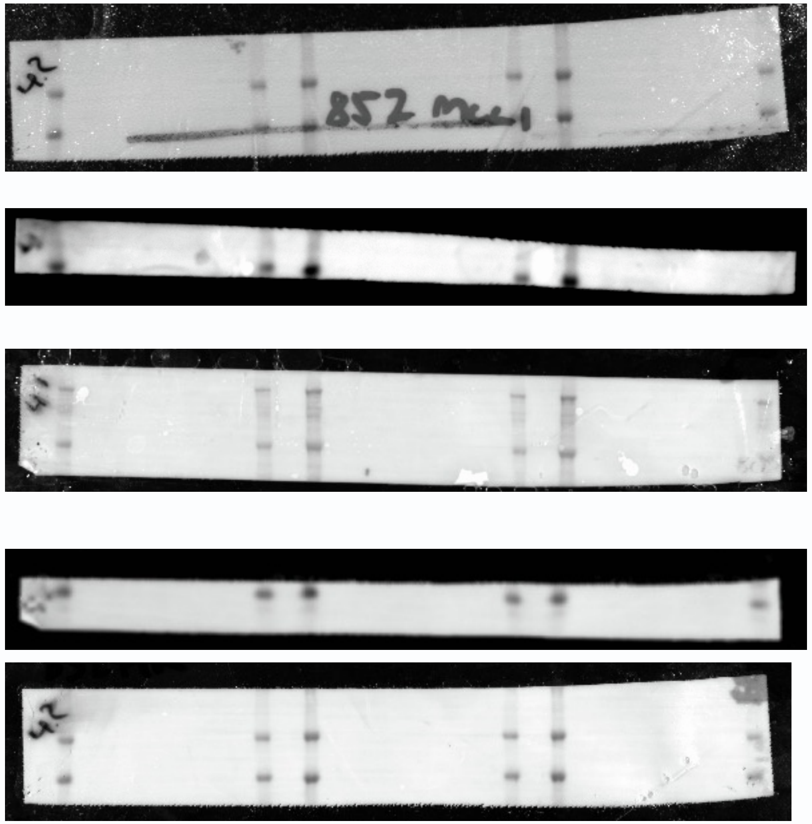

Supp Fig 8D

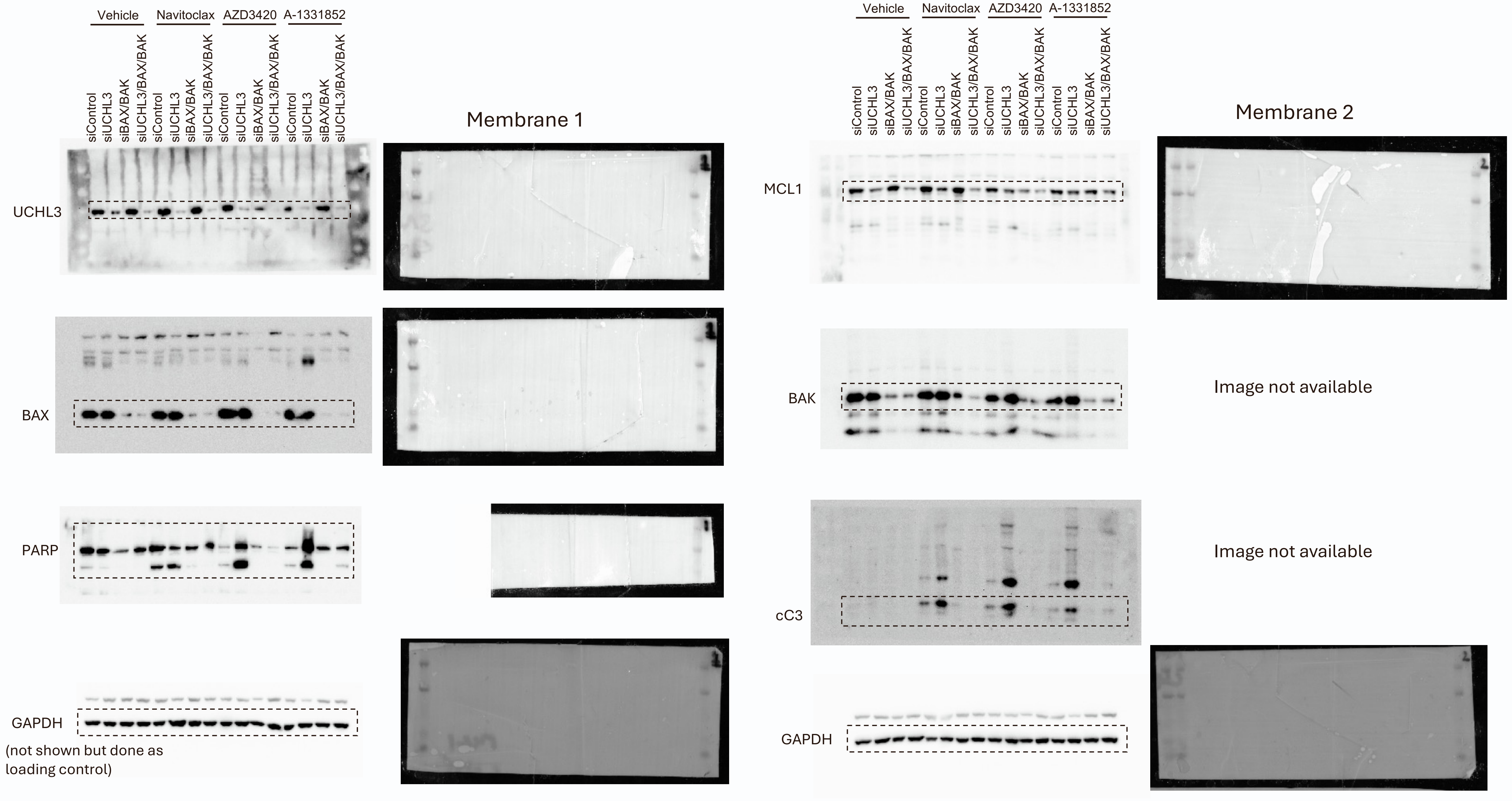

Vehicle
Navitoclax
AZD3420
A-1331852
siControl
siUCHL3
siBAX/BAK
siUCHL3/BAX/BAK
Membrane 1
UCHL3
BAX
PARP
GAPDH
(not shown but done as loading control)
Vehicle
Navitoclax
AZD3420
A-1331852
siControl
siUCHL3
siBAX/BAK
siUCHL3/BAX/BAK
Membrane 2
MCL1
BAK
Image not available
cC3
Image not available
GAPDH

Supp Fig 8E

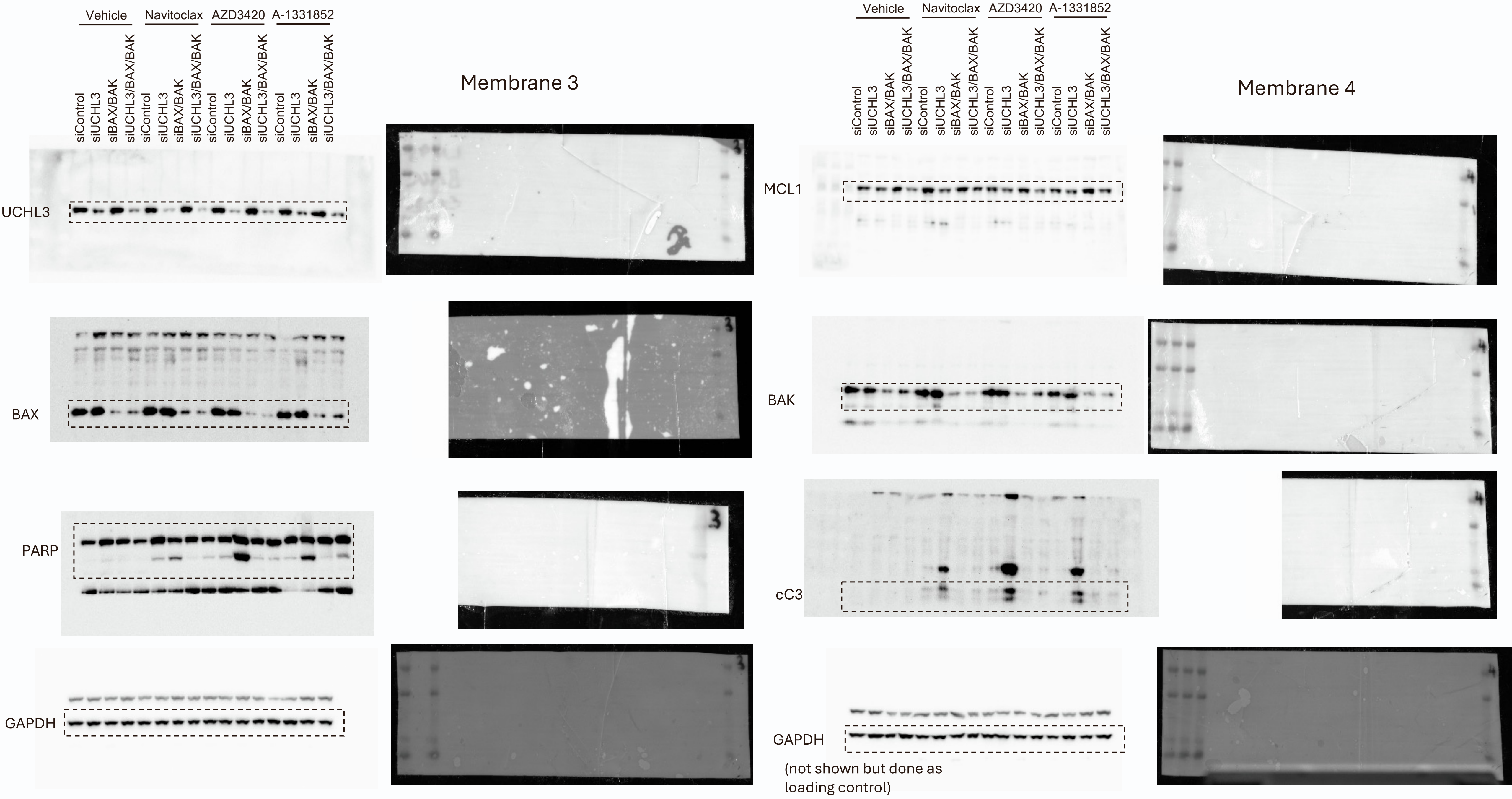
Vehicle
Navitoclax
AZD3420
A-1331852
siControl
siUCHL3
siBAX/BAK
siUCHL3/BAX/BAK
Membrane 3
UCHL3
BAX
PARP
GAPDH
MCL1
BAK
cC3
GAPDH
(not shown but done as loading control)
Membrane 4
